# Supplementary material for: Choice architecture interventions to improve diet and/or dietary behaviour by healthcare staff in high-income countries: a systematic review
Source: BMJ Open. 2019 Jan 23;9(1):e023687. doi: 10.1136/bmjopen-2018-023687 (PMC6347858; doi:10.1136/bmjopen-2018-023687)
Supplement: Supplementary file 1 [file bmjopen-2018-023687supp001.pdf]

Search strategy used to interrogate MEDLINE

#	Searches	# of hits
35	limit 34 to ("all adult (19 plus years)" and humans)	2083
34	7 and 25 and 31	3734
33	limit 32 to ("all adult (19 plus years)" and humans)	4814
32	7 and 25 and 30	8677
31	26 or 27 or 28	2095764
30	26 or 27 or 28 or 29	2837423
29	health care.mp. or exp "Delivery of Health Care"/	1267298
28	exp Workplace/	17804
27	education, public health professional/ or exp "organization and administration"/	1248979
26	hospital*.mp.	1229950
25	18 or 24	537980
24	19 or 20 or 21 or 22 or 23	532969
23	eating behaviour.mp.	1262
22	Food habit*.mp.	1705
21	exp nutrition surveys/ or exp diet surveys/	21175
20	exp Nutrition Assessment/ or nutrition.mp. or exp Nutrition Surveys/	185218
19	exp Diet/ or Diet.mp.	396453
18	11 and 17	7544
17	12 or 13 or 14 or 15 or 16	1323348
16	drink*.mp.	153981
15	exp Meals/	3251
14	exp Food Preferences/	11931
13	exp beverages/ or exp food/	1173261
12	purchas*.mp.	26716
11	8 or 9 or 10	27711
10	Food Dispensers, Automatic/ or vending machine.mp.	388
9	sale*.mp.	14446
8	exp food services/ or exp food service, hospital/	13308
7	1 or 2 or 3 or 4 or 5 or 6	311674
6	choice architecture.mp.	42
5	exp Self Stimulation/ or exp Choice Behavior/ or exp Decision Making/ or nudge.mp.	173309
4	lifestyle intervention*.mp.	4193
3	exp life style/ or healthy lifestyle/	78107
2	behavio?r intervention*.mp.	528
1	exp health promotion/ or exp healthy people programs/ or weight reduction programs/	66897
