# Supplementary material for: Choice architecture interventions to improve diet and/or dietary behaviour by healthcare staff in high-income countries: a systematic review
Source: BMJ Open. 2019 Jan 23;9(1):e023687. doi: 10.1136/bmjopen-2018-023687 (PMC6347858; doi:10.1136/bmjopen-2018-023687)
Supplement: Supplementary file 2 [file bmjopen-2018-023687supp002.pdf]

**Which outcome?**	purchasing/dietary consumption/both *[delete as appropriate]*
**Reference (complete reference)**	
**Study ID (first author, year)**	
**Endnote No.**	
**Other reports of this trial: (Give EN No. First Author and Year)**	
**Reviewer:**	LA OO OU *[delete as appropriate]*
**Date of abstraction:**	

For studies awaiting classification skip results and quality assessment

For ongoing studies skip to the end of this form

Publication details

	[delete as appropriate]
**Language of publication**	English
**Funding source**	Commercial funding / non-commercial funding / other funding (specify)
**Publication status**	peer-reviewed journal / journal supplement / full article / other (specify)

Stated aims of study

**Quote from publication: "..."**	

**Notes**	

INFORMATION FOR CHARACTERISTICS OF INCLUDED STUDIES TABLE

	[delete as appropriate]
**Study design**	
**Participants:**	
**Brief description of participants/worksite**	
Inclusion criteria	
Exclusion criteria	
**Intervention:**	
**Number of worksites**	
**Country/authors location**	
**Setting (e.g. primary care, hospital)**	
**Type of health care setting** (such as NHS vs. non-NHS)	

**Description of intervention** (give details of all components)	
**Type of intervention (highlight and describe)**	Choice architecture only/choice architecture + complex intervention
**Select intervention type**	
**Primarily alter properties or objective stimuli**	Ambiance – alter aesthetic or atmospheric aspects of the surrounding environment
	Functional design – design or adapt equipment or function of the environment
	Labelling – apply labelling or endorsement information to product or at point of choice
	Presentation – alter sensory qualities or visual design of the product
	Sizing – change size or quantity of the product
	Pricing- change price of the product
**Primarily alter placement or objects or stimuli**	Availability – add behavioural options within a given micro-environment
	Proximity – make behavioural options easier (or harder) to engage with, requiring reduced (or increased) effort
**Alter both properties and placement of objects or stimuli**	Priming – place incidental cues in the environment to influence a non-conscious behavioural response
	Prompting – use non-personalised information to promote or raise awareness of a behaviour
**Restaurant/canteen/vending machine/other (specify)**	
**Duration of intervention (duration of follow-up)**	Intervention:<br><br>Follow-up:
**Comparator**	Yes/no
**Type of comparator**	No intervention/wait list, different intervention, concomitant intervention, other ( )
**Description of comparator**	
**Pre-intervention setup**	Yes/no
**Description of pre-intervention setup**	
**Length of pre-intervention**	

OUTCOMES

NB: subjective measures of dietary intake **validated measures are eligible**. Any other measures list and note not data extracted.

**Tools description**	
**Dietary intake**	
**Dietary tool**	
**Purchasing data details**	

Baseline Characteristics

	**Intervention 1**	**Intervention 2**	**Comparator 1**	**Comparator 2**	**Overall**
**Sample size**					
**Staff group** (grade and/or category eg: medical vs nursing vs administrative)					
**Number of (*item*) sold**					
**Type of (*item*) sold** *[insert rows as needed]*					
**Type of (*item*) sold**					
**Type of (*item*) sold**					
**Price of (item) sold** *[insert rows as needed]*					
**Price of (item) sold** *[insert rows as needed]*					

Purchasing data *[insert item name, add rows if required]*	**Intervention 1**	**Intervention 2**	**Comparator 1**	**Comparator 2**	***P values***
**Outcomes**					
**Sales data (%/n):** specify item					
At time point (specify)					
**Sales data (%/n):** specify item					
At time point (specify)					
**Sales data (%/n):** specify item					
At time point (specify)					
**Sales data (%/n):** specify item					
At time point (specify)					
**Sales data (%/n):** specify item					
At time point (specify)					
**Sales data (%/n):** specify item					
At time point (specify)					
**Narrative findings (please add *p* values):**					

Dietary data (do not extract total caloric intake, *insert item name, add rows if required]*

**Outcomes**	**Intervention 1**	**Intervention 2**	**Comparator 1**	**Comparator 2**
**Dietary outcomes** (specify, unit)				
Baseline	(mean, SD, N)			
At time point (specify)				
Change from baseline (specify which time point) reviewer to calculate if not reported[a]				
**Dietary outcomes** (specify, unit), mean (SD), number of participants:	(mean, SD, N)			
Baseline				
At time point (specify)				
Change from baseline (specify which time point) reviewer to calculate if not reported[a]				
**Dietary outcomes** (specify, unit), mean (SD), number of participants:	(mean, SD, N)			
Baseline				
At time point (specify)				
Change from baseline (specify which time point) reviewer to calculate if not reported[a]				
**Narrative findings (please add *p* values):**				

Notes of data to convert/calculations to be made (e.g. SEM to SD, change data, unavailable date):

Survey of study investigators providing information on included trials

**Study author contacted**<br><br>**[DD/MM/YY]**	
**Study author replied**<br><br>**[DD/MM/YY]**	

**Study author asked for additional information**<br><br>**[short summary]**	
**Study author provided data**<br><br>**[short summary]**	

RISK OF BIAS

**Risk of bias for RCTs**	*[delete as appropriate]*	**Support for judgement**
**Random sequence generation? (selection bias)**	Low risk of bias / High risk of bias / Unclear	Quote from publication: "..."
**Allocation concealment? (selection bias)**	Low risk of bias / High risk of bias / Unclear	Quote from publication: "..."
**Blinding of participants and personnel? (performance bias) Subjective outcomes**	Low risk of bias / High risk of bias / Unclear	Quote from publication: "..."
**Blinding of participants and personnel? (performance bias) Objective outcomes**	Low risk of bias / High risk of bias / Unclear	Quote from publication: "..."
**Blinding of outcome assessment? (detection bias) Subjective outcomes**	Low risk of bias / High risk of bias / Unclear	Quote from publication: "..."
**Blinding of outcome assessment? (detection bias) Objective outcomes**	Low risk of bias / High risk of bias / Unclear	Quote from publication: "..."
**Incomplete outcome data? (attrition bias) Subjective outcomes**	Low risk of bias / High risk of bias / Unclear	Quote from publication: "..."<br><br>Comment: no missing data / reported and reasons explained / only reported / not reported
**Incomplete outcome data? (attrition bias) Objective outcomes**	Low risk of bias / High risk of bias / Unclear	Quote from publication: "..."<br><br>Comment: no missing data / reported and reasons explained / only reported / not reported
**Selective outcome reporting?**	Low risk of bias / High risk of bias / Unclear	Comment
**Other bias?**	Low risk of bias / High risk of bias / Unclear	Comment

**Risk of bias for non-RCTs**	*[delete as appropriate]*	**Support for judgement**
**Selection of participants:** *Selection bias caused by the inadequate selection of participants*	Low risk of bias / High risk of bias / Unclear	Quote from publication: "..."<br><br>Comment
**Confounding variables:** *Selection bias caused by the inadequate confirmation and consideration of confounding variable*	Low risk of bias / High risk of bias / Unclear	Quote from publication: "..."<br>Comment
**Measurement of exposure:** *Performance bias caused by the inadequate measurement of exposure*	Low risk of bias / High risk of bias / Unclear	Quote from publication: "..."<br><br>Comment
**Blinding of outcome assessments (subjective outcomes)** *Detection bias caused by the inadequate blinding of outcome assessments*	Low risk of bias / High risk of bias / Unclear	Quote from publication: "..."<br><br>Comment
**Blinding of outcome assessments (objective outcomes)** *Detection bias caused by the inadequate blinding of outcome assessments*		
**Incomplete outcome data (subjective outcomes)** *Attrition bias caused by the inadequate handling of incomplete outcome data*	Low risk of bias / High risk of bias / Unclear	Quote from publication: "..."<br><br>Comment
**Incomplete outcome data (objective outcomes)** *Attrition bias caused by the inadequate handling of incomplete outcome data*		
**Selective outcome reporting:** *Reporting bias caused by the selective reporting of outcomes*	Low risk of bias / High risk of bias / Unclear	Quote from publication: "..."<br><br>Comment

<u>Ongoing studies</u>

Behavioural interventions to increase healthier purchasing and/or consumption of food and drink data abstraction form

**Study name**	Acronym:	
**Methods**	Study design:	
	Type of health care setting:	
	Country:	
**Participants**	Enrollment: (e.g. estimated 40)	
	Inclusion criteria:	
	Exclusion criteria:	
**Interventions**	Intervention(s):	
	Comparator(s):	
**Outcomes**	OUTCOME(S)	
**Starting date**	Study start date:	
	Study completion date:	
**Contact information**	Responsible party/principal investigator:	
**Study identifier** (if applicable)	(e.g.) NCT number:	
**Official title**		
**Stated purpose of study** (aim)	Quote: "..."	
**Notes**		
