# Supplementary material for: Choice architecture interventions to improve diet and/or dietary behaviour by healthcare staff in high-income countries: a systematic review
Source: BMJ Open. 2019 Jan 23;9(1):e023687. doi: 10.1136/bmjopen-2018-023687 (PMC6347858; doi:10.1136/bmjopen-2018-023687)
Supplement: Supplementary file 3 [file bmjopen-2018-023687supp003.pdf]

Evidence-based environmental changes to promote healthy eating and drinking by NHS staff

The size of the problem

- The leading risk behaviour for disability and death in England is poor diet[1].
- Disease associated with poor diet costs the NHS at least £5.8 billion per year[2].
- Nearly two thirds of adults in England are now overweight or obese[3].
- The overall cost of obesity to wider society was projected to reach £27 billion in 2015[4].

Barriers to healthy eating specifically reported by health care staff [13,14,15,16,17]

- Lack of time;
- Unpredictable/demanding workload;
- Prioritising patients workload;
- Inconvenient access to food (both physically ie: a long walk to where food is served, and in time ie: 24 hour duties but limited opening times.
- Lack of availability of food they want to eat.

The importance to the NHS

- The NHS is the fifth largest employer on the planet, and the largest employer in the UK[5]. Improving the health and wellbeing of the 1.4 million NHS staff will lift the health and wellbeing levels of the entire UK population.
- Obesity is associated with absenteeism, presenteeism, early retirement, injuries, discrimination, and litigation[6,7,8]. NHS staff absence due to poor health costs at least £2.4billion a year[9]. Improving employee health will improve productivity and performance of the workforce leading to improved patient care.
- Health professionals play an important role in health promotion for the 1 million patients seen in the NHS every 36 hours. Many staff feel more able to do this when they appear to be following health advice themselves[10,11]. By taking action to address diet and obesity for NHS staff, the organisation and staff will be role models for others.
- Policy drivers include NICE Guidance on obesity prevention in workplaces (CG43); NHS standard contract; NHS CQUIN Indicator: Healthy food for NHS staff, visitors and patients; NHS England voluntary sugar-sweetened beverage target [Appendix 1].

Evidence based environmental changes that work to improve diets

Changing the environment to promote healthy behaviour- such as healthy food and drink purchasing and consumption can be particularly useful and effective because:

- When the environment makes healthy choices easier, it doesn't require any effort by the target audience. This means it isn't just those people who are highly motivated to make healthy changes who benefit.
- Most changes are no-cost or low cost to introduce.

Providing information alone (for example, labelling food to indicate that it is healthy or its calorie content) is unlikely to improve diet, although this can work alongside other environmental changes.

More benefits to making these changes

- Greater availability of healthy food can improve the general attractiveness of a food provider[12].Customer volume and sales may increase after changes to improve healthy food and drink purchasing are made[13,14] Types of change for which there is evidence of effectiveness:

Type of change	Examples
Reduce the effort required to select healthy options, or increase the effort required to select unhealthy options.	➢ Healthier vegetable spread available in easy-to-reach positions, butter stored in a fridge.<br>➢ Desserts arranged so that fresh fruit salad was situated in the easiest access positions, cakes and pies in the least convenient.<br>➢ Rearrangement of the canteen to increase the prominence of fruit and water and reduce prominence of confectionery and crisps.
Increase availability of healthy options.	➢ Snacks on offer at a hospital canteen enforced at 75% healthy, no more than 25% unhealthy.<br>➢ Healthy snack cart that visited staff.
Offer smaller sizes of main meals and other products.	➢ Two size options for the main meal offered in a hospital canteen.

Other examples of successful initiatives include:

- An online pre-ordering system for lunch, which allowed staff to choose their lunch an hour or more before collecting it at the canteen without joining a queue. Nutritional information was presented and the system defaulted to the healthier option when appropriate.
- Traffic light labelling of all items on offer, with green (healthy) items placed in the most salient positions and red (unhealthy) items made less visible. Permanent changes to menu boards and other signage to highlight the healthiest options.

References

For more details of the systematic review that supported this document, please contact o.r.o.oyebode@warwick.ac.uk.

1. Newton JN, Briggs AD, Murray CJ, et al. Changes in health in England, with analysis by English regions and areas of deprivation, 1990–2013: a systematic analysis for the Global Burden of Disease Study 2013. *The Lancet* 2015;386:2257-74.
2. Scarborough P, Bhatnagar P, Wickramasinghe KK, et al. The economic burden of ill health due to diet, physical inactivity, smoking, alcohol and obesity in the UK: an update to 2006–07 NHS costs. *J Public Health* 2011;33:527-35.
3. Health and Social Care Information Centre, 2016. Health Survey for England 2015: Adult overweight and obesity. NHS Digital. https://digital.nhs.uk/catalogue/PUB20562 (accessed November 2017).
4. Butland B, Jebb S, Kopelman P, et al. Tackling obesities: future choices-project report. London: Department of Innovation, Universities and Skills 2007; 10:17.
5. Young A. Innovation within a national health care system. *Surgery* 2017;161:1179-82.
6. Shrestha N, Pedisic Z, Neil-Sztramko S, Kukkonen-Harjula KT, Hermans V. The impact of obesity in the workplace: a review of contributing factors, consequences and potential solutions. *Current obesity reports* 2016;5:344-60.
7. Koepp GA, Snedden BJ, Levine JA. Workplace slip, trip and fall injuries and obesity. *Ergonomics* 2015;58:674-79.
8. Flint SW, Snook J. Disability discrimination and obesity: the big questions? *Current obesity reports* 2015;4:504-09
9. Blake H, Mo PK, Lee S, Batt ME. Health in the NHS: lifestyle behaviours of hospital employees. *Perspectives in public health* 2012;132:213.
10. Dalton M. Implementing NICE obesity guidance for staff: an NHS trust audit. *Occup Med* 2014;65:78-85.
11. Van Kleef E, Otten K, van Trijp HC. Healthy snacks at the checkout counter: A lab and field study on the impact of shelf arrangement and assortment structure on consumer choices. *BMC public health* 2012;12:1072.
12. Jilcott Pitts S, Graham J, Mojica A, et al. Implementing healthier foodservice guidelines in hospital and federal worksite cafeterias: barriers, facilitators and keys to success. J Hum Nutr Diet 2016;29:677-86.
13. Kimathi AN, Gregoire MB, Dowling RA, Stone MK. A healthful options food station can improve satisfaction and generate gross profit in a worksite cafeteria. J Am Diet Assoc 2009;109:914-7
