# Supplementary material for: Choice architecture interventions to improve diet and/or dietary behaviour by healthcare staff in high-income countries: a systematic review
Source: BMJ Open. 2019 Jan 23;9(1):e023687. doi: 10.1136/bmjopen-2018-023687 (PMC6347858; doi:10.1136/bmjopen-2018-023687)
Supplement: Author's manuscript [file bmjopen-2018-023687.draft_revisions.pdf]

BMJ Open is committed to open peer review. As part of this commitment we make the peer review history of every article we publish publicly available.

When an article is published we post the peer reviewers' comments and the authors' responses online. We also post the versions of the paper that were used during peer review. These are the versions that the peer review comments apply to.

The versions of the paper that follow are the versions that were submitted during the peer review process. They are not the versions of record or the final published versions. They should not be cited or distributed as the published version of this manuscript.

BMJ Open is an open access journal and the full, final, typeset and author-corrected version of record of the manuscript is available on our site with no access controls, subscription charges or pay-per-view fees (http://bmjopen.bmj.com).

If you have any questions on BMJ Open's open peer review process please email info.bmjopen@bmj.com

BMJ Open

Choice architecture interventions to improve diet and/or dietary behaviour by Healthcare staff in high-income countries: a systematic review

Journal:	*BMJ Open*
Manuscript ID	bmjopen-2018-023687
Article Type:	Research
Date Submitted by the Author:	26-Apr-2018
Complete List of Authors:	Al-Khudairy, Lena; University of Warwick, Population, evidenec and technology<br>Uthman, Olalekan; Warwick Medical School,<br>Walmsley, Rosemary; University of Oxford<br>Johnson, Samantha ; University of Warwick Warwick Medical School<br>Oyebode, Oyinlola; University of Warwick Warwick Medical School, Population, evidence and technology. Division of Health Sciences
Keywords:	choice architecture, diet, health care

SCHOLARONE™
Manuscripts

For peer review only

Title Page

Choice architecture interventions to improve diet and/or dietary behaviour by Healthcare staff in high-income countries: a systematic review.

Authors' names: Lena Al-Khudairy[1], Senior Research Fellow; Olalekan Uthman[1], Assistant Professor; Rosemary Walmsley[1,2], Research Assistant; Samantha Johnson[1], Academic Support Librarian; Oyinlola Oyebode[1], Associate Professor in Public Health.

Address for each author: 1. Warwick Medical School, University of Warwick, Gibbet Hill Campus, Coventry, UK. CV4 7AL.

2. Worcester College, University of Oxford, Oxford, UK. OX1 2HB.

Correspondence to: O.R.O.Oyebode@warwick.ac.uk

For peer review only

Abstract

Objectives: we were commissioned by the behavioural insights team at Public Health England to synthesise the evidence on choice architecture interventions to increase healthier purchasing and/or consumption of food and drink by NHS staff. **Data sources:** we searched electronic databases such as MEDLINE, EMBASE and CINAHL. Reference lists of included studies were screened. Two reviewers independently screened potentially relevant studies. **Design:** a systematic review that included randomised experimental or intervention studies, interrupted time series studies and controlled before and after studies. **Participants:** healthcare staff of high income countries. **Intervention:** choice architecture aimed to improve healthy dietary purchasing and/or consumption **(outcomes)** of staff. We used Hollands et al. (2013) and to expanded to include interventions that altered the price. **Appraisal and synthesis:** eligibility assessment, quality appraisal, data abstraction and analysis were carried out by two reviewers. Quality appraisal of randomised trials was informed by the Cochrane Handbook. Remaining studies were informed by the Risk of Bias Assessment Tool for Nonrandomized Studies. Findings were narratively synthesised. **Results:** we identified 18 studies that met the inclusion criteria of this review. Five studies include multiple workplaces (including health care settings) and 13 were conducted in health care settings only. The intervention in 10 studies was choice architecture only and 8 studies had complex intervention that included a choice architecture element. Interventions involving a proximity element (make behavioural options easier or harder to engage with) appear to be frequently effective at changing behaviour. One study had a sizing intervention and was effective on purchasing. Labelling alone was generally not effective at changing purchasing behaviour. Interventions including an availability element were generally reported to be successful at changing behaviour but none of the included studies examined this element alone. There was no strong evidence for the effect of pricing on purchasing or dietary intake. **Conclusion:** proximity, availability and sizing are choice architecture elements that are likely to be effective for NHS organisations. **Registration number:** CRD42017064872.

Strengths and limitations of the study

- We have carried out several steps to minimise potential bias in the reviewing process: a comprehensive search across major electronic databases relevant to the topic of this review, reference lists of relevant studies were screened and no language restriction was applied. Each included study in this review was comprehensively selected, assessed, data extracted, and quality assessed by two review authors independently.
- We have included studies in which complex interventions and it is not always possible to draw conclusions about the contribution of the choice architecture elements to the outcomes.

Key words choice architecture, diet, health care

Word count 4981

For peer review only - http://bmjopen.bmj.com/site/about/guidelines.xhtml

Introduction

The leading risk behaviour for disability and death in England, as in other high income countries, is poor diet [1]. Worldwide, the proportion of adults who are overweight has increased to 36.9% of men and 38.0% in women [2]. Disease associated with poor diet costs the National Health Service (NHS) at least £5.8 billion per year [3]. Nearly two thirds of adults in England are now overweight or obese [4]. The overall cost of obesity to wider society was projected to reach £27 billion in 2015 [5].

Health care setting have an important role to play in tackling this issue. Firstly, obesity is associated with absenteeism, presenteeism, early retirement, injuries, discrimination, and litigation [6-8]. In England, NHS staff absence due to poor health costs at least £2.4 billion a year [9]. Improving employee health may improve productivity and performance of the workforce leading to improved patient care. Secondly, health professionals play an important role in health promotion. However, a high proportion of healthcare staff do not engage in positive health behaviours themselves [10 ,11]. Nurses with poor health behaviour may be less likely to carry out health promotion activity [12]. A questionnaire survey of 540 pre-registered nurses found that three quarters felt that their physical appearance affected how they were perceived as a nurse [12]. Most respondents felt that patients would be more likely to heed their advice on health behaviours if they appeared to be following that advice themselves. 79.1% agreed that nurses should present themselves as role models for health behaviours [12]. Similarly a questionnaire completed by occupational health staff found that a fifth of them felt their own weight made it difficult to address the issue in patients [13]. By taking action to address diet and obesity for healthcare staff, these organisations and staff will be role models for others. Further, in the UK, the NHS is the largest employer [14]. This means that improving the health and wellbeing of the 1.4 million NHS staff will lift the health and wellbeing levels of the entire UK population.

The WHO defined a healthy workplace as "one in which workers and managers collaborate to use a continual improvement process to protect and promote the health, safety and well-being of all workers and the sustainability of the workplace" [15]. There are initiatives around the world to improve workplace wellbeing and many of these are specific to health care settings [16]. There are a number of policy drivers to encourage and support NHS organisations to improve the food and drink environment including an NHS Commissioning for Quality and Innovation Indicator: Healthy food for NHS staff, visitors and patients [17]. Changing the environment to promote healthy behaviour- such as healthy food and drink purchasing and consumption can be particularly useful and effective because:

- When the environment makes healthy choices easier, this doesn't require any effort by the target audience. This means it isn't just those people who are highly motivated to make healthy changes who benefit.
- Most changes are no-cost or low cost to introduce.

We were commissioned by the behavioural insights team at Public Health England (PHE) to undertake a systematic review and evidence synthesis, examining the literature to inform the evidence base on choice architecture interventions to increase healthier purchasing and/or consumption of food and drink by NHS staff.

For peer review only - http://bmjopen.bmj.com/site/about/guidelines.xhtml

Materials and Methods

The protocol for this systematic review was registered with PROSPERO (an international database of prospectively registered systematic reviews in health and social care) on 1st May 2017 CRD42017064872 [18].

MEDLINE, EMBASE, CINAHL, Cochrane Central register of Controlled Trials, PsycINFO, Applied Social Sciences Index and Abstracts, Web of Science were searched from inception until May 2017. Reference lists of retrieved included studies and of identified relevant systematic reviews were scanned for any additional studies not picked up by our electronic searches. No restrictions were made by language or year of publication. The search terms used included medical subject headings (MeSH) or the equivalent, and text word terms (*i.e.*, health promotion, choice architecture, food services, meals, workplace). A specialist librarian (SJ) was consulted for further search terms. Searches were tailored to individual databases (please see supplementary material 1).

Studies met the inclusion criteria if they fulfilled all of the following:

Study design - randomised experimental or intervention studies, including cluster randomised trials, quasi-randomised studies, interrupted time series studies (before and after) and controlled before and after studies;

Participants - NHS staff or staff from any other health care setting in a high-income country context. We used the World Bank classification for high-income countries, which includes 79 countries in total [19].

Interventions - Behavioural interventions that aimed to improve healthy dietary purchasing and/or consumption of staff were included. We restricted the specific behavioural interventions examined to "choice architecture" or “nudge” or “design” interventions. We used Hollands [20] definition of choice architecture interventions- “those that involve altering small-scale physical and social environments, or micro-environments to cue healthier behaviour” and the associated framework to structure this review. We expanded this framework to include interventions that altered the price of items to cue healthier behaviour (Table 1).

Outcomes - a) dietary (food and/or drink) purchasing (e.g. sales data; receipts analysis) in restaurants, canteens, vending machines or other situations in which food and drinks are sold; or b) dietary intake assessed by validated tools or by more than one dietary measure such as multiple 24-hour dietary recalls. We did not examine caloric intake as self-reported measures showed poor correlation in comparison to objective measures for caloric intake [21].

Table 1: Typology of choice architecture intervention in micro-environments, modified from Hollands et al (2013) [20]

Intervention Class	Intervention Type
Primarily alter properties of objects or stimuli	Ambience- alter aesthetic or atmospheric aspects of the surrounding environment
	Functional Design- design or adapt equipment or function of the environment
	Labelling- apply labelling or endorsement information to product or at point-of-choice
	Presentation- alter sensory qualities or visual design of the product
	Sizing- change size or quantity of the product
	Pricing- change price of the product
Primarily alter placement of objects or stimuli	Availability- add behavioural options within a given micro-environment

For peer review only

For peer review only - http://bmjopen.bmj.com/site/about/guidelines.xhtml

	Proximity- make behavioural options easier or harder to engage with, requiring reduced or increased effort
Alter both properties and placement of objects or stimuli	Priming- place incidental cues in the environment to influence a non-conscious behavioural response
	Prompting- use non-personalised information to promote or raise awareness of a behaviour

Following the database searching, titles, abstracts or both of every record retrieved were screened for potential relevance by two review authors (LA and OO) independently. Studies not carried out in high-income countries, qualitative studies and studies of children were excluded at this stage. Following this preliminary screening, full reports of potentially relevant studies were obtained, and two reviewers (two of LA, OO, OU) independently assessed studies for inclusion/exclusion using a checklist form based on the four inclusion criteria above. Where there was disagreement about the inclusion of a study, a third reviewer was consulted (two of LA or OO or OU). Where resolution of a disagreement was not possible, we added the article to those ’awaiting assessment’ and contacted study authors for clarification. Data were extracted from the included studies by two reviewers (two of LA, OO, OU, RW) independently using a predefined and pre-piloted data abstraction form (please see supplementary material 2). Key data including details on methods, participants, intervention (including mapping to framework domains), outcomes, funding and notable conflicts of interest of study authors. The assessment of the methodological quality of included randomised trials was informed by guidelines from the Cochrane Handbook [22]. Risk of bias for other quantitative study designs used the Risk of Bias Assessment Tool for Nonrandomized Studies [23].

Patient and Public Involvement

As this was a themed commissioned piece of work patient and public advisors were not involved in the development of research question, design of the study or conduct of the study. We were required to develop a toolkit (supplementary material 3) in a companion format designed for the audience who make decisions about catering in NHS organisations. Two advisors (please see acknowledgement) have reviewed the toolkit to ensure that the content relevance to the target audience.

Results

Searching electronic databases generated 17505 hits which equated to 14294 individual records after duplicates were removed. Screening of titles and abstracts for potential relevance excluded 14120 records, as they did not meet the above mentioned inclusion criteria of this review, leaving 174 potentially relevant records. Screening relevant systematic reviews and included studies references yielded an additional 10 records. After formal inclusion/exclusion of 184 records, we identified 28 records from 18 studies met the inclusion criteria of this review. This included nine records from five studies in which the settings were not exclusively hospitals or other health care workplaces, but in which at least one of the sites involved was relevant to our review. In addition we noted ten records from nine studies where we were unable to ascertain whether the study met our inclusion criteria based on information included in the study reports, and author contacts received no replies. In these cases, studies were classified as “awaiting classification” [Figure 1].

Results were narratively synthesised due to the substantial heterogeneity in study design, interventions (even when classified within the same framework category), and outcomes reported. We were unable to proceed with planned meta-analysis or subgroup analyses [18].

For peer review only - http://bmjopen.bmj.com/site/about/guidelines.xhtml

Included studies

Health care settings

Key characteristics of 13 included published studies are presented in Table 2. Eight studies [24-33] examined pure choice architecture interventions and five studies [34-41] examined complex interventions for which some elements were choice architecture. One included study was conducted in the UK [24], three in other European countries [25 ,26 ,31], eight in the USA [27-30 ,32-37 ,39-41] and one in Australia . Eight studies were interrupted time series [25 ,27-34 ,38], two were cluster randomised controlled trials [35 ,36 ,39-41], one was an RCT [37], one was a controlled before and after study [26] and one cross-sectional study [24]. Seven studies examined purchasing outcomes [25 ,27-31 ,38], five examined dietary consumption [24 ,26 ,35-37 ,39-41] and one study examined both purchasing and dietary consumption [34].

Multiple workplaces

Five studies (table 2) in which settings included multiple workplaces and data were not disaggregated for health care settings were included in this review. Two studies [42-45] examined pure choice architecture interventions and three studies [46-49] examined complex interventions for which some element were choice architecture. Two studies were conducted in the USA [46 ,47], two in The Netherlands [42 ,45 ,48] and one in the UK [43 ,44]. Three studies were cluster RCTs [42 ,45-47 ,49], one was a controlled before and after study [48] and one was a cross-sectional study [43 ,44]. Four studies examined dietary consumption [43 ,44 ,46-48] while one examined purchasing [42 ,45]. Further details of included studies are available in table 2.

Awaiting classification

Despite contacting authors we were unable to classify nine studies because sufficient details were lacking in study reports. Five studies were unclear on the setting [50-54]; two studies were unclear on the outcomes [55 ,56]; one study was unclear on the type of participants [57] and one abstract was unclear on several domains [58].

Intervention components and effectiveness

We examined effectiveness of interventions in included studies by whether they were a choice architecture only intervention, or a complex intervention with choice architecture elements. Summaries of the components included in interventions mapped against their reported effectiveness are presented in Tables 3 and 4.

Pure choice architecture

Of the choice architecture only interventions, almost all included studies involved labelling elements, including four studies that examined labelling in isolation. The most frequently studied single element choice architecture interventions were proximity interventions, investigated in five studies [24 ,25 ,27 ,30 ,31]. No included study investigated ambience or priming elements (Table 3). Most of the identified published studies reported successful interventions.

There was mixed evidence for the effectiveness of labelling interventions implemented on their own. Labelling interventions were not found to have an effect on purchasing in two studies [25 ,29], although they were reported to change purchasing behaviour in one other study [28]. In the study that reported successfully changing purchasing through labelling, every item of food on offer in a hospital canteen was labelled with its caloric value. This had a significant effect on the number of calories purchased during the intervention phase. This study was at low risk of bias in three domains,

For peer review only - http://bmjopen.bmj.com/site/about/guidelines.xhtml

however, it is more than four decades old and therefore likely to be examining a slightly different context to present day [28]. There was a low risk of bias in one of the studies that found no effect of labelling [25]. In this study lean (30% less fat) and butter croissants which were similar in price, appearance and taste were on offer in a Dutch hospital restaurant, where they are a popular choice for lunch. During the intervention period, the lean basket was labelled with a sign that stated “The saturated (unhealthy) fat content of this croissant is 30% lower”, however there was no statistical significance between the number or ratio of the two alternative products purchased during the intervention period, compared with the control periods [25]. Another study labelled the two entrees on offer in a hospital canteen (one of which was designed to be healthier) with information on their constituent calories, fat, and sodium but this did not significantly change sales of the healthier option [29]. This study had a high risk of bias from confounding [29]. Overall it appears that labelling is unlikely to be effective on its own.

One study, with a low risk of bias in three domains, examined an intervention which used sizing alone, specifically, offering a smaller size of the main meal of the day, which was successful in terms of changing purchasing behaviour- about 10% of hot meals sold were small meals, with no overall change in total hot meals sold and no compensatory purchasing, for instance purchasing the smaller hot meal and adding a portion of chips) [42 ,45]. A pricing element was also evaluated in this study, in which the smaller meal was either priced proportionately so that both size and price of the meal were 65% of the standard; or ‘value’ pricing was used in which the size of the meal was 65% of the standard, but the price only reduced to 80% of the standard. The pricing condition did not change the effectiveness of the intervention, there was no significant different between the proportionate and value pricing conditions [42 ,45].

Proximity interventions alone were successful at changing purchasing behaviour in two studies [25 ,27]. The interventions included having margarine available in four centrally located and easy-to-reach positions in the hospital restaurant, but butter stored in a fridge, therefore requiring more effort to obtain which reduced sales of butter and increase sales of margarine [25]; re-arrangement of desserts in a hospital canteen so that low-calorie desserts (fruit and jelly) or high-calorie desserts (pies and cakes) were at the front in most accessible positions [27].

Other successful pure choice architecture interventions including more than one element were:

- Labelling + availability interventions which changed dietary consumption in two studies [26 ,43 ,44]. Both these studies involved stipulating the proportion of meals on offer in a canteen that should be healthy (half in Lassen et al, 2014; a third in Holdsworth et al, 2014) and labelling these healthy meals as such. These are the only two pure choice architecture interventions which assessed dietary consumption. However both had a high risk of bias over more than one domain.

- Labelling + proximity + prompting which changed purchasing behaviour in one study with a low risk of bias over most domains [30]. In this study all items on offer were labelled according to a traffic light system, green items were put at eye-level while red items were made less visible, and permanent signage and menu board changes were made to highlight healthy options.

- Availability + proximity intervention which changed purchasing behaviour in one study with a low risk of bias [31]. In this study a set of shelves stocked with snacks was placed on the check-out counter of a hospital canteen. In two phases, the shelves were stacked with 75% healthy and 25% unhealthy snacks and for the other two phases these proportions were reversed. In addition, the shelves were re-arranged so that the healthy snacks were at the top in two phases,

For peer review only - http://bmjopen.bmj.com/site/about/guidelines.xhtml

and at the bottom (requiring stooping to access) in the other two phases. Both availability (proportion of healthy snacks on offer) and proximity (ease of obtaining the healthy snacks) had a positive effect on healthy snack purchasing.

Finally, Geaney et al (2011)[24] used an intervention with several choice architecture elements which was successful in changing dietary consumption. In this study menus were modified to increase availability of healthy options: high-salt products and processed meat were replaced with low-salt options, all desserts were fruit based, cooking methods with oil were limited. In addition salt was removed in all cooking processes and from tables in the staff canteen (although it was available in small sachets from service). Nutritional information on salt reduction and a healthy diet was displayed in the canteen area. No sauces or accompaniments were added to any meals without the customer's consent and extra salad and vegetable options was provided at no extra cost. This study had a low risk of bias and demonstrated an effect on 24 hour dietary recall data [24].

The only unsuccessful multi-component choice architecture only intervention was examined in Dorresteijn et al, (2013) [25] which used labelling + proximity + prompting. In this case, the two soups usually on offer in the restaurant were altered so that one option every day was reduced salt. Signs advertised that this soup "contains 30% less salt and contributes to a healthy blood pressure" however, this did not affect the number or ratio of normal salt to reduced-salt soup cups sold.

Complex interventions including choice architecture

As with the choice architecture only interventions, most of the complex interventions included labelling elements in studied interventions while none included ambience or priming elements. In addition none of the included studies of complex interventions examined presentation or sizing interventions. Where choice architecture interventions were implemented as part of complex interventions (usually involving other elements of behaviour change eg: educational elements) there were many examples of effective interventions [Table 4].

Complex interventions involving an availability choice architecture element were effective at changing dietary consumption in two studies [35 ,46]. In one of these studies, the other elements of the intervention were extensive including pedometers, on-site WeightWatchers® group meetings, an on-site group exercise program, monthly lunchtime seminars, monthly newsletters, walking maps, team competitions, participation cards, and participation rewards and weekly access to a registered dietitian/exercise specialist [35]. The environmental component of the intervention was a weekly healthy snack cart that visited staff [35]. In the other study, the intervention included formation of employee advisory boards in each worksite to implement activities in their workplace including education and information about 5-a-day, and a self-help manual provided to every employee to support them making healthy choices. In addition the "availability" component was providing more fruits and vegetables as part of the regular menus in the worksite canteens [46]. In both cases it is difficult to draw much of a conclusion about the "availability" components compared with the other components of the intervention.

Labelling + prompting was a successful combination when used alongside a complex intervention in one study [38] and labelling + prompting + proximity was effective as part of a complex intervention in Sorensen et al (1992)[47 ,49]. However labelling + proximity, without prompting, was not effective in another study [36 ,39-41] . In all three studies the other (non-choice-architecture) elements of the interventions were direct educational activities- but none were particularly intensive suggesting labelling + prompting might work as pure choice-architecture (labelling + prompting + proximity was also effective in the choice architecture only studies [30].

For peer review only - http://bmjopen.bmj.com/site/about/guidelines.xhtml

Labelling + pricing + availability was effective when used alongside a complex intervention in one study [34]. In this study canteens modified the recipes of some foods offered in order to reduce the energy density and introduced nutritional labels for all foods. In addition some employees were given training in reducing the energy density of their diet and discounts on lower energy dense foods bought in the canteen [34]. This study had high risk of attrition bias and possible evidence of selective outcome reporting. The study reported a significant reduction in total kilocalories purchased in the canteen and in the proportion of energy from fat [34].

Stites et al (2015)[37] used a complex intervention with many choice architecture elements and was effective at changing behaviour. In this intervention the participants received "mindful eating training" and the hospital implemented an online pre-ordering system for staff lunch. The pre-ordering system had nutritional information displayed by all the choices, and for four of the eight week intervention period, the employees received a discount for pre-ordering rather than buying lunch direct in the canteen. Other elements included a pre-ordering system which defaulted to the healthiest choice (requiring additional effort to select less healthy options). This study had low risk of bias and was effective at improving nutritional content of purchased lunches.

In one study, in which the intervention had an effect on dietary consumption, choice architecture elements were chosen and implemented according to what suited the local context with examples relating to availability (which also changed behaviour in two other studies) and prompting (which is not studied in isolation in any other study) [59]

Methodological Quality of Included Studies

The risk of bias of included studies is presented in Tables 5 and 6. High risk of bias was seen in two of the six RCTs. Racette et al, (2009) [35] although using randomisation, only included two worksites, with quite different employee characteristics which may have introduced other sources of bias. Sorensen et al, 1999 [36 ,39-41] collected more dietary data than was presented in the report, without clear justification for why specific outcomes were reported. Nearly all of the included randomised studies did not provide details on the process of randomisation (selection bias). Two studies [37 ,42 ,45] were at low risk of performance bias where blinding was not possible but unlikely to affect objective outcomes.

In the non-randomised studies, high risk of bias in more than one domain was seen in four [26 ,34 ,38 ,43 ,44] of the twelve included studies. One study [29] had errors in the numerical data presented which was cause for concern (number of entrees sold and mean number of entrees sold/day). Only two studies were judged at low risk of bias across all domains [25 ,31]. Half of the studies were at low risk of bias for confounding variables.

For peer review only - http://bmjopen.bmj.com/site/about/guidelines.xhtml

Discussion

Statement of principal findings

Choice architecture interventions involving a proximity element appear to be frequently effective at changing behaviour. This was found true in some studies that used proximity interventions alone or as part of choice architecture or complex interventions. Interventions including an availability element were generally reported to be successful at changing behaviour. However, none of the studies examined availability alone. A sizing intervention was effective in the only study identified examining this type of intervention.

There was no strong evidence for the effect of pricing on behavioural change in our review. No additional effect of pricing in a sizing and pricing intervention compared with sizing alone, and one complex intervention including pricing which was reported to be effective, but had a high risk of bias. Labelling alone was generally not effective at changing behaviour, although it was a popular addition to other interventions of which most were successful.

Strengths and weaknesses of the study

Because of the relatively small number of papers that we found during a scoping search, we made the decision to broaden the inclusion criteria in two ways. Firstly, we have included studies in which complex interventions with non-choice architecture elements were implemented and it is not always possible to draw conclusions about the contribution of the choice architecture elements to the outcomes. Secondly, we have included studies in which not all the workplaces were healthcare settings. We did this because this gives us some evidence of interventions which are feasible in healthcare settings, however because data was not disaggregated, we cannot be sure that outcomes were similar across the different sorts of workplaces included (ie: an element that was effective overall, may have been less effective or not effective in the health-care settings; or an element that was not effective overall may have been effective in the health-care settings). We conducted a comprehensive search across major electronic databases relevant to the topic of this review. Each included study in this review was comprehensively selected, assessed, data extracted, and quality assessed by two review authors independently to minimise potential biases in the review process. Where data of relevance were missing, either to allow assessment of eligibility, or at the data extraction stage and assessment of bias, the review authors (OO, LA, OU) contacted the study authors for further information.

Strengths and weaknesses in relation to other studies, discussing important differences in results

When Hollands et al (2013) [20] appraised the choice architecture literature in mid-2011, for the purposes of developing the typology we have used in this review, they noted that 40% of all study reports examined interventions which involved point-of-choice labelling and/or prompting elements [20]. We found labelling and proximity elements were the most popular type of choice architecture employed in our included studies, both alone or in combination with other intervention elements. The difference might be due to the scope of the literature examined, we were restricted to those aiming to change dietary behaviour while Hollands et al (2013) looked more broadly at a range of behaviours.

The finding of this review found mixed results for labelling interventions suggesting that they are unlikely to be effective in isolation. A recent systematic review evaluating the effect of restaurant menu calorie labelling found 19 studies. Meta-analysis of these studies demonstrated an 18 calorie reduction in meals ordered when all studies were combined. However, when just the six controlled studies were examined, labelling was no longer associated with a significant difference in calories ordered [60]. Another systematic review found that menu labelling with calorie information alone

For peer review only - http://bmjopen.bmj.com/site/about/guidelines.xhtml

did not reduce the number of calories selected or consumed, although the addition of information to assist consumers in applying nutritional information was effective [61]. In a systematic review of randomised controlled trials that included food labels and excluded menu labelling, nine studies were identified and meta-analysis concluded that food labelling was effective at changing dietary behaviour although not at reducing calorie consumption [62]. Menu energy labelling across socio-economic groups was evaluated in a recent review [63]. The findings suggested that any positive benefit of this kind of labelling may only apply to higher socio-economic groups [63]. The findings of this review in the context of previous literature suggests that labelling alone should not be recommended for implementation to improve healthy food and drink purchasing of health care staff .

A large systematic review evaluated supermarket and grocery store interventions to increase consumption of healthy. Analysis of forty two studies found that consumers responded to economic incentives including discounts, vouchers and subsidies [64]. Similarly, a systematic review of interventions at point-of-sale to encourage healthier food purchasing found evidence for monetary incentives as effective at changing behaviour, but that there was no strong evidence for the other intervention types they examined [65]. We found mixed evidence on pricing in health care settings in this review.

A systematic review of interventions carried out in restaurant settings to promote healthy eating stated that the most effective interventions were point-of-purchase information with increased availability of healthy choices [66]. We also found that increasing availability of healthy options alongside other intervention elements was a successful strategy for driving behaviour change in health care settings.

Meaning of the study: possible explanations and implications for clinicians and policymakers

On the basis of this systematic review, we were able to recommend some general principles that are likely to be effective when designing choice architecture interventions for NHS organisations to Public Health England. These are “proximity” interventions in which behavioural options are made easier or harder to engage with, requiring reduced or increased effort and “availability” interventions in which healthy behavioural options are added within a given micro-environment. In addition, we are able to suggest “sizing” interventions in which the size or quantity of the product on offer is changed based on one effective study with low risk of bias. Finally, we can recommend specific examples of choice architecture interventions that have been effective in studies with low risk of bias. These recommendations are made in a companion format (please see supplementary material 3), designed for the audience who make decisions about catering in NHS organisations.

Other outcomes relevant to decision-makers

We examined primary outcomes relating to dietary consumption and purchasing of food and drink. However, outcomes that are important to decision-makers include customer satisfaction and profitability. One of our included studies which examined various arrangements and assortments of snacks on offer at the checkout counter in a hospital staff restaurant found in a survey of 92 participants that the more visible the healthy snacks were in terms of location (ie: on top shelves) and proportion (ie: 75% healthy vs 25% healthy) the more attractive the entire selection was perceived to be [31]. Another included study noted that the presentation of caloric values of the food significantly decreased the total number of calories bought without significantly affecting the total money spent [28].

In a study of nine early adopters of the Hospital Healthier Food Initiative and the Health and Sustainability Guidelines in the USA, the main barriers to implementation were reported to be

For peer review only - http://bmjopen.bmj.com/site/about/guidelines.xhtml

customer complaints, a shortage of food and drinks that met the requirements of the Initiative/Guidelines and concern over profitability. However, most respondents had been able to overcome these barriers and it was noted that customer volumes and sales were reported to have increased subsequent to implementation which offset initial investments necessary to successfully meet the requirements of the Initiative/Guidelines [16].

A study in which a "healthful food station" was introduced to a worksite cafeteria in an academic medical centre in the USA, found that some customers reported that they used the cafeteria more often due to the presence of the station. Others had only started using the cafeteria because the station had been introduced. This study also found that the healthful food station generated gross profit [56].

Unanswered questions and future research

Reporting of studies was generally poor. For instance both randomised and non-randomised studies did not generally report on domains that may lead to selection bias. We are generally unsure whether subjective outcomes were collected by blinded assessors which may lead to detection bias. Overall, the methodological quality of included studies was low and future research should clearly report on this. Therefore the results of this review should be interpreted with cautious.

In health care settings, increasing availability of healthy options alongside other intervention elements was found to be successful in improving behaviour. However, we do not know the effect of increasing availability alone.

We could not come to a clear conclusion whether a pricing element is clearly successful in improving healthy food and drink purchasing and consumption in health care settings.

No studies investigated the effect of ambience or priming elements on modifying dietary behaviour in health care settings.

Declaration of competing interests

All authors have completed the ICMJE uniform disclosure form at www.icmje.org/coi_disclosure.pdf and declare: no support from any organisation for the submitted work; no financial relationships with any organisations that might have an interest in the submitted work in the previous three years; no other relationships or activities that could appear to have influenced the submitted work.

Details of contributors

LA and OO designed the protocol; LA, OO and SJ designed and ran the searches; LA and OO performed the initial screening; LA, OO and OU screened full text articles; LA, OO, OU and RW extracted data from included studies; LA and OO synthesized extracted data and wrote the first draft of the manuscript. All authors contributed to the final submitted manuscript.

Acknowledgement

The authors would like to thank Aileen Clarke, Warwick Medical School; Ruth Breese, South Warwickshire NHS Foundation Trust; Elizabeth Atherton, Food for Life Programme, Soil Association; Laura Brown, Sarah Golding and Tim Chadborn, Public Health England, all of whom gave comments and feedback that have supported this work.

Ethics approval

Not required

For peer review only - http://bmjopen.bmj.com/site/about/guidelines.xhtml

Funding Sources

This piece of research was funded by Public Health England Behavioural Insights Team who gave feedback on the study protocol. The funder had no role in the collection, analysis or interpretation of data, in the writing of the report or decision to submit the article for publication. LA and OO are supported by the National Institute of Health Research (NIHR) Collaboration for Applied Health Research and Care West Midlands initiative. The views expressed are those of the authors and not necessarily those of the NHS, the NIHR or the Department of Health. RW was supported by University of Warwick through its Global Research Priority on Food. OU is supported by the National Institute of Health Research using Official Development Assistance (ODA) funding. SJ is supported by the University of Warwick.

Transparency Declaration

The lead author affirms (as the manuscript's guarantor) that the manuscript is an honest, accurate, and transparent account of the study being reported; that no aspects of the study have been omitted; and that any discrepancies from the study as registered have been explained.

Data sharing statement

No additional data available.

What this paper adds

What is already known on this subject

- Changing the environment to promote healthy behaviour can be a low cost effective strategy.
- Proximity and prompting are choice architecture elements that are commonly used in work place settings to improve behavior of employees.

What this study adds

- Proximity, availability and sizing are elements that are likely to be effective when designing choice architecture interventions for NHS organisations.
- Labelling is a popular addition to other interventions in choice architectural literature. Labelling alone is not recommended for implementation to improve healthy food and drink purchasing of health care staff.

Figure 1: PRISMA flow diagram

For peer review only - http://bmjopen.bmj.com/site/about/guidelines.xhtml

Table 2: Characteristics of included studies

Study	Participants/worksite	Interventions	Outcomes
**Dorresteijn (2013)**[67]	Canteen, Medical Centre.<br>University Medical Centre with over 11 000 employees, over 1000 hospital beds and over 2000 customers visiting the hospital restaurant each day.<br>***Inclusion/exclusion:*** N/A (data not collected from individuals).	(1) Point-of-decision prompts on hospital elevator doors promoting stair use. In the hospital restaurant: (2) Point of- purchase prompts promoting reduced-salt soup.<br>(3) Point-of-purchase prompts promoting lean croissants.<br>(4) Reversal of the accessibility and availability of diet margarine and butter.<br>***Comparator:*** No comparator	Number and ratio of purchased normal-salt soup, reduced-salt soup, croissants, lean croissants purchased, diet margarine and butter.
**Geaney (2011)** [68]	Canteen, hospital.<br>Participants were representative sample of 100 adults aged 18–64 years working in two public sector hospitals (fifty staff from each hospital).<br>***Inclusion:*** Being an employee and consuming at least one main meal in the hospital staff canteen daily.<br>***Exclusion:*** None reported.	Restricting food high in salt, fat and sugar. Modifying menus to make healthier options available. Replacing purchasing orders for high salt products (e.g. bacon) with low salt alternatives (e.g. turkey). Salt removed in all cooking places and tables but available in small sachets at service. Nutrition information on salt reduction and a healthy diet displayed. No sauces or accompaniments added without customer's consent. Staff encouraged to consume extra salad and vegetable options at no extra charge. Cooking oil use limited. All desserts fruit based.<br>*Comparator:* canteen at another hospital with no intervention.	Macronutrients (g/d): total sugars, sucrose, fructose, total fat, saturated fat, protein, carbohydrates, salt.<br>Micronutrients (mg/d): K, Ca, Fe, Vitamin B6, Vitamin B12, Vitamin C, Vitamin D.
**Lassen (2014)**[69]	Canteen, hospital.<br>The canteen was seeking Keyhole certification in Denmark. Participants were canteen customers approached at random after purchasing lunch.<br>***Inclusion:*** Purchasing lunch in the	Introduction of the Keyhole symbol on freshly prepared food in the canteen. The Keyhole symbol is used in the Nordic countries as a sign of a healthy choice (meeting certain criteria).<br>***Comparator:*** no intervention.	Energy per meal consumed (kJ), energy density of meal (kJ/100g), fat content (E%), fruit and vegetables (g/100g), salt (g/100g), , refined sugars (g/100g), wholegrain (g/100g)

For peer review only - http://bmjopen.bmj.com/site/about/guidelines.xhtml

	canteen.<br>***Exclusion:*** Not reported.		
**Lowe (2010)** [34]	Canteens, hospitals.<br>***Inclusion:*** Reporting eating lunch in the hospital cafeteria at least two times each week, on average, being between 21 and 65 years of age and being a hospital or university employee.<br>***Exclusion:*** Having a current diagnosis of a chronic disease or condition known to affect appetite or body weight, taking medication known to affect appetite or body weight, being pregnant or planning to become pregnant within the next 24 months, being enrolled or having plans to 15 label within the next 24 months in an organized weight management program, and/or having plans to terminate hospital employment within the next 12 months.	When the intervention period began, participants in both groups were exposed to environmental change (EC): reductions in the energy density (ED) of some foods offered in the cafeteria and introduction of nutritional labels for all foods sold in the cafeterias. Participants in the EC-Plus condition received two additional intervention components: training in reducing the ED of their diet and discounts on low-ED foods purchased in the cafeteria.<br>***Comparator:*** environmental change only.	Fruit (servings/ day) , vegetables (servings/day) , bread products (servings/day) , dairy products (servings/day) , fats and sweets (servings/day) , meats (servings/day); Purchased kcal (kcal): purchased calories from fat (%); purchased calories from protein (%); purchased calories from carbohydrate (%)
**MacDonald (2016)** [70]	Aboriginal Community controlled health organisations.<br>**Inclusion/exclusion:** not reported.	A tailored 'Healthy Catering Toolkits' to local caterers at each site and included order forms classified according to the 'traffic light guide' to ensure healthy catering choices were easy ordering choices for staff. They also distributed 'traffic light guide' posters and information sheets, a nutrition policy template and a wallet-sized card for interpreting food labels.<br>**Comparator:** no comparator.	Proportion of purchased foods in category 'foods to limit' (%)
**Meyers (1980)** [71]	Canteen, Hospital.<br>A hospital cafeteria that served hospital staff, students and hospital visitors.<br>**Inclusion/exclusion:** not reported.	Desserts were arrayed in columns four deep along the cafeteria counter. On control days research assistants arrayed desserts in columns of four, high calorie	Dessert purchases.

For peer review only - http://bmjopen.bmj.com/site/about/guidelines.xhtml

		desserts alternating with low-calorie desserts, permitting equal access to each dessert. High-calorie desserts were placed in the two front positions with low calorie desserts in the read (less accessible) positions on days 3 and 5. On days 2 and 4 this order was reversed.<br>**Comparator:** No comparator.	
**Milich (1976)** [28]	Canteen, hospital.<br>**Inclusion:** Being a female employee (identifiable by an ID badge and/or uniform) and having a food tray.<br>**Exclusion:** Being a patient or visitor, being visibly pregnant or being an employee who had brought food from home or only bought part of her meal in the cafeteria.	Calorie signs were posted for foods in the cafeteria.<br>The intervention consisted of 1 week of Price Increase, followed by 1 week of Price Increase and Calorie Presentation<br>**Comparator:** No comparator.	Calories bought (kcal), money spent ($).
**Racette (2009)** [35]	Worksites, medical centre.<br>**Inclusion:** All employees over 18 years of age were eligible, including those who smoked, had pre-existing disease (e.g. hypertension, diabetes), or used medications.<br>**Exclusion:** not reported.	The intervention was comprised of a constellation of nutrition components, physical activity components, and incentives designed to promote healthy dietary and physical activity behaviours, with the goals of promoting weight control and reducing risk factors for cardiovascular disease.<br>Specific intervention components included weekly healthy snack cart, monthly lunchtime seminars, monthly newsletters, walking maps, participation cards and participation rewards. Each week a registered dietitian/exercise specialist was available at the worksite to provide support.<br>**Comparator:** Health assessments only.	Fruit and vegetable intake (servings/day).

For peer review only - http://bmjopen.bmj.com/site/about/guidelines.xhtml

**Sato (2013)** [29]	Cafeteria, Medical Centre.<br>Customers of the medical centre cafeteria aged 18 years and older. Roughly 70% of the customers at Kaiser Permanente San Francisco Medical Center are Kaiser employees; the rest are visitors or patients in the hospital. The cafeteria serves roughly 100 customers per day.<br>**Inclusion/exclusion:** N/A (receipts collected anonymously).	A 'Healthy Pick' meeting various criteria was made available every day. The meals available were labelled with a label featuring calories, fat and sodium.<br>**Comparator:** No comparator.	Number of Healthy Picks purchased, number of main meals purchased.
**Sorensen (1999)** [36 ,39-41]	Community health centres.<br>**Inclusion:** Being a permanent employee and working at least 15 hours a week.<br>**Exclusion:** not reported.	The core intervention included periodic exposure to national 5-a-Day campaigns and a general nutrition presentation. In the additional worksite intervention, educational activities and changes were made to the worksite environment including labelling and adding fruit and vegetables to vending machines.<br>**Comparator:** core intervention only.	Fruit and vegetable intake (servings/ day).
**Stites (2015)** [37]	Canteen, hospital.<br>**Inclusion:** Having a BMI of at least 25.0 kg/m^2, eating at least three lunches/week in the hospital cafeteria, being willing to allow researchers to collect data about their lunch purchases, and having access to a computer at work.<br>**Exclusion:** Having a diagnosis of unstable hypertension, dyslipidemia, or coronary artery disease whose medical therapy had changed in the past three months, having plans to terminate hospital employment within four months following study enrolment, or being pregnant.	During the Full Intervention phase participants received mindful eating training, were encouraged to pre-order their lunches, and were given vouchers to use on lunch purchases. Following this there was a Partial Intervention phase, where participants were encouraged to pre-order their lunches but did not receive vouchers. The pre-ordering system listed the food available daily in the cafeteria, along with nutritional information. Employees had to order at least 45 minutes ahead of scheduled pick up time.<br>**Comparator:** wait list control.	Kcal per lunch purchased (kcal),<br>fat grams per lunch purchased (g).

For peer review only - http://bmjopen.bmj.com/site/about/guidelines.xhtml

**Thorndike (2014)** [30 ,32 ,33]	Canteen, hospital.<br>Hospital employees have the option of paying for cafeteria purchases by direct payroll deduction using a "platinum plate" card.<br>**Inclusion:** Using "platinum plate" and making a purchase in the cafeteria at least three times during each 3-month period from baseline to the end of follow up.<br>**Exclusion:** Not reported.	A traffic light food labelling system was introduced, along with signage explaining it. Three months later further changes were introduced, including rearranging items in the beverage and sandwich refrigerators to put all the green items at eye level; placing baskets of bottled water throughout the cafeteria; and providing pre-packaged salads next to the pizza counter.<br>**Comparator:** No comparator.	Of items bought: % red, % green (red labels and green labels as denoting unhealthy and healthy choices respectively).<br>Of beverages bought: % red , % green.
**Van Kleef (2012)** [31]	Canteen, Hospital.<br>**Inclusion/exclusion:** N/A	Four successive weeks were randomly assigned to four experimental conditions:<br>Intervention 1: 25% of products healthy, these located at top shelves.<br>Intervention 2: 25% of products healthy, these located at bottom shelves.<br>Intervention 3: 75% of products healthy, these located at bottom shelves.<br>Intervention 4: 75% of products healthy, these located top at shelves.<br>**Comparator:** no comparator.	Number of healthy snacks sold, number of unhealthy snacks sold.
**Beresford (2001)** [72]	28 worksites (6 health service organisations).<br>Inclusion: (For worksites) Having a food-serving cafeteria and having between 250 and 2000 employees.<br>**Exclusion:** N/A	The intervention was tailored to worksites. Elements included a kick-off event, the 5-a-Day message being posted on boards in each worksite, more fruit and vegetables becoming part of the menus and the provision of a self-help manual for every employee.<br>**Comparator:** no intervention.	Fruit and vegetable intake (servings/ day).
**Holdsworth (2004)** [43 ,44]	6 worksites (two health care).<br>**Inclusion/exclusion:** not reported.	The Heartbeat Award scheme increases opportunities for behaviour change by providing customers with information,	Daily intake of: biscuits and cakes, sweet puddings, confectionery, sugary drinks, processed meat, hard cheese,

For peer review only - http://bmjopen.bmj.com/site/about/guidelines.xhtml

		reminders and reinforcement to guide them towards healthier choices. **Comparator:** no intervention.	crisps, low-fat cheese, fried food, beans and pulses, fruit, vegetables, chicken and fish, starchy foods, red meat, low calorie drinks. All reported: (1) no change/negative change; (2) positive change.
**Kwak (2009)** [73 ,74]	12 worksites (two hospitals). **Inclusion:** Employees with BMI > 18kg/m2 and not under any medical restrictions. **Exclusion:** Not reported.	There was an individual and an environmental component to the intervention. Interventions included changes in the assortment of food products in the cafeteria, workshops, an information wall containing information on the balance between food intake and physical activity, posters or prompts stimulating stair use, and ways to form lunch-walking and cycling groups.	Intake of: Fibre rich bread, low fibre bread, fibre-rich main meal, low energy dense toppings, high energy dense toppings, low energy dense main meal, high energy dense main meal, low energy dense snacks, high energy dense snacks, low energy dense drinks, high energy dense drinks (all in servings/day).
**Sorensen (1992)** [47 ,49]	16 worksites (at least one intervention and one control site specialising in "health care"). **Inclusion:** (for worksites) Having 200-2000 employees, having a cafeteria with a kitchen, having an annual turnover rate of <25%, having <25% of employees working rotating shifts, part time or off site and being stable as a company, defined as having no plans for geographic relocation or major layoffs in the next 2 years. **Exclusion:** not reported.	Worksites received a 15-month intervention with various elements. These included direct education programs, such as classes offered in all intervention worksites and optional activities tailored to individual worksites, and environmental programs which targeted cafeterias both to increase the availability of low-fat, high-fibre foods and to provide supportive nutrition education by labelling those food options. **Comparator:** no intervention.	Total dietary fat (% kcal), total dietary fibre (g)
**Vermeer (2011)** [42 ,45]	25 worksites (15 hospitals). **Inclusion:** (for worksites) Selling on average 30 meals/day, offering a reducible meal at least twice per week, being willing to be randomly allocated to a study condition, following the	Intervention 1: a smaller portion (that is, about two-thirds of the size of the existing portion) was offered in addition to the existing portion and proportional pricing was employed (that is, the price was 65% of the existing size).	Number of large meals sold, number of small meals sold, number of fried snacks sold.

For peer review only - http://bmjopen.bmj.com/site/about/guidelines.xhtml

	study protocol and providing their daily sales figures of hot meals and fried snacks.<br>**Exclusion:** not reported.	Intervention 2: a smaller portion was added to the assortment and value size pricing (that is, a lower price per unit for large portions than for small portions) was employed (that is, the price was 80% of the existing size).<br>**Comparator:** no intervention.	

N/A: not applicable

For peer review only

Table 3: Choice architecture elements and effectiveness of interventions examined in included studies

Intervention Class	Intervention Type	Studies												
		Dorresteijn 2013			Geaney 2011	Holdsworth 2004	Lassen 2014	Meyers 1980	Milich 1976	Sato 2013	Thorndike 2014	Van Kleef 2012	Vermeer 2011	
		a	b	c									a	b
Primarily alter properties of objects or stimuli	Ambience													
	Functional design				X									
	Labelling	X	X		X	X	X		X	X	X			
	Presentation				X									
	Sizing												X	X
	Pricing													X
Primarily alter placement of objects or stimuli	Availability	X				X	X					X		
	Proximity			X	X			X			X	X		
Alter both properties and placement of objects and stimuli	Priming													
	Prompting	X									X			
Outcome (N= not effective; Y= effective)	Effect on purchasing?	N	N	Y				Y	Y	N	Y	Y	Y	Y*
Outcome (N=not effective; Y= effective)	Effect on dietary consumption?				Y	Y	Y							

X blue box: element was implemented in the study; *although sizing plus pricing had an effect compared with control, there was no significant effect between sizing and pricing and sizing alone in this study.

For peer review only

Table 4: Choice architecture elements included in complex interventions and their reported effectiveness in included studies

		Studies							
**Intervention Class**	**Intervention Type**	**Berefsord 2001**	**Kwak 2009**	**Lowe 2010**	**Macdonald 2016**	**Racette 2009**	**Sorensen 1992**	**Sorensen 1999**	**Stites 2015**
**Primarily alter properties of objects or stimuli**	**Ambience**								
	**Functional design**								X
	**Labelling**			X	X		X	X	X
	**Presentation**								
	**Sizing**								
	**Pricing**			X					
**Primarily alter placement of objects or stimuli**	**Availability**	X	X*	X		X	X		
	**Proximity**							X	X
**Alter both properties and placement of objects and stimuli**	**Priming**								
	**Prompting**		X*		X		X		
**Outcome** (N= not effective; Y= effective)	**Effect on purchasing?**				Y				Y
	**Effect on dietary consumption?**	Y	Y	Y		Y	Y	N	

X blue box: element was implemented in the study; *****Kwak et al, (2009) used an assets-based approach in which intervention worksites chose intervention elements that suited their context. Examples of actions given were classified as "availability" and "prompting".

For peer review only

For peer review only - http://bmjopen.bmj.com/site/about/guidelines.xhtml

Table 5: Risk of bias in randomised controlled trials

	Selection bias		Performance bias	Detection bias	Attrition bias		
**Study**	random sequence generation	allocation concealment	blinding of participants + personnel (subjective/ objective outcomes)	blinding of outcome assessment (subjective/ objective outcomes)	incomplete outcome data (subjective/ objective outcomes)	**Selective outcome reporting**	**Other bias**
**Beresford 2001**	No details	No details	Blinding not possible	No details. Lack of blinding may affect subjective outcomes	Numbers provided but reasons not fully explained	Outcomes reported as in method. No protocol.	No other bias
**Racette 2009**	No details	No details	Blinding not possible	No details. Lack of blinding may affect subjective outcomes	Numbers and reasons provided	No protocol	Randomisation was only over two sites. Demographical difference between groups.
**Sorensen 1992**	No details	No details	Blinding not possible	No details	Numbers provided and gives some reason for questionnaires being eliminated from analyses	FFQ was used but only fibre and fat reported.	No other bias
**Sorensen 1999**	No details	No details	Blinding not possible	No details	Not reported but analysis presented for all sample (not restricted for completers).	"*Additional dietary data, not presented here, were collected by means of the Food Frequency Questionnair*"	No significant differences among the groups at baseline. All analyses were computed by taking into consideration the nesting of employees in worksites.
**Stites 2015**	Using a computer program	No details	Blinding not possible	No details	Mainly reported and reasons explained,	Outcomes reported as in	No other bias

For peer review only - http://bmjopen.bmj.com/site/about/guidelines.xhtml

					for one participant, no reason explained.	method. No protocol.	
**Vermeer 2011**	No details	No details	Blinding not possible	No details	Numbers provided but reasons not fully explained	Outcomes reported as in method. No protocol.	No other bias

Orange: unclear risk of bias; Green: low risk of bias; Red: high risk of bias; "*quote form publication*"

For peer review only

For peer review only - http://bmjopen.bmj.com/site/about/guidelines.xhtml

Table 6: Risk of bias is non-randomised studies

	Selection bias		Performance bias	Detection bias	Attrition bias	
**Study**	selection of participants	confounding variables	Measurement of exposure	blinding of outcome assessment (subjective/ objective outcomes)	incomplete outcome data (subjective/ objective outcomes)	**Selective outcome reporting**
**Dorresteijn 2013**	Data were collected prospectively	Confounding variables (weather) were considered	All were exposed, performance bias is not affected by exposure	Blinding not possible and unlikely to affect objective outcomes	Characteristics of study before and after the intervention are available	All of the expected outcomes were included in the study descriptions
**Geaney 2011**	*"A random sample of 100 individuals took part in the study (fifty staff from each hospital)." "In the intervention hospital, all individuals who were asked to participate agreed. Less than five in the non-intervention hospital refused to participate in the study."*	Confounding variables (age and gender) were considered	All were exposed, performance bias is not affected by exposure	No details	Cross-sectional there was no attrition	All of the expected outcomes were included in the study descriptions
**Holdsworth 2004**	*" Two premises applying for the HBA were*	Confounding variables (adjusted for age, gender,	All were exposed, performance bias is not affected by	Lack of blinding may affect subjective outcomes	Numbers provided (low response rate)	All of the expected outcomes were included in the study

For peer review only - http://bmjopen.bmj.com/site/about/guidelines.xhtml

	unsuccessful in receiving it, and were used as a comparison group for the study"	ethnicity, social class and BMI) were considered	exposure			descriptions
**Kwak 2009**	*"A sample of 128 worksites meeting the inclusion criteria were selected through the Chamber of Commerce and invited by letter and telephone to take part in this study….12 worksites agreed to participate"*	Confounding variables (matched based on the social economic status of their employees) were considered	All were exposed, performance bias is not affected by exposure	Lack of blinding may affect subjective outcomes	*" At baseline, the average response rate to the questionnaire was 88.1% (487 of 553). At the first follow-up, the response rate average was 82.5% (n=376) and at the second follow-up 75.8% (n=303). During the course of the project, one of the worksites in the intervention group underwent reorganization; half of the employees were made redundant, thereby causing most of the dropout in our study. There was no selective dropout at the first follow-up. At the second follow-up, smokers were more likely to discontinue the study than non-smokers"*	More items collected than reported
**Lassen 2014**	*"The employees*	Confounding	All were exposed,	Lack of blinding	Evidence significant	*"For the outcomes of*

	were randomly approached after having bought their meal and asked to participate in the study focusing on canteen food intake".	variables (demographics, seasonal change) were not considered	performance bias is not affected by exposure	unlikely to affect objective outcomes.	differential in number of health care personnel between baseline & follow-up	*refined sugar, wholegrain and plate waste a large number of participants had zero intake. These were analysed in two steps. First the probability of any intake yes/no was analysed…. then a regression model was fitted as described above with regard to the participants with a positive intake"*
**Lowe 2010**	*"Participants were recruited through letters distributed to cafeteria patrons that provided an overview of the study along with inclusion and exclusion criteria"*	No details	All were exposed, performance bias is not affected by exposure	Lack of blinding is unlikely to affect objective outcomes.<br>For subjective outcomes *"Dietary recalls were conducted by the Diet Assessment Center at The Pennsylvania State University"*	Numbers provided (high attrition rate). *"Data from participants who did not scan their cards an average of at least four times per month were excluded from analyses"*	Reporting of data was not always per intervention group with no justification
**Macdonald 2016**	5 centres were chosen to participate including a range of contexts (metropolitan, rural, regional)	Confounding variables (changes at the sites) were not considered	All were exposed, performance bias is not affected by exposure	*"catering receipts received included generic food types such as sandwiches. Categorisation into food types in these instances were*	*"two ACCHOs were able to provide catering receipts at baseline and follow up"*	All of the expected outcomes were included in the study descriptions

For peer review only - http://bmjopen.bmj.com/site/about/guidelines.xhtml

				based on inferences of the project dietitian"<br><br>*"the number of serves provided where invoices listed general categories; for example, 'large fruit platter' were made from inferences based on cost by the project dietitian"*		
**Meyers 1980**	Data were collected prospectively	No details	All were exposed, performance bias is not affected by exposure	*"observations were carried out by undergraduate research assistants"*	N/A	All of the expected outcomes were included in the study descriptions
**Milich 1976**	*"From original total pool of sampled employees 50 subjects were randomly sampled for each body size in each experimental condition"*	Confounding variables (weather, obesity and price change) were considered	All were exposed, performance bias is not affected by exposure	Outcome assessed by assessing food items on plate then calculating caloric value	N/A	All of the expected outcomes were included in the study descriptions
**Sato 2013**	Data were prospectively collected	Confounding variables (price change) were not considered	All were exposed, performance bias is not affected by exposure	Lack of blinding is unlikely to affect objective outcomes.	N/A	All of the expected outcomes were included in the study descriptions
**Thorndike 2014**	*"employee platinum plate users who made a*	Confounding variables (age, gender, race, job	All were exposed, performance bias is not affected by	Lack of blinding is unlikely to affect objective outcomes.	*"1.7% of employees missing any sociodemographic data*	All of the expected outcomes were included in the study

For peer review only - http://bmjopen.bmj.com/site/about/guidelines.xhtml

	purchase in the cafeteria at least three times during each 3-month period from baseline to the end of follow up were included in the study cohort”	type, and work status) were considered	exposure		*were excluded from the analysis”* Information about the number of participants before and after the study exists, however at each time point the cohort was not identical	descriptions
**Van Kleef 2012**	*“carried out in hospital staff restaurant and visitors are allowed to purchase displayed snacks at a self-service checkout counter throughout the day over a four week period. Data were collected prospectively”*	No details	All were exposed, performance bias is not affected by exposure	Lack of blinding is unlikely to affect objective outcomes.	N/A	All of the expected outcomes were included in the study descriptions

Orange: unclear risk of bias; Green: low risk of bias; Red: high risk of bias; Grey: not applicable; N/A: not applicable; “*quote form publication*”

For peer review only - http://bmjopen.bmj.com/site/about/guidelines.xhtml

References

1. Newton JN, Briggs AD, Murray CJ, et al. Changes in health in England, with analysis by English regions and areas of deprivation, 1990–2013: a systematic analysis for the Global Burden of Disease Study 2013. The Lancet 2015;386:2257-74.
2. Ng M, Fleming T, Robinson M, et al. Global, regional, and national prevalence of overweight and obesity in children and adults during 1980–2013: a systematic analysis for the Global Burden of Disease Study 2013. The Lancet 2014;384:766-81.
3. Scarborough P, Bhatnagar P, Wickramasinghe KK, et al. The economic burden of ill health due to diet, physical inactivity, smoking, alcohol and obesity in the UK: an update to 2006–07 NHS costs. J Public Health 2011;33:527-35.
4. NHS Digital. Secondary Health Survey for England 2015: Adult overweight and obesity 2016. https://digital.nhs.uk/catalogue/PUB20562 (accessed November 2017).
5. Butland B, Jebb S, Kopelman P, et al. Tackling obesities: future choices-project report. London: Department of Innovation, Universities and Skills 2007; 10:17.
6. Shrestha N, Pedisic Z, Neil-Sztramko S, Kukkonen-Harjula KT, Hermans V. The impact of obesity in the workplace: a review of contributing factors, consequences and potential solutions. Current obesity reports 2016;5:344-60.
7. Koepp GA, Snedden BJ, Levine JA. Workplace slip, trip and fall injuries and obesity. Ergonomics 2015;58:674-79.
8. Flint SW, Snook J. Disability discrimination and obesity: the big questions?. Current obesity reports 2015;4:504-09
9. NHS England 2015. Simon Stevens announces major drive to improve health in NHS workplace. https://www.england.nhs.uk/2015/09/nhs-workplace/ (accessed November 2017).
10. Blake H, Mo PK, Lee S, Batt ME. Health in the NHS: lifestyle behaviours of hospital employees. Perspectives in public health 2012;132:213.
11. Kyle RG, Neall RA, Atherton IM. Prevalence of overweight and obesity among nurses in Scotland: A cross-sectional study using the Scottish Health Survey. Int J Nurs Stud 2016;53:126-33
12. Blake H, Harrison C. Health behaviours and attitudes towards being role models. British Journal of Nursing 2013;22:86-94
13. Dalton M. Implementing NICE obesity guidance for staff: an NHS trust audit. Occup Med 2014;65:78-85
14. Young A. Innovation within a national health care system. Surgery 2017;161:1179-82
15. World Health Organization. Healthy Workplaces: A Global Framework and Model: Review of Literature and Practices. Geneva, 2010. www.who.int/occupational_health/healthy_workplaces/en/index.html (accessed January 2018).
16. Jilcott Pitts S, Graham J, Mojica A, et al. Implementing healthier foodservice guidelines in hospital and federal worksite cafeterias: barriers, facilitators and keys to success. J Hum Nutr Diet 2016;29:677-86
17. NHS England 2017a. Commissioning for Quality and Innovation (CQUIN) Indicator Specification 2017-2019. https://www.england.nhs.uk/publication/cquin-indicator-specification/(accessed December 2017).
18. Al-Khudairy L, Uthman O, Johnson S, Clarke A, Oyebode O. Behavioural interventions to increase healthier purchasing and/or consumption of food and drink by NHS staff. PROSPERO 2017; CRD42017064872.

For peer review only - http://bmjopen.bmj.com/site/about/guidelines.xhtml

19. Hollands GJ, Shemilt I, Marteau TM, et al. Altering micro-environments to change: towards an evidence base for choice architecture interventions. BMC public health 2013;13:1218.
20. World Bank 2017. High Income. https://data.worldbank.org/income-level/high-income (accessed November 2017).
21. Willett W. Nutritional epidemiology: Oxford University Press 2012.
22. Higgins JPT, S G. Cochrane Handbook for Systematic Reviews of Interventions Version 5.1.0. The Cochrane Collaboration 2011.
23. Kim SY, Park JE, Lee YJ, et al. Testing a tool for assessing the risk of bias for nonrandomized studies showed moderate reliability and promising validity. J Clin Epidemiol 2013;66:408-14.
24. Geaney F, Harrington J, Fitzgerald AP, Perry IJ. The impact of a workplace catering initiative on dietary intakes of salt and other nutrients: a pilot study. Public Health Nutr 2011;14:1345-49.
25. Dorresteijn JA, van der Graaf Y, Zheng K, Spiering W, Visseren FL. The daily 10 kcal expenditure deficit: a before-and-after study on low-cost interventions in the work environment. BMJ open 2013;3:e002125.
26. Lassen AD, Beck A, Leedo E, et al. Effectiveness of offering healthy labelled meals in improving the nutritional quality of lunch meals eaten in a worksite canteen. Appetite 2014;75:128-34.
27. Meyers AW, Stunkard A, Coll M. Food accessibility and food choice. Arch Gen Psychiatry 1980;37:1133-35.
28. Milich R, Anderson J, Mills M. Effects of visual presentation of caloric values on food buying by normal and obese persons. Percept Mot Skills 1976;42:155-62.
29. Sato JN, Wagle A, McProud L, Lee L. Food label effects on customer purchases in a hospital cafeteria in northern California. Journal of foodservice business research 2013;16:155-68.
30. Thorndike AN, Riis J, Sonnenberg LM, Levy DE. Traffic-light labels and choice architecture: promoting healthy food choices. Am J Prev Med 2014;46:143-49.
31. Van Kleef E, Otten K, van Trijp HC. Healthy snacks at the checkout counter: A lab and field study on the impact of shelf arrangement and assortment structure on consumer choices. BMC public health 2012;12:1072.
32. Levy DE, Riis J, Sonnenberg LM, Barraclough SJ, Thorndike AN. Food choices of minority and low-income employees: a cafeteria intervention. Am J Prev Med 2012;43:240-48
33. Thorndike AN, Sonnenberg L, Riis J, Barraclough S, Levy DE. A 2-phase labeling and choice architecture intervention to improve healthy food and beverage choices. Am J Public Health 2012;102:527-33.
34. Lowe MR, Tappe KA, Butryn ML, et al. An intervention study targeting energy and nutrient intake in worksite cafeterias. Eating behaviors 2010;11:144-51.
35. Racette SB, Deusinger SS, Inman CL, et al. Worksite Opportunities for Wellness (WOW): Effects on cardiovascular disease risk factors after 1 year. Prev Med 2009;49:108-14.
36. Sorensen G, Stoddard A, Peterson K, et al. Increasing fruit and vegetable consumption through worksites and families in the Treatwell 5-a-Day study. Am J Public Health 1999;89:54-60.
37. Stites SD, Singletary SB, Menasha A, et al. Pre-ordering lunch at work. Results of the what to eat for lunch study. Appetite 2015;84:88-97.
38. MacDonald C, Genat B, Thorpe S, Browne J. Establishing health-promoting workplaces in Aboriginal community organisations: healthy eating policies. Aust. J. Prim. Health 2016;22:239-43

For peer review only

For peer review only - http://bmjopen.bmj.com/site/about/guidelines.xhtml

39. Hunt MK, Lederman R, Potter S, Stoddard A, Sorensen G. Results of employee involvement in planning and implementing the Treatwell 5-a-Day work-site study. Health Educ Behav 2000;27:223-31.
40. Hunt MK, Lederman R, Stoddard A, et al. Process tracking results from the Treatwell 5-a-Day Worksite Study. American Journal of Health Promotion 2000;14:179-87.
41. Sorensen G, Hunt MK, Cohen N, et al. Worksite and family education for dietary change: the Treatwell 5-a-Day program. Health Educ. *Res* 1998;13:577-91.
42. Vermeer WM, Steenhuis IH, Leeuwis FH, Heymans MW, Seidell JC. Small portion sizes in worksite cafeterias: do they help consumers to reduce their food intake?. Int. J. Obes 2011;35:1200.
43. Holdsworth M, Raymond NT, Haslam C. Does the Heartbeat Award scheme in England result in change in dietary behaviour in the workplace?. Health Promot Internation 2004;19:197-204.
44. Holdsworth M, Haslam C, Raymond N. Does the heartbeat award scheme change employees' dietary attitudes and knowledge?. Appetite 2000;35:179-88.
45. Vermeer WM, Leeuwis FH, Koprulu S, et al. The process evaluation of two interventions aimed at portion size in worksite cafeterias. J Hum Nutr Diet 2012;25:180-88.
46. Beresford SA, Thompson B, Feng Z, et al. Seattle 5 a Day worksite program to increase fruit and vegetable consumption. Prev Med 2001;32:230-38.
47. Sorensen G, Morris DM, Hunt MK, et al. Work-site nutrition intervention and employees' dietary habits: the Treatwell program. Am J Public Health 1992;82:877-80.
48. Kwak L, Kremers SP, Visscher TL, van Baak MA, Brug J. Behavioral and cognitive effects of a worksite-based weight gain prevention program: the NHF-NRG in balance-project. J Occup Environ Med 2009;51:1437-46.
49. Sorensen G, Hunt MK, Morris DH, et al. Promoting healthy eating patterns in the worksite: the Treatwell intervention model. Health Educ. Res 1990;5:505-15.
50. Cremaschini M, Moretti R, Brembilla G, et al. [Assessment of the impact over one year of a workplace health promotion programme in the province of Bergamo]. Med Lav 2015;106:159-71.
51. Glasgow RE, Terborg JR, Hollis JF, Severson HH, Boles SM. TAKE HEART - RESULTS FROM THE INITIAL PHASE OF A WORK-SITE WELLNESS PROGRAM. Am J Public Health 1995;85:209-16.
52. Verweij LM, Proper KI, Weel AN, Hulshof CT, van Mechelen W. The application of an occupational health guideline reduces sedentary behaviour and increases fruit intake at work: results from an RCT. Occup Environ Med 2012;69:500-7.
53. Vyth EL, Steenhuis IH, Heymans MW, et al. Influence of placement of a nutrition logo on cafeteria menu items on lunchtime food Choices at Dutch work sites. J Am Diet Assoc 2011;111:131-6.
54. Alinia S, Lassen AD, Krogholm KS, et al. A workplace feasibility study of the effect of a minimal fruit intervention on fruit intake. Public Health Nutr 2011;14:1382-7.
55. Dawson J, Dwyer JJM, Evers S, Sheeshka J. Eat smart! Workplace Cafeteria Program - Evaluation of the nutirition component. Can J Diet Pract Res 2006;67:85-90.
56. Kimathi AN, Gregoire MB, Dowling RA, Stone MK. A healthful options food station can improve satisfaction and generate gross profit in a worksite cafeteria. J Am Diet Assoc 2009;109:914-7.
57. Van Hulst A, Barnett TA, Dery V, Cote G, Colin C. Health-promoting vending machines: evaluation of a pediatric hospital intervention. Can J Diet Pract Res 2013;74:28-34.
58. Hurd J, Mitchell H, Hawkins C, Douglas E. The get on track challenge: An effective physical activity and healthy eating program for workplaces. Obesity Reviews 2014;15:221.

For peer review only

For peer review only - http://bmjopen.bmj.com/site/about/guidelines.xhtml

59. Kwak L, Kremers SP, Candel MJ, et al. Changes in skinfold thickness and waist circumference after 12 and 24 months resulting from the NHF-NRG In Balance-project. Int. J. Behav. Nutr. Phys. Act 2010;7:26.
60. Long MW, Tobias DK, Cradock AL, Batchelder H, Gortmaker SL. Systematic review and meta-analysis of the impact of restaurant menu calorie labeling. Am J Public Health 2015;105:e11-e24.
61. Sinclair SE, Cooper M, Mansfield ED. The influence of menu labeling on calories selected or consumed: a systematic review and meta-analysis. J. Acad. Nutr. Diet 2014;114:1375-88. e15.
62. Cecchini M, Warin L. Impact of food labelling systems on food choices and eating behaviours: a systematic review and meta-analysis of randomized studies. Obesity reviews 2016;17:201-10.
63. Sarink D, Peeters A, Freak-Poli R, et al. The impact of menu energy labelling across socioeconomic groups: A systematic review. Appetite 2016;99:59-75.
64. Adam A, Jensen JD. What is the effectiveness of obesity related interventions at retail grocery stores and supermarkets?—a systematic review. BMC public health 2016;16:1247.
65. Liberato SC, Bailie R, Brimblecombe J. Nutrition interventions at point-of-sale to encourage healthier food purchasing: a systematic review. BMC public health 2014;14:919.
66. Espino JNV, Guerrero N, Rhoads N, et al. Peer Reviewed: Community-Based Restaurant Interventions to Promote Healthy Eating: A Systematic Review. Prev. Chronic Dis 2015;12
67. Dorresteijn JAN, Van Der Graaf Y, Zheng K, Spiering W, Visseren FLJ. The daily 10 kcal expenditure deficit: A before-and-after study on low-cost interventions in the work environment. BMJ Open 2013;3:e002125.
68. Geaney F, Harrington J, Fitzgerald A, Perry I. The impact of a workplace catering initiative on dietary intakes of salt and other nutrients: a pilot study. Public Health Nutr 2011;14:1345-9.
69. Lassen AD, Beck A, Leedo E, et al. Effectiveness of offering healthy labelled meals in improving the nutritional quality of lunch meals eaten in a worksite canteen. Appetite 2014;75:128-34.
70. MacDonald C, Genat B, Thorpe S, Browne J. Establishing health-promoting workplaces in Aboriginal community organisations: healthy eating policies. Aust J Prim Health 2016;22:239-43.
71. Meyers AW, Stunkard AJ. Food accessibility and food choice: A test of Schachter's externality hypothesis. Arch Gen Psychiatry 1980;37:1133-35.
72. Beresford SA, Thompson B, Feng Z, et al. Seattle 5 a Day worksite program to increase fruit and vegetable consumption. Prev Med 2001;32:230-8.
73. Kwak L, Kremers SPJ, Candel M, et al. Changes in skinfold thickness and waist circumference after 12 and 24 months resulting from the NHF-NRG In Balance-project. Int. J. Behav. Nutr. Phys. *Act* 2010;7.
74. Kwak L, Kremers SP, Visscher TL, van Baak MA, Brug J. Behavioral and cognitive effects of a worksite-based weight gain prevention program: the NHF-NRG in balance-project. J Occup Environ Med 2009;51:1437-46.

For peer review only - http://bmjopen.bmj.com/site/about/guidelines.xhtml

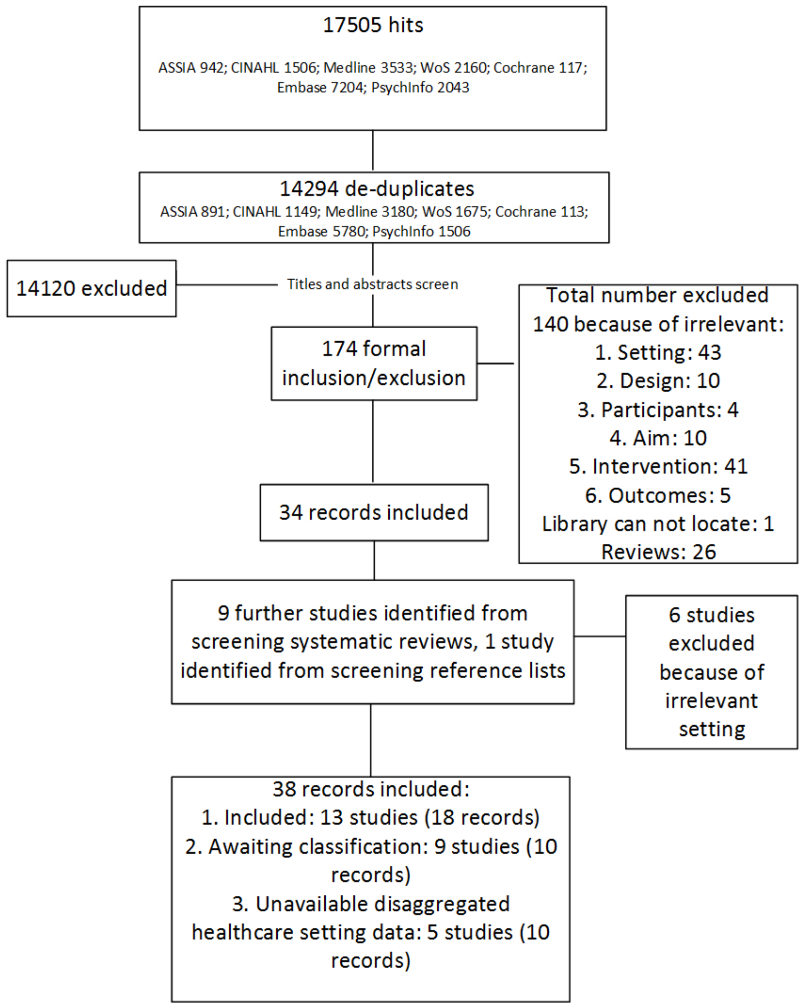

PRISMA flow diagram

90x90mm (300 x 300 DPI)

Search strategy used to interrogate MEDLINE

#	Searches	# of hits
35	limit 34 to ("all adult (19 plus years)" and humans)	2083
34	7 and 25 and 31	3734
33	limit 32 to ("all adult (19 plus years)" and humans)	4814
32	7 and 25 and 30	8677
31	26 or 27 or 28	2095764
30	26 or 27 or 28 or 29	2837423
29	health care.mp. or exp "Delivery of Health Care"/	1267298
28	exp Workplace/	17804
27	education, public health professional/ or exp "organization and administration"/	1248979
26	hospital*.mp.	1229950
25	18 or 24	537980
24	19 or 20 or 21 or 22 or 23	532969
23	eating behaviour.mp.	1262
22	Food habit*.mp.	1705
21	exp nutrition surveys/ or exp diet surveys/	21175
20	exp Nutrition Assessment/ or nutrition.mp. or exp Nutrition Surveys/	185218
19	exp Diet/ or Diet.mp.	396453
18	11 and 17	7544
17	12 or 13 or 14 or 15 or 16	1323348
16	drink*.mp.	153981
15	exp Meals/	3251
14	exp Food Preferences/	11931
13	exp beverages/ or exp food/	1173261
12	purchas*.mp.	26716
11	8 or 9 or 10	27711
10	Food Dispensers, Automatic/ or vending machine.mp.	388
9	sale*.mp.	14446
8	exp food services/ or exp food service, hospital/	13308
7	1 or 2 or 3 or 4 or 5 or 6	311674
6	choice architecture.mp.	42
5	exp Self Stimulation/ or exp Choice Behavior/ or exp Decision Making/ or nudge.mp.	173309
4	lifestyle intervention*.mp.	4193
3	exp life style/ or healthy lifestyle/	78107
2	behavio?r intervention*.mp.	528
1	exp health promotion/ or exp healthy people programs/ or weight reduction programs/	66897

For peer review only

**Which outcome?**	purchasing/dietary consumption/both *[delete as appropriate]*
**Reference (complete reference)**	
**Study ID (first author, year)**	
**Endnote No.**	
**Other reports of this trial: (Give EN No. First Author and Year)**	
**Reviewer:**	LA OO OU *[delete as appropriate]*
**Date of abstraction:**	

For studies awaiting classification skip results and quality assessment

For ongoing studies skip to the end of this form

Publication details

	[delete as appropriate]
**Language of publication**	English
**Funding source**	Commercial funding / non-commercial funding / other funding (specify)
**Publication status**	peer-reviewed journal / journal supplement / full article / other (specify)

Stated aims of study

**Quote from publication: "..."**	

**Notes**	

INFORMATION FOR CHARACTERISTICS OF INCLUDED STUDIES TABLE

	[delete as appropriate]
**Study design**	
**Participants:**	
**Brief description of participants/worksite**	
Inclusion criteria	
Exclusion criteria	
**Intervention:**	
**Number of worksites**	
**Country/authors location**	
**Setting (e.g. primary care, hospital)**	
**Type of health care setting** (such as NHS vs. non-NHS)	

For peer review only

**Description of intervention** (give details of all components)		
**Type of intervention (highlight and describe)**	Choice architecture only/choice architecture + complex intervention	
**Select intervention type**		
**Primarily alter properties or objective stimuli**	Ambiance – alter aesthetic or atmospheric aspects of the surrounding environment	
	Functional design – design or adapt equipment or function of the environment	
	Labelling – apply labelling or endorsement information to product or at point of choice	
	Presentation – alter sensory qualities or visual design of the product	
	Sizing – change size or quantity of the product	
	Pricing- change price of the product	
**Primarily alter placement or objects or stimuli**	Availability – add behavioural options within a given micro-environment	
	Proximity – make behavioural options easier (or harder) to engage with, requiring reduced (or increased) effort	
**Alter both properties and placement of objects or stimuli**	Priming – place incidental cues in the environment to influence a non-conscious behavioural response	
	Prompting – use non-personalised information to promote or raise awareness of a behaviour	
**Restaurant/canteen/vending machine/other (specify)**		
**Duration of intervention (duration of follow-up)**	Intervention:<br><br>Follow-up:	
**Comparator**	Yes/no	
**Type of comparator**	No intervention/wait list, different intervention, concomitant intervention, other ( )	
**Description of comparator**		
**Pre-intervention setup**	Yes/no	
**Description of pre-intervention setup**		
**Length of pre-intervention**		

OUTCOMES

NB: subjective measures of dietary intake **validated measures are eligible**. Any other measures list and note not data extracted.

**Tools description**	
**Dietary intake**	
**Dietary tool**	
**Purchasing data details**	

For peer review only

Baseline Characteristics

	**Intervention 1**	**Intervention 2**	**Comparator 1**	**Comparator 2**	**Overall**
**Sample size**					
**Staff group** (grade and/or category eg: medical vs nursing vs administrative)					
**Number of (*item*) sold**					
**Type of (*item*) sold** *[insert rows as needed]*					
**Type of (*item*) sold**					
**Type of (*item*) sold**					
**Price of (item) sold** *[insert rows as needed]*					
**Price of (item) sold** *[insert rows as needed]*					

***Purchasing data** [insert item name, add rows if required]*	**Intervention 1**	**Intervention 2**	**Comparator 1**	**Comparator 2**	***P values***
**Outcomes**					
**Sales data (%/n):** specify item					
At time point (specify)					
**Sales data (%/n):** specify item					
At time point (specify)					
**Sales data (%/n):** specify item					
At time point (specify)					
**Sales data (%/n):** specify item					
At time point (specify)					
**Sales data (%/n):** specify item					
At time point (specify)					
**Sales data (%/n):** specify item					
At time point (specify)					
**Narrative findings (please add *p* values):**					

Dietary data (do not extract total caloric intake, *insert item name, add rows if required]*

**Outcomes**	**Intervention 1**	**Intervention 2**	**Comparator 1**	**Comparator 2**
**Dietary outcomes** (specify, unit)				
Baseline	(mean, SD, N)			
At time point (specify)				
Change from baseline (specify which time point) reviewer to calculate if not reported[a]				
**Dietary outcomes** (specify, unit), mean (SD), number of participants:	(mean, SD, N)			
Baseline				
At time point (specify)				
Change from baseline (specify which time point) reviewer to calculate if not reported[a]				
**Dietary outcomes** (specify, unit), mean (SD), number of participants:	(mean, SD, N)			
Baseline				
At time point (specify)				
Change from baseline (specify which time point) reviewer to calculate if not reported[a]				
**Narrative findings (please add *p* values):**				

Notes of data to convert/calculations to be made (e.g. SEM to SD, change data, unavailable date):

Survey of study investigators providing information on included trials

**Study author contacted** [DD/MM/YY]	
**Study author replied** [DD/MM/YY]	

For peer review only

**Study author asked for additional information**<br><br>**[short summary]**	
**Study author provided data**<br><br>**[short summary]**	

RISK OF BIAS

**Risk of bias for RCTs**	*[delete as appropriate]*	**Support for judgement**
**Random sequence generation? (selection bias)**	Low risk of bias / High risk of bias / Unclear	Quote from publication: "..."
**Allocation concealment? (selection bias)**	Low risk of bias / High risk of bias / Unclear	Quote from publication: "..."
**Blinding of participants and personnel? (performance bias) Subjective outcomes**	Low risk of bias / High risk of bias / Unclear	Quote from publication: "..."
**Blinding of participants and personnel? (performance bias) Objective outcomes**	Low risk of bias / High risk of bias / Unclear	Quote from publication: "..."
**Blinding of outcome assessment? (detection bias) Subjective outcomes**	Low risk of bias / High risk of bias / Unclear	Quote from publication: "..."
**Blinding of outcome assessment? (detection bias) Objective outcomes**	Low risk of bias / High risk of bias / Unclear	Quote from publication: "..."
**Incomplete outcome data? (attrition bias) Subjective outcomes**	Low risk of bias / High risk of bias / Unclear	Quote from publication: "..."<br><br>Comment: no missing data / reported and reasons explained / only reported / not reported
**Incomplete outcome data? (attrition bias) Objective outcomes**	Low risk of bias / High risk of bias / Unclear	Quote from publication: "..."<br><br>Comment: no missing data / reported and reasons explained / only reported / not reported
**Selective outcome reporting?**	Low risk of bias / High risk of bias / Unclear	Comment
**Other bias?**	Low risk of bias / High risk of bias / Unclear	Comment

For peer review only

Risk of bias for non-RCTs	*[delete as appropriate]*	Support for judgement
**Selection of participants:** *Selection bias caused by the inadequate selection of participants*	Low risk of bias / High risk of bias / Unclear	Quote from publication: "..." Comment
**Confounding variables:** *Selection bias caused by the inadequate confirmation and consideration of confounding variable*	Low risk of bias / High risk of bias / Unclear	Quote from publication: "..." Comment
**Measurement of exposure:** *Performance bias caused by the inadequate measurement of exposure*	Low risk of bias / High risk of bias / Unclear	Quote from publication: "..." Comment
**Blinding of outcome assessments (subjective outcomes)** *Detection bias caused by the inadequate blinding of outcome assessments*	Low risk of bias / High risk of bias / Unclear	Quote from publication: "..." Comment
**Blinding of outcome assessments (objective outcomes)** *Detection bias caused by the inadequate blinding of outcome assessments*		
**Incomplete outcome data (subjective outcomes)** *Attrition bias caused by the inadequate handling of incomplete outcome data*	Low risk of bias / High risk of bias / Unclear	Quote from publication: "..." Comment
**Incomplete outcome data (objective outcomes)** *Attrition bias caused by the inadequate handling of incomplete outcome data*		
**Selective outcome reporting:** *Reporting bias caused by the selective reporting of outcomes*	Low risk of bias / High risk of bias / Unclear	Quote from publication: "..." Comment

Ongoing studies

For peer review only

**Study name**	Acronym:	
**Methods**	Study design:	
	Type of health care setting:	
	Country:	
**Participants**	Enrollment: (e.g. estimated 40)	
	Inclusion criteria:	
	Exclusion criteria:	
**Interventions**	Intervention(s):	
	Comparator(s):	
**Outcomes**	OUTCOME(S)	
**Starting date**	Study start date:	
	Study completion date:	
**Contact information**	Responsible party/principal investigator:	
**Study identifier** (if applicable)	(e.g.) NCT number:	
**Official title**		
**Stated purpose of study** (aim)	Quote: "..."	
**Notes**		

For peer review only

Evidence-based environmental changes to promote healthy eating and drinking by NHS staff

The size of the problem

- The leading risk behaviour for disability and death in England is poor diet[1].
- Disease associated with poor diet costs the NHS at least £5.8 billion per year[2].
- Nearly two thirds of adults in England are now overweight or obese[3].
- The overall cost of obesity to wider society was projected to reach £27 billion in 2015[4].

Barriers to healthy eating specifically reported by health care staff [13,14,15,16,17]

- Lack of time;
- Unpredictable/demanding workload;
- Prioritising patients workload;
- Inconvenient access to food (both physically ie: a long walk to where food is served, and in time ie: 24 hour duties but limited opening times.
- Lack of availability of food they want to eat.

The importance to the NHS

- The NHS is the fifth largest employer on the planet, and the largest employer in the UK[5]. Improving the health and wellbeing of the 1.4 million NHS staff will lift the health and wellbeing levels of the entire UK population.
- Obesity is associated with absenteeism, presenteeism, early retirement, injuries, discrimination, and litigation[6,7,8]. NHS staff absence due to poor health costs at least £2.4billion a year[9]. Improving employee health will improve productivity and performance of the workforce leading to improved patient care.
- Health professionals play an important role in health promotion for the 1 million patients seen in the NHS every 36 hours. Many staff feel more able to do this when they appear to be following health advice themselves[10,11]. By taking action to address diet and obesity for NHS staff, the organisation and staff will be role models for others.
- Policy drivers include NICE Guidance on obesity prevention in workplaces (CG43); NHS standard contract; NHS CQUIN Indicator: Healthy food for NHS staff, visitors and patients; NHS England voluntary sugar-sweetened beverage target [Appendix 1].

Evidence based environmental changes that work to improve diets

Changing the environment to promote healthy behaviour- such as healthy food and drink purchasing and consumption can be particularly useful and effective because:

- When the environment makes healthy choices easier, it doesn't require any effort by the target audience. This means it isn't just those people who are highly motivated to make healthy changes who benefit.
- Most changes are no-cost or low cost to introduce.

Providing information alone (for example, labelling food to indicate that it is healthy or its calorie content) is unlikely to improve diet, although this can work alongside other environmental changes.

More benefits to making these changes

- Greater availability of healthy food can improve the general attractiveness of a food provider[12].Customer volume and sales may increase after changes to improve healthy food and drink purchasing are made[13,14] Types of change for which there is evidence of effectiveness:

Type of change	Examples
Reduce the effort required to select healthy options, or increase the effort required to select unhealthy options.	➢ Healthier vegetable spread available in easy-to-reach positions, butter stored in a fridge.<br>➢ Desserts arranged so that fresh fruit salad was situated in the easiest access positions, cakes and pies in the least convenient.<br>➢ Rearrangement of the canteen to increase the prominence of fruit and water and reduce prominence of confectionery and crisps.
Increase availability of healthy options.	➢ Snacks on offer at a hospital canteen enforced at 75% healthy, no more than 25% unhealthy.<br>➢ Healthy snack cart that visited staff.
Offer smaller sizes of main meals and other products.	➢ Two size options for the main meal offered in a hospital canteen.

Other examples of successful initiatives include:

- ➢ An online pre-ordering system for lunch, which allowed staff to choose their lunch an hour or more before collecting it at the canteen without joining a queue. Nutritional information was presented and the system defaulted to the healthier option when appropriate.
- ➢ Traffic light labelling of all items on offer, with green (healthy) items placed in the most salient positions and red (unhealthy) items made less visible. Permanent changes to menu boards and other signage to highlight the healthiest options.

References

For more details of the systematic review that supported this document, please contact o.r.o.oyebode@warwick.ac.uk.

1. Newton JN, Briggs AD, Murray CJ, et al. Changes in health in England, with analysis by English regions and areas of deprivation, 1990–2013: a systematic analysis for the Global Burden of Disease Study 2013. *The Lancet* 2015;386:2257-74.
2. Scarborough P, Bhatnagar P, Wickramasinghe KK, et al. The economic burden of ill health due to diet, physical inactivity, smoking, alcohol and obesity in the UK: an update to 2006–07 NHS costs. *J Public Health* 2011;33:527-35.
3. Health and Social Care Information Centre, 2016. Health Survey for England 2015: Adult overweight and obesity. NHS Digital. https://digital.nhs.uk/catalogue/PUB20562 (accessed November 2017).
4. Butland B, Jebb S, Kopelman P, et al. Tackling obesities: future choices-project report. London: Department of Innovation, Universities and Skills 2007; 10:17.
5. Young A. Innovation within a national health care system. *Surgery* 2017;161:1179-82.
6. Shrestha N, Pedisic Z, Neil-Sztramko S, Kukkonen-Harjula KT, Hermans V. The impact of obesity in the workplace: a review of contributing factors, consequences and potential solutions. *Current obesity reports* 2016;5:344-60.
7. Koepp GA, Snedden BJ, Levine JA. Workplace slip, trip and fall injuries and obesity. *Ergonomics* 2015;58:674-79.
8. Flint SW, Snook J. Disability discrimination and obesity: the big questions? *Current obesity reports* 2015;4:504-09
9. Blake H, Mo PK, Lee S, Batt ME. Health in the NHS: lifestyle behaviours of hospital employees. *Perspectives in public health* 2012;132:213.
10. Dalton M. Implementing NICE obesity guidance for staff: an NHS trust audit. *Occup Med* 2014;65:78-85.
11. Van Kleef E, Otten K, van Trijp HC. Healthy snacks at the checkout counter: A lab and field study on the impact of shelf arrangement and assortment structure on consumer choices. *BMC public health* 2012;12:1072.
12. Jilcott Pitts S, Graham J, Mojica A, et al. Implementing healthier foodservice guidelines in hospital and federal worksite cafeterias: barriers, facilitators and keys to success. J Hum Nutr Diet 2016;29:677-86.
13. Kimathi AN, Gregoire MB, Dowling RA, Stone MK. A healthful options food station can improve satisfaction and generate gross profit in a worksite cafeteria. J Am Diet Assoc 2009;109:914-7

For peer review only

For peer review only

Reporting checklist for systematic review and meta-analysis.

Based on the PRISMA guidelines.

Instructions to authors

Complete this checklist by entering the page numbers from your manuscript where readers will find each of the items listed below.

Your article may not currently address all the items on the checklist. Please modify your text to include the missing information. If you are certain that an item does not apply, please write "n/a" and provide a short explanation.

Upload your completed checklist as an extra file when you submit to a journal.

In your methods section, say that you used the PRISMA reporting guidelines, and cite them as:

Moher D, Liberati A, Tetzlaff J, Altman DG, The PRISMA Group. Preferred Reporting Items for Systematic Reviews and Meta-Analyses: The PRISMA Statement

		Reporting Item	Page Number
	#1	Identify the report as a systematic review, meta-analysis, or both.	1
Structured summary	#2	Provide a structured summary including, as applicable: background; objectives; data sources; study eligibility criteria, participants, and interventions; study appraisal and synthesis methods; results; limitations; conclusions and implications of key findings; systematic review registration number	2
Rationale	#3	Describe the rationale for the review in the context of what is already known.	3
Objectives	#4	Provide an explicit statement of questions being addressed with reference to participants, interventions, comparisons, outcomes, and study design (PICOS).	4
Protocol and registration	#5	Indicate if a review protocol exists, if and where it can be accessed (e.g., Web address) and, if available, provide registration information including the registration number.	1,4

For peer review only

Eligibility criteria	#6	Specify study characteristics (e.g., PICOS, length of follow-up) and report characteristics (e.g., years considered, language, publication status) used as criteria for eligibility, giving rational	4-5
Information sources	#7	Describe all information sources in the search (e.g., databases with dates of coverage, contact with study authors to identify additional studies) and date last searched.	5
Search	#8	Present full electronic search strategy for at least one database, including any limits used, such that it could be repeated.	Supplementary 1
Study selection	#9	State the process for selecting studies (i.e., for screening, for determining eligibility, for inclusion in the systematic review, and, if applicable, for inclusion in the meta-analysis).	5
Data collection process	#10	Describe the method of data extraction from reports (e.g., piloted forms, independently by two reviewers) and any processes for obtaining and confirming data from investigators.	5
Data items	#11	List and define all variables for which data were sought (e.g., PICOS, funding sources), and any assumptions and simplifications made.	Supplementary 2
Risk of bias in individual studies	#12	Describe methods used for assessing risk of bias in individual studies (including specification of whether this was done at the study or outcome level, or both), and how this information is to be used in any data synthesis.	5
Summary measures	#13	State the principal summary measures (e.g., risk ratio, difference in means).	5
Planned methods of analyis	#14	Describe the methods of handling data and combining results of studies, if done, including measures of consistency (e.g., I2) for each meta-analysis.	5
Risk of bias across studies	#15	Specify any assessment of risk of bias that may affect the cumulative evidence (e.g., publication bias, selective reporting within studies).	25-29

For peer review only

Additional analyses	#16	Describe methods of additional analyses (e.g., sensitivity or subgroup analyses, meta-regression), if done, indicating which were pre-specified.	5
Study selection	#17	Give numbers of studies screened, assessed for eligibility, and included in the review, with reasons for exclusions at each stage, ideally with a flow diagram.	6, PRISMA flow
Study characteristics	#18	For each study, present characteristics for which data were extracted (e.g., study size, PICOS, follow-up period) and provide the citation.	14-20
Risk of bias within studies	#19	Present data on risk of bias of each study and, if available, any outcome-level assessment (see Item 12).	25-29
Results of individual studies	#20	For all outcomes considered (benefits and harms), present, for each study: (a) simple summary data for each intervention group and (b) effect estimates and confidence intervals, ideally with a forest plot.	21-22
Synthesis of results	#21	Present the main results of the review. If meta-analyses are done, include for each, confidence intervals and measures of consistency.	6-9
Risk of bias across studies	#22	Present results of any assessment of risk of bias across studies (see Item 15).	9
Additional analysis	#23	Give results of additional analyses, if done (e.g., sensitivity or subgroup analyses, meta-regression [see Item 16]).	22
Summary of Evidence	#24	Summarize the main findings, including the strength of evidence for each main outcome; consider their relevance to key groups (e.g., health care providers, users, and policy makers	10,11
Limitations	#25	Discuss limitations at study and outcome level (e.g., risk of bias), and at review level (e.g., incomplete retrieval of identified research, reporting bias).	10
Conclusions	#26	Provide a general interpretation of the results in the context of other evidence, and implications for future research.	10
Funding	#27	Describe sources of funding or other support (e.g., supply	12-13

For peer review only

of data) for the systematic review; role of funders for the systematic review.

The PRISMA checklist is distributed under the terms of the Creative Commons Attribution License CC-BY. This checklist can be completed online using https://www.goodreports.org/, a tool made by the EQUATOR Network in collaboration with Penelope.ai

For peer review only

BMJ Open

Choice architecture interventions to improve diet and/or dietary behaviour by Healthcare staff in high-income countries: a systematic review

Journal:	*BMJ Open*
Manuscript ID	bmjopen-2018-023687.R1
Article Type:	Research
Date Submitted by the Author:	28-Sep-2018
Complete List of Authors:	Al-Khudairy, Lena; University of Warwick, Population, evidenec and technology<br>Uthman, Olalekan; Warwick Medical School,<br>Walmsley, Rosemary; University of Oxford<br>Johnson, Samantha ; University of Warwick Warwick Medical School<br>Oyebode, Oyinlola; University of Warwick Warwick Medical School, Population, evidence and technology. Division of Health Sciences
**Primary Subject Heading**:	Public health
Secondary Subject Heading:	Nutrition and metabolism, Public health, Health services research, Health policy
Keywords:	choice architecture, diet, health care, PUBLIC HEALTH

SCHOLARONE™
Manuscripts

Title Page

Choice architecture interventions to improve diet and/or dietary behaviour by Healthcare staff in high-income countries: a systematic review.

Authors' names: Lena Al-Khudairy[1], Senior Research Fellow; Olalekan Uthman[1], Assistant Professor; Rosemary Walmsley[1,2], Research Assistant; Samantha Johnson[1], Academic Support Librarian; Oyinlola Oyebode[1], Associate Professor in Public Health.

Address for each author: 1. Warwick Medical School, University of Warwick, Gibbet Hill Campus, Coventry, UK. CV4 7AL.

2. Worcester College, University of Oxford, Oxford, UK. OX1 2HB.

Correspondence to: O.R.O.Oyebode@warwick.ac.uk

For peer review only

For peer review only - http://bmjopen.bmj.com/site/about/guidelines.xhtml

Abstract

Objectives: we were commissioned by the behavioural insights team at Public Health England to synthesise the evidence on choice architecture interventions to increase healthy purchasing and/or consumption of food and drink by NHS staff. **Data sources:** MEDLINE, EMBASE, CINAHL, Cochrane Central register of Controlled Trials, PsycINFO, Applied Social Sciences Index and Abstracts and Web of Science were searched from inception until May 2017 and references were screened independently by two reviewers. **Design:** a systematic review that included randomised experimental or intervention studies, interrupted time series and controlled before and after studies. **Participants:** healthcare staff of high income countries. **Intervention:** choice architecture interventions that aimed to improve dietary purchasing and/or consumption **(outcomes)** of staff. **Appraisal and synthesis:** eligibility assessment, quality appraisal, data abstraction and analysis were completed by two reviewers. Quality appraisal of randomised trials was informed by the Cochrane Handbook and the Risk of Bias Assessment Tool for Nonrandomized Studies was used for the remainder. Findings were narratively synthesised. **Results:** Eighteen studies met the inclusion criteria. Five studies included multiple workplaces (including health care settings), 13 were conducted in health care settings only. Interventions in 10 studies were choice architecture only and 8 studies involved a complex intervention with a choice architecture element. Interventions involving a proximity element (making behavioural options easier or harder to engage with) appear to be frequently effective at changing behaviour. One study presented an effective sizing intervention. Labelling alone was generally not effective at changing purchasing behaviour. Interventions including an availability element were generally reported to be successful at changing behaviour but no included study examined this element alone. There was no strong evidence for the effect of pricing on purchasing or dietary intake. **Conclusion:** proximity, availability and sizing are choice architecture elements that are likely to be effective for NHS organisations. **Registration number:** CRD42017064872.

Strengths and limitations of the study

- Examines interventions appropriate to improve diet in this important and unique population group: health care staff.
- Potential bias in identification of studies was minimised through a comprehensive search across major electronic databases relevant to the topic of this review, reference lists of relevant studies were screened and no language restriction was applied.
- To increase methodological rigour and minimise error and bias, each study included in this review was selected, assessed, data extracted, and quality assessed by two review authors independently.
- Many included studies examined complex interventions and it is not always possible to draw conclusions about the contribution of specific choice architecture elements to the outcomes.

Key words choice architecture, diet, health care

Word count 4981

For peer review only - http://bmjopen.bmj.com/site/about/guidelines.xhtml

Introduction

The leading risk behaviour for disability and death in England, as in other high income countries, is poor diet [1]. Worldwide, the proportion of adults who are overweight has increased to 36.9% of men and 38.0% in women [2]. Disease associated with poor diet costs the National Health Service (NHS) at least £5.8 billion per year [3]. Nearly two thirds of adults in England are now overweight or obese [4]. The overall cost of obesity to wider society was projected to reach £27 billion in 2015 [5].

Health care settings have an important role to play in helping their staff tackle this issue. Firstly, obesity is associated with absenteeism, presenteeism, early retirement, injuries, discrimination, and litigation [6-8]. In England, NHS staff absence due to poor health costs at least £2.4 billion a year [9]. Improving employee health may improve productivity and performance of the workforce leading to improved patient care. Secondly, health professionals play an important role in health promotion. However, a high proportion of healthcare staff do not engage in positive health behaviours themselves [10 ,11]. Nurses with poor health behaviour may be less likely to carry out health promotion activity [12]. A questionnaire survey of 540 pre-registered nurses found that three quarters felt that their physical appearance affected how they were perceived as a nurse [12]. Most respondents felt that patients would be more likely to heed their advice on health behaviours if they appeared to be following that advice themselves. 79.1% agreed that nurses should present themselves as role models for health behaviours [12]. Similarly a questionnaire completed by occupational health staff found that a fifth of them felt their own weight made it difficult to address the issue in patients [13]. By taking action to address diet and obesity for healthcare staff, these organisations and staff will be role models for others. While these are good reasons for any health care setting taking a role in supporting staff to eat healthily in the UK, an additional reason is that the NHS is the largest employer [14]. This means that improving the health and wellbeing of the 1.4 million NHS staff will lift the health and wellbeing levels of the entire UK population.

The WHO defined a healthy workplace as "one in which workers and managers collaborate to use a continual improvement process to protect and promote the health, safety and well-being of all workers and the sustainability of the workplace" [15]. There are initiatives around the world to improve workplace wellbeing and many of these are specific to health care settings [16]. In England, there are a number of policy drivers to encourage and support NHS organisations to improve the food and drink environment including an NHS Commissioning for Quality and Innovation Indicator: Healthy food for NHS staff, visitors and patients [17]. Changing the environment to promote healthy behaviour- such as healthy food and drink purchasing and consumption can be particularly useful and effective because:

- When the environment makes healthy choices easier, this doesn't require any effort by the target audience. This means it isn't just those people who are highly motivated to make healthy changes who benefit.
- Most changes are no-cost or low cost to introduce.

We were commissioned by the behavioural insights team at Public Health England (PHE) to undertake a systematic review and evidence synthesis, examining the literature to inform the evidence base on choice architecture interventions to increase healthier purchasing and/or consumption of food and drink by NHS staff.

For peer review only - http://bmjopen.bmj.com/site/about/guidelines.xhtml

Materials and Methods

The protocol for this systematic review was registered with PROSPERO (an international database of prospectively registered systematic reviews in health and social care) on 1st May 2017 CRD42017064872 [18].

MEDLINE, EMBASE, CINAHL, Cochrane Central register of Controlled Trials, PsycINFO, Applied Social Sciences Index and Abstracts, and Web of Science were searched from inception until May 2017. Reference lists of retrieved included studies and of identified relevant systematic reviews were scanned for any additional studies not picked up by our electronic searches. No restrictions were made by language or year of publication. The search terms used included medical subject headings (MeSH) or the equivalent, and text word terms (*i.e.*, health promotion, choice architecture, food services, meals, workplace). A specialist librarian (SJ) was consulted for further search terms. Searches were tailored to individual databases (please see supplementary material 1).

Studies met the inclusion criteria if they fulfilled all of the following:

Study design - randomised experimental or intervention studies, including cluster randomised trials, quasi-randomised studies, interrupted time series studies (before and after) and controlled before and after studies;

Participants - NHS staff or staff from any other health care setting in a high-income country context. We used the World Bank classification for high-income countries, which includes 79 countries in total [19].

Interventions - Behavioural interventions that aimed to change dietary purchasing and/or consumption of staff were included. We restricted the specific behavioural interventions examined to "choice architecture" or “nudge” or “design” interventions. We used Hollands [20] definition of choice architecture interventions- “those that involve altering small-scale physical and social environments, or micro-environments to cue healthier behaviour” and the associated framework to structure this review. We expanded this framework to include interventions that altered the price of items to cue healthier behaviour (Table 1). This framework was chosen in discussion with PHE, as it has previously been used to examine dietary interventions and was applicable to the context (health care settings).

Outcomes - a) dietary (food and/or drink) purchasing (e.g. sales data; receipts analysis) in restaurants, canteens, vending machines or other situations in which food and drinks are sold; or b) dietary intake assessed by validated tools or by more than one dietary measure such as multiple 24-hour dietary recalls. We did not examine caloric intake as self-reported measures showed poor correlation in comparison to objective measures for caloric intake [21].

Table 1: Typology of choice architecture intervention in micro-environments, modified from Hollands et al (2013) [20]

Intervention Class	Intervention Type
Primarily alter properties of objects or stimuli	Ambience- alter aesthetic or atmospheric aspects of the surrounding environment
	Functional Design- design or adapt equipment or function of the environment
	Labelling- apply labelling or endorsement information to product or at point-of-choice
	Presentation- alter sensory qualities or visual design of the product
	Sizing- change size or quantity of the product

For peer review only - http://bmjopen.bmj.com/site/about/guidelines.xhtml

	Pricing- change price of the product
Primarily alter placement of objects or stimuli	Availability- add behavioural options within a given micro-environment
	Proximity- make behavioural options easier or harder to engage with, requiring reduced or increased effort
Alter both properties and placement of objects or stimuli	Priming- place incidental cues in the environment to influence a non-conscious behavioural response
	Prompting- use non-personalised information to promote or raise awareness of a behaviour

Following the database searching, titles, abstracts or both of every record retrieved were screened for potential relevance by two review authors (LA and OO) independently. Studies not carried out in high-income countries, qualitative studies and studies of children were excluded at this stage. Following this preliminary screening, full reports of potentially relevant studies were obtained, and two reviewers (two of LA, OO, OU) independently assessed studies for inclusion/exclusion using a checklist form based on the four inclusion criteria above. Where there was disagreement about the inclusion of a study, a third reviewer was consulted (LA or OO or OU). Where resolution of a disagreement was not possible, we added the article to those ’awaiting assessment’ and contacted study authors for clarification. Data were extracted from the included studies by two reviewers (two of LA, OO, OU, RW) independently using a predefined and pre-piloted data abstraction form (please see supplementary material 2). Key data including details on methods, participants, intervention (including mapping to framework domains), outcomes, funding and notable conflicts of interest of study authors. The assessment of the methodological quality of included randomised trials was informed by guidelines from the Cochrane Handbook [22]. Risk of bias for other quantitative study designs used the Risk of Bias Assessment Tool for Nonrandomized Studies [23].

Patient and Public Involvement

As this was a themed commissioned piece of work, patient and public advisors were not involved in the development of the research question, design of the study or conduct of the study. We were required to develop a toolkit (supplementary material 3) in a companion format designed for the audience who make decisions about catering in NHS organisations. Two advisors (please see acknowledgement) reviewed the toolkit to ensure its relevance to the target audience.

Results

Searching electronic databases generated 17505 hits which equated to 14294 individual records after duplicates were removed. Screening of titles and abstracts for potential relevance excluded 14120 records, as they did not meet the above mentioned inclusion criteria of this review, leaving 174 potentially relevant records. Screening relevant systematic reviews and included studies references yielded an additional 10 records. After formal inclusion/exclusion of 184 records, we identified 28 records from 18 studies met the inclusion criteria of this review. This included nine records from five studies in which the settings were not exclusively hospitals or other health care workplaces, but in which at least one of the sites involved was relevant to our review. In addition we noted ten records from nine studies where we were unable to ascertain whether the study met our inclusion criteria based on information included in the study reports, and author contacts received no replies. In these cases, studies were classified as “awaiting classification” [Figure 1].

For peer review only - http://bmjopen.bmj.com/site/about/guidelines.xhtml

Results were narratively synthesised due to the substantial heterogeneity in study design, interventions (even when classified within the same framework category), and outcomes reported. We were unable to proceed with planned meta-analysis or subgroup analyses [18].

Included studies

Health care settings

Key characteristics of 13 included published studies are presented in Table 2. Eight studies [24-33] examined pure choice architecture interventions and five studies [34-41] examined complex interventions for which some elements were choice architecture. One included study was conducted in the UK [24], three in other European countries [25 ,26 ,31], eight in the USA [27-30 ,32-37 ,39-41] and one in Australia [38]. Eight studies were interrupted time series [25 ,27-34 ,38], two were cluster randomised controlled trials [35 ,36 ,39-41], one was an RCT [37], one was a controlled before and after study [26] and one cross-sectional study [24]. Seven studies examined purchasing outcomes [25, 27-31, 38], five examined dietary consumption [24, 26, 35-37, 39-41] and one study examined both purchasing and dietary consumption [34].

Multiple workplaces

Five studies (table 2) in which settings included multiple workplaces and data were not disaggregated for health care settings were included in this review. Two studies [42-45] examined pure choice architecture interventions and three studies [46-49] examined complex interventions for which some elements were choice architecture. Two studies were conducted in the USA [46 ,47], two in The Netherlands [42 ,45 ,48] and one in the UK [43 ,44]. Three studies were cluster RCTs [42 ,45-47 ,49], one was a controlled before and after study [48] and one was a cross-sectional study [43 ,44]. Four studies examined dietary consumption [43 ,44 ,46-48] while one examined purchasing [42 ,45]. Further details of included studies are available in table 2.

Awaiting classification

Despite contacting authors we were unable to classify nine studies because sufficient details were lacking in study reports. Five studies were unclear on the setting [50-54]; two studies were unclear on the outcomes [55 ,56]; one study was unclear on the type of participants [57] and one abstract was unclear on several domains [58].

Intervention components and effectiveness

We examined effectiveness of interventions in included studies by whether they were a choice architecture only intervention, or a complex intervention with choice architecture elements. Summaries of the components included in interventions mapped against their reported effectiveness are presented in Tables 3 and 4.

Pure choice architecture

Of the choice architecture only interventions, almost all included studies involved labelling elements, including four studies that examined labelling in isolation. The most frequently studied single element choice architecture interventions were proximity interventions, investigated in five studies [24 ,25 ,27 ,30 ,31]. No included study investigated ambience or priming elements (Table 3). Most of the identified published studies reported successful interventions.

There was mixed evidence for the effectiveness of labelling interventions implemented on their own. Labelling interventions were not found to have an effect on purchasing in two studies [25 ,29],

For peer review only - http://bmjopen.bmj.com/site/about/guidelines.xhtml

although they were reported to change purchasing behaviour in one other study [28]. In the study that reported successfully changing purchasing through labelling, every item of food on offer in a hospital canteen was labelled with its caloric value. This had a significant effect on the number of calories purchased during the intervention phase. This study was at low risk of bias in three domains, however, it is more than four decades old and therefore likely to be examining a slightly different context to present day [28]. There was a low risk of bias in one of the studies that found no effect of labelling [25]. In this study lean (30% less fat) and butter croissants which were similar in price, appearance and taste were on offer in a Dutch hospital restaurant, where they are a popular choice for lunch. During the intervention period, the lean basket was labelled with a sign that stated “The saturated (unhealthy) fat content of this croissant is 30% lower”, however there was statistically significant difference between the number or ratio of the two alternative products purchased during the intervention period, compared with the control periods [25]. Another study labelled the two entrees on offer in a hospital canteen (one of which was designed to be healthier) with information on their constituent calories, fat, and sodium but this did not significantly change sales of the healthier option [29]. This study had a high risk of bias from confounding [29]. Overall it appears that labelling is unlikely to be effective on its own.

One study, with a low risk of bias in three domains, examined an intervention which used sizing alone, specifically, offering a smaller size of the main meal of the day, which was successful in terms of changing purchasing behaviour- about 10% of hot meals sold were small meals, with no overall change in total hot meals sold and no compensatory purchasing, for instance purchasing the smaller hot meal and adding a portion of chips) [42 ,45]. A pricing element was also evaluated in this study, in which the smaller meal was either priced proportionately so that both size and price of the meal were 65% of the standard; or ‘value’ pricing was used in which the size of the meal was 65% of the standard, but the price only reduced to 80% of the standard. The pricing condition did not change the effectiveness of the intervention, there was no significant different between the proportionate and value pricing conditions [42 ,45].

Proximity interventions alone were successful at changing purchasing behaviour in two studies [25 ,27]. The interventions included having margarine available in four centrally located and easy-to-reach positions in the hospital restaurant, but butter stored in a fridge, therefore requiring more effort to obtain which reduced sales of butter and increase sales of margarine [25]; re-arrangement of desserts in a hospital canteen so that low-calorie desserts (fruit and jelly) or high-calorie desserts (pies and cakes) were at the front in most accessible positions [27].

Other successful pure choice architecture interventions including more than one element were:

- Labelling + availability interventions which changed dietary consumption in two studies [26 ,43 ,44]. Both these studies involved stipulating the proportion of meals on offer in a canteen that should be healthy (half in Lassen et al, 2014; a third in Holdsworth et al, 2014) and labelling these healthy meals as such. These are the only two pure choice architecture interventions which assessed dietary consumption. However both had a high risk of bias over more than one domain.

- Labelling + proximity + prompting which changed purchasing behaviour in one study with a low risk of bias over most domains [30]. In this study all items on offer were labelled according to a traffic light system, green items were put at eye-level while red items were made less visible, and permanent signage and menu board changes were made to highlight healthy options.

For peer review only - http://bmjopen.bmj.com/site/about/guidelines.xhtml

- Availability + proximity intervention which changed purchasing behaviour in one study with a low risk of bias [31]. In this study a set of shelves stocked with snacks was placed on the check-out counter of a hospital canteen. In two phases, the shelves were stacked with 75% healthy and 25% unhealthy snacks and for the other two phases these proportions were reversed. In addition, the shelves were re-arranged so that the healthy snacks were at the top in two phases, and at the bottom (requiring stooping to access) in the other two phases. Both availability (proportion of healthy snacks on offer) and proximity (ease of obtaining the healthy snacks) had a positive effect on healthy snack purchasing.

Finally, Geaney et al (2011)[24] used an intervention with several choice architecture elements which was successful in changing dietary consumption. In this study menus were modified to increase availability of healthy options: high-salt products and processed meat were replaced with low-salt options, all desserts were fruit based, cooking methods with oil were limited. In addition salt was removed in all cooking processes and from tables in the staff canteen (although it was available in small sachets from service). Nutritional information on salt reduction and a healthy diet was displayed in the canteen area. No sauces or accompaniments were added to any meals without the customer's consent and extra salad and vegetable options was provided at no extra cost. This study had a low risk of bias and demonstrated an effect on 24 hour dietary recall data [24].

The only unsuccessful multi-component choice architecture only intervention was examined in Dorresteijn et al, (2013) [25] which used labelling + proximity + prompting. In this case, the two soups usually on offer in the restaurant were altered so that one option every day was reduced salt. Signs advertised that this soup "contains 30% less salt and contributes to a healthy blood pressure" however, this did not affect the number or ratio of normal salt to reduced-salt soup cups sold.

Complex interventions including choice architecture

As with the choice architecture only interventions, most of the complex interventions included labelling elements in studied interventions while none included ambience or priming elements. In addition none of the included studies of complex interventions examined presentation or sizing interventions. Where choice architecture interventions were implemented as part of complex interventions (usually involving other elements of behaviour change eg: educational elements) there were many examples of effective interventions [Table 4].

Complex interventions involving an availability choice architecture element were effective at changing dietary consumption in two studies [35 ,46]. In one of these studies, the other elements of the intervention were extensive including pedometers, on-site WeightWatchers® group meetings, an on-site group exercise program, monthly lunchtime seminars, monthly newsletters, walking maps, team competitions, participation cards, and participation rewards and weekly access to a registered dietitian/exercise specialist [35]. The environmental component of the intervention was a weekly healthy snack cart that visited staff [35]. In the other study, the intervention included formation of employee advisory boards in each worksite to implement activities in their workplace including education and information about 5-a-day, and a self-help manual provided to every employee to support them making healthy choices. In addition the "availability" component was providing more fruits and vegetables as part of the regular menus in the worksite canteens [46]. In both cases it is difficult to draw much of a conclusion about the "availability" components compared with the other components of the intervention.

Labelling + prompting was a successful combination when used alongside a complex intervention in one study [38] and labelling + prompting + proximity was effective as part of a complex intervention

For peer review only - http://bmjopen.bmj.com/site/about/guidelines.xhtml

in Sorensen et al (1992)[47 ,49]. However labelling + proximity, without prompting, was not effective in another study [36 ,39-41]. In all three studies the other (non-choice-architecture) elements of the interventions were direct educational activities, but none were particularly intensive suggesting labelling + prompting might work as pure choice-architecture (labelling + prompting + proximity was also effective in the choice architecture only studies [30].

Labelling + pricing + availability was effective when used alongside a complex intervention in one study [34]. In this study canteens modified the recipes of some foods offered in order to reduce the energy density and introduced nutritional labels for all foods. In addition some employees were given training in reducing the energy density of their diet and discounts on lower energy dense foods bought in the canteen [34]. This study had high risk of attrition bias and possible evidence of selective outcome reporting. The study reported a significant reduction in total kilocalories purchased in the canteen and in the proportion of energy from fat [34].

Stites et al (2015)[37] used a complex intervention with many choice architecture elements and was effective at changing behaviour. In this intervention the participants received “mindful eating training” and the hospital implemented an online pre-ordering system for staff lunch. The pre-ordering system had nutritional information displayed by all the choices, and for four of the eight week intervention period, the employees received a discount for pre-ordering rather than buying lunch direct in the canteen. Other elements included a pre-ordering system which defaulted to the healthiest choice (requiring additional effort to select less healthy options). This study had low risk of bias and was effective at improving nutritional content of purchased lunches.

In one study, in which the intervention had an effect on dietary consumption, choice architecture elements were chosen and implemented according to what suited the local context with examples relating to availability (which also changed behaviour in two other studies) and prompting (which is not studied in isolation in any other study) [59]

Methodological Quality of Included Studies

The risk of bias of included studies is presented in Tables 5 and 6. High risk of bias was seen in two of the six RCTs. Racette et al, (2009) [35] although using randomisation, only included two worksites, with quite different employee characteristics which may have introduced other sources of bias. Sorensen et al, 1999 [36 ,39-41] collected more dietary data than was presented in the report, without clear justification for why specific outcomes were reported. Nearly all of the included randomised studies did not provide details on the process of randomisation (selection bias). Two studies [37 ,42 ,45] were at low risk of performance bias where blinding was not possible but unlikely to affect objective outcomes.

In the non-randomised studies, high risk of bias in more than one domain was seen in four [26 ,34 ,38 ,43 ,44] of the twelve included studies. One study [29] had errors in the numerical data presented which was cause for concern (number of entrees sold and mean number of entrees sold/day). Only two studies were judged at low risk of bias across all domains [25 ,31]. Half of the studies were at low risk of bias for confounding variables.

For peer review only - http://bmjopen.bmj.com/site/about/guidelines.xhtml

Discussion

Statement of principal findings

Choice architecture interventions involving a proximity element appear to be frequently effective at changing behaviour. This was found in some studies that used proximity interventions alone or as part of choice architecture or complex interventions. Interventions including an availability element were generally reported to be successful at changing behaviour. However, none of the studies examined availability alone. A sizing intervention was effective in the only study identified examining this type of intervention.

There was no strong evidence for the effect of pricing on behavioural change in our review. No additional effect of pricing in a sizing and pricing intervention compared with sizing alone, and one complex intervention including pricing which was reported to be effective, but had a high risk of bias. Labelling alone was generally not effective at changing behaviour, although it was a popular addition to other interventions of which most were successful.

Strengths and weaknesses of the study

Because of the relatively small number of papers that we found during a scoping search, we made the decision to broaden the inclusion criteria in two ways. Firstly, we have included studies in which complex interventions with non-choice architecture elements were implemented and it is not always possible to draw conclusions about the contribution of the choice architecture elements to the outcomes. Secondly, we have included studies in which not all the workplaces were healthcare settings. We did this because this gives us some evidence of interventions which are feasible in healthcare settings, however because data was not disaggregated, we cannot be sure that outcomes were similar across the different sorts of workplaces included (ie: an element that was effective overall, may have been less effective or not effective in the health-care settings; or an element that was not effective overall may have been effective in the health-care settings). We conducted a comprehensive search across major electronic databases relevant to the topic of this review. Each included study in this review was comprehensively selected, assessed, data extracted, and quality assessed by two review authors independently to minimise potential biases in the review process. Where data of relevance were missing, either to allow assessment of eligibility, or at the data extraction stage and assessment of bias, the review authors (OO, LA, OU) contacted the study authors for further information.

Strengths and weaknesses in relation to other studies, discussing important differences in results

When Hollands et al (2013) [20] appraised the choice architecture literature in mid-2011, for the purposes of developing the typology we have used in this review, they noted that 40% of all study reports examined interventions which involved point-of-choice labelling and/or prompting elements [20]. We found labelling and proximity elements were the most popular type of choice architecture employed in our included studies, both alone or in combination with other intervention elements. The difference might be due to the scope of the literature examined, we were restricted to those aiming to change dietary behaviour while Hollands et al (2013) looked more broadly at a range of behaviours.

Our study is specifically focused on employees of health care settings, a unique population who are likely to be more health conscious than the general population. For this reason, our findings are not necessarily generalizable outside these settings and other's findings may not be relevant for health care staff.

For peer review only - http://bmjopen.bmj.com/site/about/guidelines.xhtml

The finding of this review found mixed results for labelling interventions suggesting that they are unlikely to be effective in isolation in these settings. A recent systematic review evaluating the effect of restaurant menu calorie labelling found 19 studies. Meta-analysis of these studies demonstrated an 18 calorie reduction in meals ordered when all studies were combined. However, when just the six controlled studies were examined, labelling was no longer associated with a significant difference in calories ordered [60]. Another systematic review found that menu labelling with calorie information alone did not reduce the number of calories selected or consumed, although the addition of information to assist consumers in applying nutritional information was effective [61]. In a systematic review of randomised controlled trials that included food labels and excluded menu labelling, nine studies were identified and meta-analysis concluded that food labelling was effective at changing dietary behaviour although not at reducing calorie consumption [62]. Menu energy labelling across socio-economic groups was evaluated in a recent review [63]. The findings suggested that any positive benefit of this kind of labelling may only apply to higher socio-economic groups [63]. The findings of this review in the context of previous literature suggests that labelling alone should not be recommended for implementation to improve healthy food and drink purchasing of health care staff.

A large systematic review evaluated supermarket and grocery store interventions to increase consumption of healthy. Analysis of forty two studies found that consumers responded to economic incentives including discounts, vouchers and subsidies [64]. Similarly, a systematic review of interventions at point-of-sale to encourage healthier food purchasing found evidence for monetary incentives as effective at changing behaviour, but that there was no strong evidence for the other intervention types they examined [65]. We found mixed evidence on pricing in health care settings in this review.

A systematic review of interventions carried out in restaurant settings to promote healthy eating stated that the most effective interventions were point-of-purchase information with increased availability of healthy choices [66]. We also found that increasing availability of healthy options alongside other intervention elements was a successful strategy for driving behaviour change in health care settings.

Meaning of the study: possible explanations and implications for clinicians and policymakers

On the basis of this systematic review, we were able to recommend some general principles that are likely to be effective when designing choice architecture interventions for NHS organisations to Public Health England. These are "proximity" interventions in which behavioural options are made easier or harder to engage with, requiring reduced or increased effort and "availability" interventions in which healthy behavioural options are added within a given micro-environment. In addition, we are able to suggest "sizing" interventions in which the size or quantity of the product on offer is changed based on one effective study with low risk of bias. Finally, we can recommend specific examples of choice architecture interventions that have been effective in studies with low risk of bias. These recommendations are made in a companion format (please see supplementary material 3), designed for the audience who make decisions about catering in NHS organisations.

Other outcomes relevant to decision-makers

We examined primary outcomes relating to dietary consumption and purchasing of food and drink. However, outcomes that are important to decision-makers include customer satisfaction and profitability. One of our included studies which examined various arrangements and assortments of snacks on offer at the checkout counter in a hospital staff restaurant found in a survey of 92 participants that the more visible the healthy snacks were in terms of location (ie: on top shelves) and proportion (ie: 75% healthy vs 25% healthy) the more attractive the entire selection was

For peer review only - http://bmjopen.bmj.com/site/about/guidelines.xhtml

perceived to be [31]. Another included study noted that the presentation of caloric values of the food significantly decreased the total number of calories bought without significantly affecting the total money spent [28].

In a study of nine early adopters of the Hospital Healthier Food Initiative and the Health and Sustainability Guidelines in the USA, the main barriers to implementation were reported to be customer complaints, a shortage of food and drinks that met the requirements of the Initiative/Guidelines and concern over profitability. However, most respondents had been able to overcome these barriers and it was noted that customer volumes and sales were reported to have increased subsequent to implementation which offset initial investments necessary to successfully meet the requirements of the Initiative/Guidelines [16].

A study in which a "healthful food station" was introduced to a worksite cafeteria in an academic medical centre in the USA, found that some customers reported that they used the cafeteria more often due to the presence of the station. Others had only started using the cafeteria because the station had been introduced. This study also found that the healthful food station generated gross profit [56].

Unanswered questions and future research

Reporting of studies was generally poor. For instance both randomised and non-randomised studies did not generally report on domains that may lead to selection bias. We are generally unsure whether subjective outcomes were collected by blinded assessors which may lead to detection bias. Overall, the methodological quality of included studies was low and future research should clearly report on this. Therefore the results of this review should be interpreted with cautious.

In health care settings, increasing availability of healthy options alongside other intervention elements was found to be successful in improving behaviour (specifically increasing consumption of fruit and vegetables). However, we do not know the effect of increasing availability alone.

We could not come to a clear conclusion whether a pricing element is clearly successful in improving healthy food and drink purchasing and consumption in health care settings.

No studies investigated the effect of ambience or priming elements on modifying dietary behaviour in health care settings.

Declaration of competing interests

All authors have completed the ICMJE uniform disclosure form at www.icmje.org/coi_disclosure.pdf and declare: no support from any organisation for the submitted work; no financial relationships with any organisations that might have an interest in the submitted work in the previous three years; no other relationships or activities that could appear to have influenced the submitted work.

Details of contributors

LA and OO designed the protocol; LA, OO and SJ designed and ran the searches; LA and OO performed the initial screening; LA, OO and OU screened full text articles; LA, OO, OU and RW extracted data from included studies; LA and OO synthesized extracted data and wrote the first draft of the manuscript. All authors contributed to the final submitted manuscript.

Acknowledgement

The authors would like to thank Aileen Clarke, Warwick Medical School; Ruth Breese, South Warwickshire NHS Foundation Trust; Elizabeth Atherton, Food for Life Programme, Soil Association;

For peer review only

Laura Brown, Sarah Golding and Tim Chadborn, Public Health England, all of whom gave comments and feedback that have supported this work.

Ethics approval

Not required

Funding Sources

This piece of research was funded by Public Health England Behavioural Insights Team who gave feedback on the study protocol. The funder had no role in the collection, analysis or interpretation of data, in the writing of the report or decision to submit the article for publication. LA and OO are supported by the National Institute of Health Research (NIHR) Collaboration for Applied Health Research and Care West Midlands initiative. The views expressed are those of the authors and not necessarily those of the NHS, the NIHR or the Department of Health. RW was supported by University of Warwick through its Global Research Priority on Food. OU is supported by the National Institute of Health Research using Official Development Assistance (ODA) funding. SJ is supported by the University of Warwick.

Transparency Declaration

The lead author affirms (as the manuscript's guarantor) that the manuscript is an honest, accurate, and transparent account of the study being reported; that no aspects of the study have been omitted; and that any discrepancies from the study as registered have been explained.

Data sharing statement

No additional data available.

What this paper adds

What is already known on this subject

- Changing the environment to promote healthy behaviour can be a low cost effective strategy.
- Proximity and prompting are choice architecture elements that are commonly used in work place settings to improve behavior of employees.

What this study adds

- Proximity, availability and sizing are elements that are likely to be effective when designing choice architecture interventions for NHS organisations.
- Labelling is a popular addition to other interventions in choice architectural literature. Labelling alone is not recommended for implementation to improve healthy food and drink purchasing of health care staff.

Figure 1: PRISMA flow diagram

For peer review only - http://bmjopen.bmj.com/site/about/guidelines.xhtml

Table 2: Characteristics of included studies

Study	Participants/worksite	Interventions	Outcomes
**Dorresteijn (2013)**[67]	Canteen, Medical Centre.<br>University Medical Centre with over 11 000 employees, over 1000 hospital beds and over 2000 customers visiting the hospital restaurant each day.<br>***Inclusion/exclusion:*** N/A (data not collected from individuals).	(1) Point-of-decision prompts on hospital elevator doors promoting stair use. In the hospital restaurant: (2) Point of- purchase prompts promoting reduced-salt soup.<br>(3) Point-of-purchase prompts promoting lean croissants.<br>(4) Reversal of the accessibility and availability of diet margarine and butter.<br>***Comparator:*** No comparator	Number and ratio of purchased normal-salt soup, reduced-salt soup, croissants, lean croissants purchased, diet margarine and butter.
**Geaney (2011)** [68]	Canteen, hospital.<br>Participants were representative sample of 100 adults aged 18–64 years working in two public sector hospitals (fifty staff from each hospital).<br>***Inclusion:*** Being an employee and consuming at least one main meal in the hospital staff canteen daily.<br>***Exclusion:*** None reported.	Restricting food high in salt, fat and sugar. Modifying menus to make healthier options available. Replacing purchasing orders for high salt products (e.g. bacon) with low salt alternatives (e.g. turkey). Salt removed in all cooking places and tables but available in small sachets at service. Nutrition information on salt reduction and a healthy diet displayed. No sauces or accompaniments added without customer's consent. Staff encouraged to consume extra salad and vegetable options at no extra charge. Cooking oil use limited. All desserts fruit based.<br>*Comparator:* canteen at another hospital with no intervention.	Macronutrients (g/d): total sugars, sucrose, fructose, total fat, saturated fat, protein, carbohydrates, salt.<br>Micronutrients (mg/d): K, Ca, Fe, Vitamin B6, Vitamin B12, Vitamin C, Vitamin D.
**Lassen (2014)**[69]	Canteen, hospital.<br>The canteen was seeking Keyhole certification in Denmark. Participants were canteen customers approached at random after purchasing lunch.<br>***Inclusion:*** Purchasing lunch in the	Introduction of the Keyhole symbol on freshly prepared food in the canteen. The Keyhole symbol is used in the Nordic countries as a sign of a healthy choice (meeting certain criteria).<br>***Comparator:*** no intervention.	Energy per meal consumed (kJ), energy density of meal (kJ/100g), fat content (E%), fruit and vegetables (g/100g), salt (g/100g), refined sugars (g/100g), wholegrain (g/100g)

For peer review only - http://bmjopen.bmj.com/site/about/guidelines.xhtml

	canteen.<br>***Exclusion:*** Not reported.		
**Lowe (2010)** [34]	Canteens, hospitals.<br>***Inclusion:*** Reporting eating lunch in the hospital cafeteria at least two times each week, on average, being between 21 and 65 years of age and being a hospital or university employee.<br>***Exclusion:*** Having a current diagnosis of a chronic disease or condition known to affect appetite or body weight, taking medication known to affect appetite or body weight, being pregnant or planning to become pregnant within the next 24 months, being enrolled or having plans to 15 label within the next 24 months in an organized weight management program, and/or having plans to terminate hospital employment within the next 12 months.	When the intervention period began, participants in both groups were exposed to environmental change (EC): reductions in the energy density (ED) of some foods offered in the cafeteria and introduction of nutritional labels for all foods sold in the cafeterias. Participants in the EC-Plus condition received two additional intervention components: training in reducing the ED of their diet and discounts on low-ED foods purchased in the cafeteria.<br>***Comparator:*** environmental change only.	Fruit (servings/ day) , vegetables (servings/day) , bread products (servings/day) , dairy products (servings/day) , fats and sweets (servings/day) , meats (servings/day); Purchased kcal (kcal): purchased calories from fat (%); purchased calories from protein (%); purchased calories from carbohydrate (%)
**MacDonald (2016)** [38]	Aboriginal Community controlled health organisations.<br>**Inclusion/exclusion:** not reported.	A tailored 'Healthy Catering Toolkits' to local caterers at each site and included order forms classified according to the 'traffic light guide' to ensure healthy catering choices were easy ordering choices for staff. They also distributed 'traffic light guide' posters and information sheets, a nutrition policy template and a wallet-sized card for interpreting food labels.<br>**Comparator:** no comparator.	Proportion of purchased foods in category 'foods to limit' (%)
**Meyers (1980)** [27]	Canteen, Hospital.<br>A hospital cafeteria that served hospital staff, students and hospital visitors.<br>**Inclusion/exclusion:** not reported.	Desserts were arrayed in columns four deep along the cafeteria counter. On control days research assistants arrayed desserts in columns of four, high calorie	Dessert purchases.

For peer review only

		desserts alternating with low-calorie desserts, permitting equal access to each dessert. High-calorie desserts were placed in the two front positions with low calorie desserts in the read (less accessible) positions on days 3 and 5. On days 2 and 4 this order was reversed.<br>**Comparator:** No comparator.	
**Milich (1976)** [28]	Canteen, hospital.<br>**Inclusion:** Being a female employee (identifiable by an ID badge and/or uniform) and having a food tray.<br>**Exclusion:** Being a patient or visitor, being visibly pregnant or being an employee who had brought food from home or only bought part of her meal in the cafeteria.	Calorie signs were posted for foods in the cafeteria.<br>The intervention consisted of 1 week of Price Increase, followed by 1 week of Price Increase and Calorie Presentation<br>**Comparator:** No comparator.	Calories bought (kcal), money spent ($).
**Racette (2009)** [35]	Worksites, medical centre.<br>**Inclusion:** All employees over 18 years of age were eligible, including those who smoked, had pre-existing disease (e.g. hypertension, diabetes), or used medications.<br>**Exclusion:** not reported.	The intervention was comprised of a constellation of nutrition components, physical activity components, and incentives designed to promote healthy dietary and physical activity behaviours, with the goals of promoting weight control and reducing risk factors for cardiovascular disease.<br>Specific intervention components included weekly healthy snack cart, monthly lunchtime seminars, monthly newsletters, walking maps, participation cards and participation rewards. Each week a registered dietitian/exercise specialist was available at the worksite to provide support.<br>**Comparator:** Health assessments only.	Fruit and vegetable intake (servings/day).

For peer review only

**Sato (2013)** [29]	Cafeteria, Medical Centre. Customers of the medical centre cafeteria aged 18 years and older. Roughly 70% of the customers at Kaiser Permanente San Francisco Medical Center are Kaiser employees; the rest are visitors or patients in the hospital. The cafeteria serves roughly 100 customers per day. **Inclusion/exclusion:** N/A (receipts collected anonymously).	A 'Healthy Pick' meeting various criteria was made available every day. The meals available were labelled with a label featuring calories, fat and sodium. **Comparator:** No comparator.	Number of Healthy Picks purchased, number of main meals purchased.
**Sorensen (1999)** [36 ,39-41]	Community health centres. **Inclusion:** Being a permanent employee and working at least 15 hours a week. **Exclusion:** not reported.	The core intervention included periodic exposure to national 5-a-Day campaigns and a general nutrition presentation. In the additional worksite intervention, educational activities and changes were made to the worksite environment including labelling and adding fruit and vegetables to vending machines. **Comparator:** core intervention only.	Fruit and vegetable intake (servings/ day).
**Stites (2015)** [37]	Canteen, hospital. **Inclusion:** Having a BMI of at least 25.0 kg/m^2, eating at least three lunches/week in the hospital cafeteria, being willing to allow researchers to collect data about their lunch purchases, and having access to a computer at work. **Exclusion:** Having a diagnosis of unstable hypertension, dyslipidemia, or coronary artery disease whose medical therapy had changed in the past three months, having plans to terminate hospital employment within four months following study enrolment, or being pregnant.	During the Full Intervention phase participants received mindful eating training, were encouraged to pre-order their lunches, and were given vouchers to use on lunch purchases. Following this there was a Partial Intervention phase, where participants were encouraged to pre-order their lunches but did not receive vouchers. The pre-ordering system listed the food available daily in the cafeteria, along with nutritional information. Employees had to order at least 45 minutes ahead of scheduled pick up time. **Comparator:** wait list control.	Kcal per lunch purchased (kcal), fat grams per lunch purchased (g).

For peer review only - http://bmjopen.bmj.com/site/about/guidelines.xhtml

**Thorndike (2014)** [30 ,32 ,33]	Canteen, hospital.<br>Hospital employees have the option of paying for cafeteria purchases by direct payroll deduction using a “platinum plate” card.<br>**Inclusion:** Using “platinum plate” and making a purchase in the cafeteria at least three times during each 3-month period from baseline to the end of follow up.<br>**Exclusion:** Not reported.	A traffic light food labelling system was introduced, along with signage explaining it. Three months later further changes were introduced, including rearranging items in the beverage and sandwich refrigerators to put all the green items at eye level; placing baskets of bottled water throughout the cafeteria; and providing pre-packaged salads next to the pizza counter.<br>**Comparator:** No comparator.	Of items bought: % red, % green (red labels and green labels as denoting unhealthy and healthy choices respectively).<br>Of beverages bought: % red , % green.
**Van Kleef (2012)** [31]	Canteen, Hospital.<br>**Inclusion/exclusion:** N/A	Four successive weeks were randomly assigned to four experimental conditions:<br>Intervention 1: 25% of products healthy, these located at top shelves.<br>Intervention 2: 25% of products healthy, these located at bottom shelves.<br>Intervention 3: 75% of products healthy, these located at bottom shelves.<br>Intervention 4: 75% of products healthy, these located top at shelves.<br>**Comparator:** no comparator.	Number of healthy snacks sold, number of unhealthy snacks sold.
**Beresford (2001)** [46]	28 worksites (6 health service organisations).<br>Inclusion: (For worksites) Having a food-serving cafeteria and having between 250 and 2000 employees.<br>**Exclusion:** N/A	The intervention was tailored to worksites. Elements included a kick-off event, the 5-a-Day message being posted on boards in each worksite, more fruit and vegetables becoming part of the menus and the provision of a self-help manual for every employee.<br>**Comparator:** no intervention.	Fruit and vegetable intake (servings/ day).
**Holdsworth (2004)** [43 ,44]	6 worksites (two health care).<br>**Inclusion/exclusion:** not reported.	The Heartbeat Award scheme increases opportunities for behaviour change by providing customers with information,	Daily intake of: biscuits and cakes, sweet puddings, confectionery, sugary drinks, processed meat, hard cheese,

For peer review only - http://bmjopen.bmj.com/site/about/guidelines.xhtml

		reminders and reinforcement to guide them towards healthier choices. **Comparator:** no intervention.	crisps, low-fat cheese, fried food, beans and pulses, fruit, vegetables, chicken and fish, starchy foods, red meat, low calorie drinks. All reported: (1) no change/negative change; (2) positive change.
**Kwak (2009)** [48 ,59]	12 worksites (two hospitals). **Inclusion:** Employees with BMI > 18kg/m2 and not under any medical restrictions. **Exclusion:** Not reported.	There was an individual and an environmental component to the intervention. Interventions included changes in the assortment of food products in the cafeteria, workshops, an information wall containing information on the balance between food intake and physical activity, posters or prompts stimulating stair use, and ways to form lunch-walking and cycling groups.	Intake of: Fibre rich bread, low fibre bread, fibre-rich main meal, low energy dense toppings, high energy dense toppings, low energy dense main meal, high energy dense main meal, low energy dense snacks, high energy dense snacks, low energy dense drinks, high energy dense drinks (all in servings/day).
**Sorensen (1992)** [47 ,49]	16 worksites (at least one intervention and one control site specialising in "health care"). **Inclusion:** (for worksites) Having 200-2000 employees, having a cafeteria with a kitchen, having an annual turnover rate of <25%, having <25% of employees working rotating shifts, part time or off site and being stable as a company, defined as having no plans for geographic relocation or major layoffs in the next 2 years. **Exclusion:** not reported.	Worksites received a 15-month intervention with various elements. These included direct education programs, such as classes offered in all intervention worksites and optional activities tailored to individual worksites, and environmental programs which targeted cafeterias both to increase the availability of low-fat, high-fibre foods and to provide supportive nutrition education by labelling those food options. **Comparator:** no intervention.	Total dietary fat (% kcal), total dietary fibre (g)
**Vermeer (2011)** [42 ,45]	25 worksites (15 hospitals). **Inclusion:** (for worksites) Selling on average 30 meals/day, offering a reducible meal at least twice per week, being willing to be randomly allocated to a study condition, following the	Intervention 1: a smaller portion (that is, about two-thirds of the size of the existing portion) was offered in addition to the existing portion and proportional pricing was employed (that is, the price was 65% of the existing size).	Number of large meals sold, number of small meals sold, number of fried snacks sold.

For peer review only - http://bmjopen.bmj.com/site/about/guidelines.xhtml

	study protocol and providing their daily sales figures of hot meals and fried snacks.<br>**Exclusion:** not reported.	Intervention 2: a smaller portion was added to the assortment and value size pricing (that is, a lower price per unit for large portions than for small portions) was employed (that is, the price was 80% of the existing size).<br>**Comparator:** no intervention.	

N/A: not applicable

For peer review only

For peer review only - http://bmjopen.bmj.com/site/about/guidelines.xhtml

Table 3: Choice architecture elements and effectiveness of interventions examined in included studies

Intervention Class	Intervention Type	Studies: Dorresteijn 2013 a	Dorresteijn 2013 b	Dorresteijn 2013 c	Geaney 2011	Holdsworth 2004	Lassen 2014	Meyers 1980	Milich 1976	Sato 2013	Thorndike 2014	Van Kleef 2012	Vermeer 2011 a	Vermeer 2011 b
**Primarily alter properties of objects or stimuli**	**Ambience**													
	**Functional design**				X									
	**Labelling**	X	X		X	X	X		X	X	X			
	**Presentation**				X									
	**Sizing**												X	X
	**Pricing**													X
**Primarily alter placement of objects or stimuli**	**Availability**	X				X	X					X		
	**Proximity**			X	X			X			X	X		
**Alter both properties and placement of objects and stimuli**	**Priming**													
	**Prompting**	X									X			
**Outcome** (N= not effective; Y= effective)	**Effect on purchasing?**	N	N	Y				Y	Y	N	Y	Y	Y	Y*
**Outcome** (N=not effective; Y= effective)	**Effect on dietary consumption?**				Y	Y	Y							

X blue box: element was implemented in the study; *although sizing plus pricing had an effect compared with control, there was no significant effect between sizing and pricing and sizing alone in this study.

For peer review only - http://bmjopen.bmj.com/site/about/guidelines.xhtml

Table 4: Choice architecture elements included in complex interventions and their reported effectiveness in included studies

		**Studies**							
**Intervention Class**	**Intervention Type**	**Berefsord 2001**	**Kwak 2009**	**Lowe 2010**	**Macdonald 2016**	**Racette 2009**	**Sorensen 1992**	**Sorensen 1999**	**Stites 2015**
**Primarily alter properties of objects or stimuli**	**Ambience**								
	**Functional design**								**X**
	**Labelling**			**X**	**X**		**X**	**X**	**X**
	**Presentation**								
	**Sizing**								
	**Pricing**			**X**					
**Primarily alter placement of objects or stimuli**	**Availability**	**X**	**X***	**X**		**X**	**X**		
	**Proximity**							**X**	**X**
**Alter both properties and placement of objects and stimuli**	**Priming**								
	**Prompting**		**X***		**X**		**X**		
**Outcome** (N= not effective; Y= effective)	**Effect on purchasing?**				**Y**				**Y**
	**Effect on dietary consumption?**	**Y**	**Y**	**Y**		**Y**	**Y**	**N**	

X blue box: element was implemented in the study; *****Kwak et al, (2009) used an assets-based approach in which intervention worksites chose intervention elements that suited their context. Examples of actions given were classified as “availability” and “prompting”.

For peer review only

Table 5: Risk of bias in randomised controlled trials

	Selection bias		Performance bias	Detection bias	Attrition bias		
**Study**	random sequence generation	allocation concealment	blinding of participants + personnel (subjective/ objective outcomes)	blinding of outcome assessment (subjective/ objective outcomes)	incomplete outcome data (subjective/ objective outcomes)	**Selective outcome reporting**	**Other bias**
**Beresford 2001**	No details	No details	Blinding not possible	No details. Lack of blinding may affect subjective outcomes	Numbers provided but reasons not fully explained	Outcomes reported as in method. No protocol.	No other bias
**Racette 2009**	No details	No details	Blinding not possible	No details. Lack of blinding may affect subjective outcomes	Numbers and reasons provided	No protocol	Randomisation was only over two sites. Demographical difference between groups.
**Sorensen 1992**	No details	No details	Blinding not possible	No details	Numbers provided and gives some reason for questionnaires being eliminated from analyses	FFQ was used but only fibre and fat reported.	No other bias
**Sorensen 1999**	No details	No details	Blinding not possible	No details	Not reported but analysis presented for all sample (not restricted for completers).	"*Additional dietary data, not presented here, were collected by means of the Food Frequency Questionnair*"	No significant differences among the groups at baseline. All analyses were computed by taking into consideration the nesting of employees in worksites.
**Stites 2015**	Using a computer program	No details	Blinding not possible	No details	Mainly reported and reasons explained,	Outcomes reported as in	No other bias

For peer review only - http://bmjopen.bmj.com/site/about/guidelines.xhtml

					for one participant, no reason explained.	method. No protocol.	
**Vermeer 2011**	No details	No details	Blinding not possible	No details	Numbers provided but reasons not fully explained	Outcomes reported as in method. No protocol.	No other bias

Orange: unclear risk of bias; Green: low risk of bias; Red: high risk of bias; "*quote form publication*"

For peer review only

For peer review only - http://bmjopen.bmj.com/site/about/guidelines.xhtml

Table 6: Risk of bias is non-randomised studies

	**Selection bias**		**Performance bias**	**Detection bias**	**Attrition bias**	
**Study**	selection of participants	confounding variables	Measurement of exposure	blinding of outcome assessment (subjective/ objective outcomes)	incomplete outcome data (subjective/ objective outcomes)	**Selective outcome reporting**
**Dorresteijn 2013**	Data were collected prospectively	Confounding variables (weather) were considered	All were exposed, performance bias is not affected by exposure	Blinding not possible and unlikely to affect objective outcomes	Characteristics of study before and after the intervention are available	All of the expected outcomes were included in the study descriptions
**Geaney 2011**	*"A random sample of 100 individuals took part in the study (fifty staff from each hospital)."* *"In the intervention hospital, all individuals who were asked to participate agreed. Less than five in the non-intervention hospital refused to participate in the study."*	Confounding variables (age and gender) were considered	All were exposed, performance bias is not affected by exposure	No details	Cross-sectional there was no attrition	All of the expected outcomes were included in the study descriptions
**Holdsworth 2004**	*" Two premises applying for the HBA were*	Confounding variables (adjusted for age, gender,	All were exposed, performance bias is not affected by	Lack of blinding may affect subjective outcomes	Numbers provided (low response rate)	All of the expected outcomes were included in the study

For peer review only - http://bmjopen.bmj.com/site/about/guidelines.xhtml

	unsuccessful in receiving it, and were used as a comparison group for the study"	ethnicity, social class and BMI) were considered	exposure			descriptions
**Kwak 2009**	*"A sample of 128 worksites meeting the inclusion criteria were selected through the Chamber of Commerce and invited by letter and telephone to take part in this study....12 worksites agreed to participate"*	Confounding variables (matched based on the social economic status of their employees) were considered	All were exposed, performance bias is not affected by exposure	Lack of blinding may affect subjective outcomes	*" At baseline, the average response rate to the questionnaire was 88.1% (487 of 553). At the first follow-up, the response rate average was 82.5% (n=376) and at the second follow-up 75.8% (n=303). During the course of the project, one of the worksites in the intervention group underwent reorganization; half of the employees were made redundant, thereby causing most of the dropout in our study. There was no selective dropout at the first follow-up. At the second follow-up, smokers were more likely to discontinue the study than non-smokers"*	More items collected than reported
**Lassen 2014**	*"The employees*	Confounding	All were exposed,	Lack of blinding	Evidence significant	*"For the outcomes of*

For peer review only - http://bmjopen.bmj.com/site/about/guidelines.xhtml

	were randomly approached after having bought their meal and asked to participate in the study focusing on canteen food intake".	variables (demographics, seasonal change) were not considered	performance bias is not affected by exposure	unlikely to affect objective outcomes.	differential in number of health care personnel between baseline & follow-up	*refined sugar, wholegrain and plate waste a large number of participants had zero intake. These were analysed in two steps. First the probability of any intake yes/no was analysed…. then a regression model was fitted as described above with regard to the participants with a positive intake"*
**Lowe 2010**	*"Participants were recruited through letters distributed to cafeteria patrons that provided an overview of the study along with inclusion and exclusion criteria"*	No details	All were exposed, performance bias is not affected by exposure	Lack of blinding is unlikely to affect objective outcomes.<br>For subjective outcomes *"Dietary recalls were conducted by the Diet Assessment Center at The Pennsylvania State University"*	Numbers provided (high attrition rate). *"Data from participants who did not scan their cards an average of at least four times per month were excluded from analyses"*	Reporting of data was not always per intervention group with no justification
**Macdonald 2016**	5 centres were chosen to participate including a range of contexts (metropolitan, rural, regional)	Confounding variables (changes at the sites) were not considered	All were exposed, performance bias is not affected by exposure	*"catering receipts received included generic food types such as sandwiches. Categorisation into food types in these instances were*	*"two ACCHOs were able to provide catering receipts at baseline and follow up"*	All of the expected outcomes were included in the study descriptions

For peer review only - http://bmjopen.bmj.com/site/about/guidelines.xhtml

				based on inferences of the project dietitian"<br><br>*"the number of serves provided where invoices listed general categories; for example, 'large fruit platter' were made from inferences based on cost by the project dietitian"*		
**Meyers 1980**	Data were collected prospectively	No details	All were exposed, performance bias is not affected by exposure	*"observations were carried out by undergraduate research assistants"*	N/A	All of the expected outcomes were included in the study descriptions
**Milich 1976**	*"From original total pool of sampled employees 50 subjects were randomly sampled for each body size in each experimental condition"*	Confounding variables (weather, obesity and price change) were considered	All were exposed, performance bias is not affected by exposure	Outcome assessed by assessing food items on plate then calculating caloric value	N/A	All of the expected outcomes were included in the study descriptions
**Sato 2013**	Data were prospectively collected	Confounding variables (price change) were not considered	All were exposed, performance bias is not affected by exposure	Lack of blinding is unlikely to affect objective outcomes.	N/A	All of the expected outcomes were included in the study descriptions
**Thorndike 2014**	*"employee platinum plate users who made a*	Confounding variables (age, gender, race, job	All were exposed, performance bias is not affected by	Lack of blinding is unlikely to affect objective outcomes.	*"1.7% of employees missing any sociodemographic data*	All of the expected outcomes were included in the study

	purchase in the cafeteria at least three times during each 3-month period from baseline to the end of follow up were included in the study cohort"	type, and work status) were considered	exposure		*were excluded from the analysis"* Information about the number of participants before and after the study exists, however at each time point the cohort was not identical	descriptions
**Van Kleef 2012**	*"carried out in hospital staff restaurant and visitors are allowed to purchase displayed snacks at a self-service checkout counter throughout the day over a four week period. Data were collected prospectively"*	No details	All were exposed, performance bias is not affected by exposure	Lack of blinding is unlikely to affect objective outcomes.	N/A	All of the expected outcomes were included in the study descriptions

Orange: unclear risk of bias; Green: low risk of bias; Red: high risk of bias; Grey: not applicable; N/A: not applicable; "*quote form publication*"

For peer review only - http://bmjopen.bmj.com/site/about/guidelines.xhtml

References

1. Newton JN, Briggs AD, Murray CJ, et al. Changes in health in England, with analysis by English regions and areas of deprivation, 1990–2013: a systematic analysis for the Global Burden of Disease Study 2013. The Lancet 2015;386:2257-74.
2. Ng M, Fleming T, Robinson M, et al. Global, regional, and national prevalence of overweight and obesity in children and adults during 1980–2013: a systematic analysis for the Global Burden of Disease Study 2013. The Lancet 2014;384:766-81.
3. Scarborough P, Bhatnagar P, Wickramasinghe KK, et al. The economic burden of ill health due to diet, physical inactivity, smoking, alcohol and obesity in the UK: an update to 2006–07 NHS costs. J Public Health 2011;33:527-35.
4. NHS Digital. Secondary Health Survey for England 2015: Adult overweight and obesity 2016. https://digital.nhs.uk/catalogue/PUB20562 (accessed November 2017).
5. Butland B, Jebb S, Kopelman P, et al. Tackling obesities: future choices-project report. London: Department of Innovation, Universities and Skills 2007; 10:17.
6. Shrestha N, Pedisic Z, Neil-Sztramko S, Kukkonen-Harjula KT, Hermans V. The impact of obesity in the workplace: a review of contributing factors, consequences and potential solutions. Current obesity reports 2016;5:344-60.
7. Koepp GA, Snedden BJ, Levine JA. Workplace slip, trip and fall injuries and obesity. Ergonomics 2015;58:674-79.
8. Flint SW, Snook J. Disability discrimination and obesity: the big questions?. Current obesity reports 2015;4:504-09
9. NHS England 2015. Simon Stevens announces major drive to improve health in NHS workplace. https://www.england.nhs.uk/2015/09/nhs-workplace/ (accessed November 2017).
10. Blake H, Mo PK, Lee S, Batt ME. Health in the NHS: lifestyle behaviours of hospital employees. Perspectives in public health 2012;132:213.
11. Kyle RG, Neall RA, Atherton IM. Prevalence of overweight and obesity among nurses in Scotland: A cross-sectional study using the Scottish Health Survey. Int J Nurs Stud 2016;53:126-33
12. Blake H, Harrison C. Health behaviours and attitudes towards being role models. British Journal of Nursing 2013;22:86-94
13. Dalton M. Implementing NICE obesity guidance for staff: an NHS trust audit. Occup Med 2014;65:78-85
14. Young A. Innovation within a national health care system. Surgery 2017;161:1179-82
15. World Health Organization. Healthy Workplaces: A Global Framework and Model: Review of Literature and Practices. Geneva, 2010. www.who.int/occupational_health/healthy_workplaces/en/index.html (accessed January 2018).
16. Jilcott Pitts S, Graham J, Mojica A, et al. Implementing healthier foodservice guidelines in hospital and federal worksite cafeterias: barriers, facilitators and keys to success. J Hum Nutr Diet 2016;29:677-86
17. NHS England 2017a. Commissioning for Quality and Innovation (CQUIN) Indicator Specification 2017-2019. https://www.england.nhs.uk/publication/cquin-indicator-specification/(accessed December 2017).
18. Al-Khudairy L, Uthman O, Johnson S, Clarke A, Oyebode O. Behavioural interventions to increase healthier purchasing and/or consumption of food and drink by NHS staff. PROSPERO 2017; CRD42017064872.

For peer review only - http://bmjopen.bmj.com/site/about/guidelines.xhtml

19. Hollands GJ, Shemilt I, Marteau TM, et al. Altering micro-environments to change: towards an evidence base for choice architecture interventions. BMC public health 2013;13:1218.
20. World Bank 2017. High Income. https://data.worldbank.org/income-level/high-income (accessed November 2017).
21. Willett W. Nutritional epidemiology: Oxford University Press 2012.
22. Higgins JPT, S G. Cochrane Handbook for Systematic Reviews of Interventions Version 5.1.0. The Cochrane Collaboration 2011.
23. Kim SY, Park JE, Lee YJ, et al. Testing a tool for assessing the risk of bias for nonrandomized studies showed moderate reliability and promising validity. J Clin Epidemiol 2013;66:408-14.
24. Geaney F, Harrington J, Fitzgerald AP, Perry IJ. The impact of a workplace catering initiative on dietary intakes of salt and other nutrients: a pilot study. Public Health Nutr 2011;14:1345-49.
25. Dorresteijn JA, van der Graaf Y, Zheng K, Spiering W, Visseren FL. The daily 10 kcal expenditure deficit: a before-and-after study on low-cost interventions in the work environment. BMJ open 2013;3:e002125.
26. Lassen AD, Beck A, Leedo E, et al. Effectiveness of offering healthy labelled meals in improving the nutritional quality of lunch meals eaten in a worksite canteen. Appetite 2014;75:128-34.
27. Meyers AW, Stunkard A, Coll M. Food accessibility and food choice. Arch Gen Psychiatry 1980;37:1133-35.
28. Milich R, Anderson J, Mills M. Effects of visual presentation of caloric values on food buying by normal and obese persons. Percept Mot Skills 1976;42:155-62.
29. Sato JN, Wagle A, McProud L, Lee L. Food label effects on customer purchases in a hospital cafeteria in northern California. Journal of foodservice business research 2013;16:155-68.
30. Thorndike AN, Riis J, Sonnenberg LM, Levy DE. Traffic-light labels and choice architecture: promoting healthy food choices. Am J Prev Med 2014;46:143-49.
31. Van Kleef E, Otten K, van Trijp HC. Healthy snacks at the checkout counter: A lab and field study on the impact of shelf arrangement and assortment structure on consumer choices. BMC public health 2012;12:1072.
32. Levy DE, Riis J, Sonnenberg LM, Barraclough SJ, Thorndike AN. Food choices of minority and low-income employees: a cafeteria intervention. Am J Prev Med 2012;43:240-48
33. Thorndike AN, Sonnenberg L, Riis J, Barraclough S, Levy DE. A 2-phase labeling and choice architecture intervention to improve healthy food and beverage choices. Am J Public Health 2012;102:527-33.
34. Lowe MR, Tappe KA, Butryn ML, et al. An intervention study targeting energy and nutrient intake in worksite cafeterias. Eating behaviors 2010;11:144-51.
35. Racette SB, Deusinger SS, Inman CL, et al. Worksite Opportunities for Wellness (WOW): Effects on cardiovascular disease risk factors after 1 year. Prev Med 2009;49:108-14.
36. Sorensen G, Stoddard A, Peterson K, et al. Increasing fruit and vegetable consumption through worksites and families in the Treatwell 5-a-Day study. Am J Public Health 1999;89:54-60.
37. Stites SD, Singletary SB, Menasha A, et al. Pre-ordering lunch at work. Results of the what to eat for lunch study. Appetite 2015;84:88-97.
38. MacDonald C, Genat B, Thorpe S, Browne J. Establishing health-promoting workplaces in Aboriginal community organisations: healthy eating policies. Aust. J. Prim. Health 2016;22:239-43

For peer review only

For peer review only - http://bmjopen.bmj.com/site/about/guidelines.xhtml

39. Hunt MK, Lederman R, Potter S, Stoddard A, Sorensen G. Results of employee involvement in planning and implementing the Treatwell 5-a-Day work-site study. Health Educ Behav 2000;27:223-31.
40. Hunt MK, Lederman R, Stoddard A, et al. Process tracking results from the Treatwell 5-a-Day Worksite Study. American Journal of Health Promotion 2000;14:179-87.
41. Sorensen G, Hunt MK, Cohen N, et al. Worksite and family education for dietary change: the Treatwell 5-a-Day program. Health Educ. *Res* 1998;13:577-91.
42. Vermeer WM, Steenhuis IH, Leeuwis FH, Heymans MW, Seidell JC. Small portion sizes in worksite cafeterias: do they help consumers to reduce their food intake?. Int. J. Obes 2011;35:1200.
43. Holdsworth M, Raymond NT, Haslam C. Does the Heartbeat Award scheme in England result in change in dietary behaviour in the workplace?. Health Promot Internation 2004;19:197-204.
44. Holdsworth M, Haslam C, Raymond N. Does the heartbeat award scheme change employees' dietary attitudes and knowledge?. Appetite 2000;35:179-88.
45. Vermeer WM, Leeuwis FH, Koprulu S, et al. The process evaluation of two interventions aimed at portion size in worksite cafeterias. J Hum Nutr Diet 2012;25:180-88.
46. Beresford SA, Thompson B, Feng Z, et al. Seattle 5 a Day worksite program to increase fruit and vegetable consumption. Prev Med 2001;32:230-38.
47. Sorensen G, Morris DM, Hunt MK, et al. Work-site nutrition intervention and employees' dietary habits: the Treatwell program. Am J Public Health 1992;82:877-80.
48. Kwak L, Kremers SP, Visscher TL, van Baak MA, Brug J. Behavioral and cognitive effects of a worksite-based weight gain prevention program: the NHF-NRG in balance-project. J Occup Environ Med 2009;51:1437-46.
49. Sorensen G, Hunt MK, Morris DH, et al. Promoting healthy eating patterns in the worksite: the Treatwell intervention model. Health Educ. Res 1990;5:505-15.
50. Cremaschini M, Moretti R, Brembilla G, et al. [Assessment of the impact over one year of a workplace health promotion programme in the province of Bergamo]. Med Lav 2015;106:159-71.
51. Glasgow RE, Terborg JR, Hollis JF, Severson HH, Boles SM. TAKE HEART - RESULTS FROM THE INITIAL PHASE OF A WORK-SITE WELLNESS PROGRAM. Am J Public Health 1995;85:209-16.
52. Verweij LM, Proper KI, Weel AN, Hulshof CT, van Mechelen W. The application of an occupational health guideline reduces sedentary behaviour and increases fruit intake at work: results from an RCT. Occup Environ Med 2012;69:500-7.
53. Vyth EL, Steenhuis IH, Heymans MW, et al. Influence of placement of a nutrition logo on cafeteria menu items on lunchtime food Choices at Dutch work sites. J Am Diet Assoc 2011;111:131-6.
54. Alinia S, Lassen AD, Krogholm KS, et al. A workplace feasibility study of the effect of a minimal fruit intervention on fruit intake. Public Health Nutr 2011;14:1382-7.
55. Dawson J, Dwyer JJM, Evers S, Sheeshka J. Eat smart! Workplace Cafeteria Program - Evaluation of the nutirition component. Can J Diet Pract Res 2006;67:85-90.
56. Kimathi AN, Gregoire MB, Dowling RA, Stone MK. A healthful options food station can improve satisfaction and generate gross profit in a worksite cafeteria. J Am Diet Assoc 2009;109:914-7.
57. Van Hulst A, Barnett TA, Dery V, Cote G, Colin C. Health-promoting vending machines: evaluation of a pediatric hospital intervention. Can J Diet Pract Res 2013;74:28-34.
58. Hurd J, Mitchell H, Hawkins C, Douglas E. The get on track challenge: An effective physical activity and healthy eating program for workplaces. Obesity Reviews 2014;15:221.

For peer review only - http://bmjopen.bmj.com/site/about/guidelines.xhtml

59. Kwak L, Kremers SP, Candel MJ, et al. Changes in skinfold thickness and waist circumference after 12 and 24 months resulting from the NHF-NRG In Balance-project. Int. J. Behav. Nutr. Phys. Act 2010;7:26.
60. Long MW, Tobias DK, Cradock AL, Batchelder H, Gortmaker SL. Systematic review and meta-analysis of the impact of restaurant menu calorie labeling. Am J Public Health 2015;105:e11-e24.
61. Sinclair SE, Cooper M, Mansfield ED. The influence of menu labeling on calories selected or consumed: a systematic review and meta-analysis. J. Acad. Nutr. Diet 2014;114:1375-88. e15.
62. Cecchini M, Warin L. Impact of food labelling systems on food choices and eating behaviours: a systematic review and meta-analysis of randomized studies. Obesity reviews 2016;17:201-10.
63. Sarink D, Peeters A, Freak-Poli R, et al. The impact of menu energy labelling across socioeconomic groups: A systematic review. Appetite 2016;99:59-75.
64. Adam A, Jensen JD. What is the effectiveness of obesity related interventions at retail grocery stores and supermarkets?—a systematic review. BMC public health 2016;16:1247.
65. Liberato SC, Bailie R, Brimblecombe J. Nutrition interventions at point-of-sale to encourage healthier food purchasing: a systematic review. BMC public health 2014;14:919.
66. Espino JNV, Guerrero N, Rhoads N, et al. Peer Reviewed: Community-Based Restaurant Interventions to Promote Healthy Eating: A Systematic Review. Prev. Chronic Dis 2015;12
67. Dorresteijn JAN, Van Der Graaf Y, Zheng K, Spiering W, Visseren FLJ. The daily 10 kcal expenditure deficit: A before-and-after study on low-cost interventions in the work environment. BMJ Open 2013;3:e002125.
68. Geaney F, Harrington J, Fitzgerald A, Perry I. The impact of a workplace catering initiative on dietary intakes of salt and other nutrients: a pilot study. Public Health Nutr 2011;14:1345-9.
69. Lassen AD, Beck A, Leedo E, et al. Effectiveness of offering healthy labelled meals in improving the nutritional quality of lunch meals eaten in a worksite canteen. Appetite 2014;75:128-34.

For peer review only

For peer review only - http://bmjopen.bmj.com/site/about/guidelines.xhtml

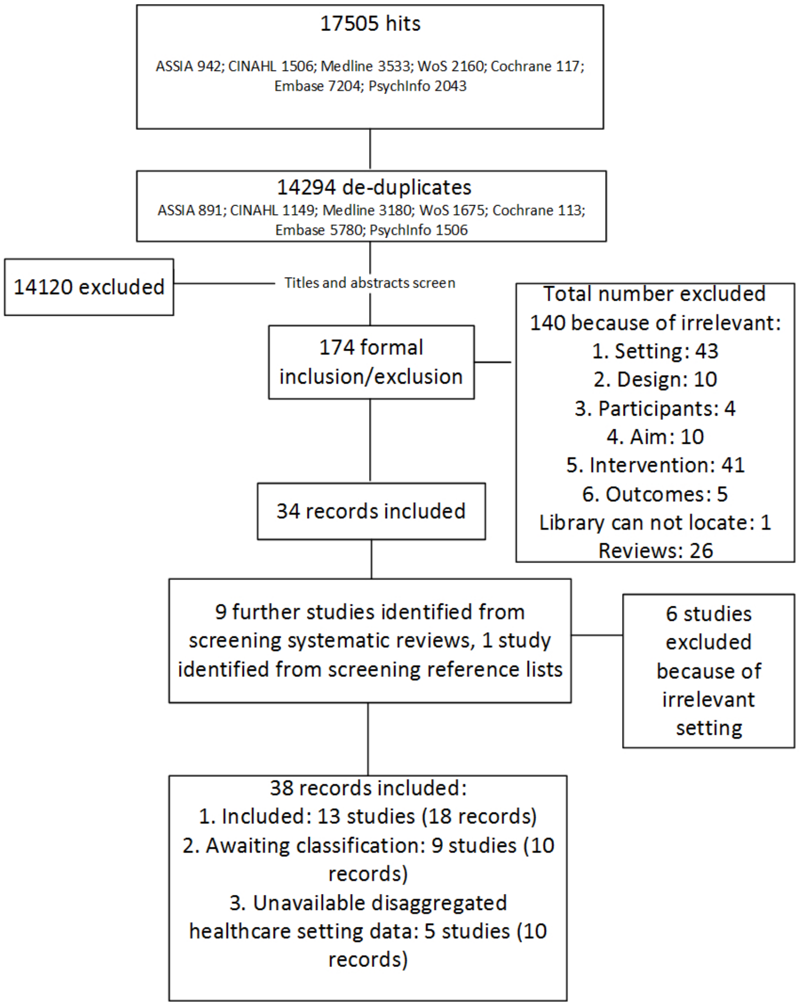

PRISMA flow diagram

90x90mm (300 x 300 DPI)

Search strategy used to interrogate MEDLINE

#	Searches	# of hits
35	limit 34 to ("all adult (19 plus years)" and humans)	2083
34	7 and 25 and 31	3734
33	limit 32 to ("all adult (19 plus years)" and humans)	4814
32	7 and 25 and 30	8677
31	26 or 27 or 28	2095764
30	26 or 27 or 28 or 29	2837423
29	health care.mp. or exp "Delivery of Health Care"/	1267298
28	exp Workplace/	17804
27	education, public health professional/ or exp "organization and administration"/	1248979
26	hospital*.mp.	1229950
25	18 or 24	537980
24	19 or 20 or 21 or 22 or 23	532969
23	eating behaviour.mp.	1262
22	Food habit*.mp.	1705
21	exp nutrition surveys/ or exp diet surveys/	21175
20	exp Nutrition Assessment/ or nutrition.mp. or exp Nutrition Surveys/	185218
19	exp Diet/ or Diet.mp.	396453
18	11 and 17	7544
17	12 or 13 or 14 or 15 or 16	1323348
16	drink*.mp.	153981
15	exp Meals/	3251
14	exp Food Preferences/	11931
13	exp beverages/ or exp food/	1173261
12	purchas*.mp.	26716
11	8 or 9 or 10	27711
10	Food Dispensers, Automatic/ or vending machine.mp.	388
9	sale*.mp.	14446
8	exp food services/ or exp food service, hospital/	13308
7	1 or 2 or 3 or 4 or 5 or 6	311674
6	choice architecture.mp.	42
5	exp Self Stimulation/ or exp Choice Behavior/ or exp Decision Making/ or nudge.mp.	173309
4	lifestyle intervention*.mp.	4193
3	exp life style/ or healthy lifestyle/	78107
2	behavio?r intervention*.mp.	528
1	exp health promotion/ or exp healthy people programs/ or weight reduction programs/	66897

For peer review only

**Which outcome?**	purchasing/dietary consumption/both *[delete as appropriate]*
**Reference (complete reference)**	
**Study ID (first author, year)**	
**Endnote No.**	
**Other reports of this trial: (Give EN No. First Author and Year)**	
**Reviewer:**	LA OO OU *[delete as appropriate]*
**Date of abstraction:**	

For studies awaiting classification skip results and quality assessment

For ongoing studies skip to the end of this form

Publication details

	[delete as appropriate]
**Language of publication**	English
**Funding source**	Commercial funding / non-commercial funding / other funding (specify)
**Publication status**	peer-reviewed journal / journal supplement / full article / other (specify)

Stated aims of study

**Quote from publication: "..."**	

**Notes**	

INFORMATION FOR CHARACTERISTICS OF INCLUDED STUDIES TABLE

	[delete as appropriate]
**Study design**	
**Participants:**	
**Brief description of participants/worksite**	
Inclusion criteria	
Exclusion criteria	
**Intervention:**	
**Number of worksites**	
**Country/authors location**	
**Setting (e.g. primary care, hospital)**	
**Type of health care setting** (such as NHS vs. non-NHS)	

For peer review only

Behavioural interventions to increase healthier purchasing and/or consumption of food and drink data abstraction form

**Description of intervention** (give details of all components)		
**Type of intervention (highlight and describe)**	Choice architecture only/choice architecture + complex intervention	
**Select intervention type**		
**Primarily alter properties or objective stimuli**	Ambiance – alter aesthetic or atmospheric aspects of the surrounding environment	
	Functional design – design or adapt equipment or function of the environment	
	Labelling – apply labelling or endorsement information to product or at point of choice	
	Presentation – alter sensory qualities or visual design of the product	
	Sizing – change size or quantity of the product	
	Pricing- change price of the product	
**Primarily alter placement or objects or stimuli**	Availability – add behavioural options within a given micro-environment	
	Proximity – make behavioural options easier (or harder) to engage with, requiring reduced (or increased) effort	
**Alter both properties and placement of objects or stimuli**	Priming – place incidental cues in the environment to influence a non-conscious behavioural response	
	Prompting – use non-personalised information to promote or raise awareness of a behaviour	
**Restaurant/canteen/vending machine/other (specify)**		
**Duration of intervention (duration of follow-up)**	Intervention:<br><br>Follow-up:	
**Comparator**	Yes/no	
**Type of comparator**	No intervention/wait list, different intervention, concomitant intervention, other ( )	
**Description of comparator**		
**Pre-intervention setup**	Yes/no	
**Description of pre-intervention setup**		
**Length of pre-intervention**		

OUTCOMES

NB: subjective measures of dietary intake **validated measures are eligible**. Any other measures list and note not data extracted.

**Tools description**	
**Dietary intake**	
**Dietary tool**	
**Purchasing data details**	

For peer review only

Baseline Characteristics

	Intervention 1	Intervention 2	Comparator 1	Comparator 2	Overall
**Sample size**					
**Staff group** (grade and/or category eg: medical vs nursing vs administrative)					
**Number of (*item*) sold**					
**Type of (*item*) sold** *[insert rows as needed]*					
**Type of (*item*) sold**					
**Type of (*item*) sold**					
**Price of (item) sold** *[insert rows as needed]*					
**Price of (item) sold** *[insert rows as needed]*					

***Purchasing data** [insert item name, add rows if required]*	**Intervention 1**	**Intervention 2**	**Comparator 1**	**Comparator 2**	***P values***
**Outcomes**					
**Sales data (%/n):** specify item					
At time point (specify)					
**Sales data (%/n):** specify item					
At time point (specify)					
**Sales data (%/n):** specify item					
At time point (specify)					
**Sales data (%/n):** specify item					
At time point (specify)					
**Sales data (%/n):** specify item					
At time point (specify)					
**Sales data (%/n):** specify item					
At time point (specify)					
**Narrative findings (please add *p* values):**					

Dietary data (do not extract total caloric intake, *insert item name, add rows if required]*

For peer review only

Behavioural interventions to increase healthier purchasing and/or consumption of food and drink data abstraction form

Outcomes	Intervention 1	Intervention 2	Comparator 1	Comparator 2
**Dietary outcomes** (specify, unit)				
Baseline	(mean, SD, N)			
At time point (specify)				
Change from baseline (specify which time point) reviewer to calculate if not reported[a]				
**Dietary outcomes** (specify, unit), mean (SD), number of participants:	(mean, SD, N)			
Baseline				
At time point (specify)				
Change from baseline (specify which time point) reviewer to calculate if not reported[a]				
**Dietary outcomes** (specify, unit), mean (SD), number of participants:	(mean, SD, N)			
Baseline				
At time point (specify)				
Change from baseline (specify which time point) reviewer to calculate if not reported[a]				
**Narrative findings (please add *p* values):**				

Notes of data to convert/calculations to be made (e.g. SEM to SD, change data, unavailable date):

Survey of study investigators providing information on included trials

**Study author contacted** <br>**[DD/MM/YY]**	
**Study author replied** <br>**[DD/MM/YY]**	

For peer review only

**Study author asked for additional information**<br><br>**[short summary]**	
**Study author provided data**<br><br>**[short summary]**	

RISK OF BIAS

**Risk of bias for RCTs**	*[delete as appropriate]*	**Support for judgement**
**Random sequence generation? (selection bias)**	Low risk of bias / High risk of bias / Unclear	Quote from publication: "..."
**Allocation concealment? (selection bias)**	Low risk of bias / High risk of bias / Unclear	Quote from publication: "..."
**Blinding of participants and personnel? (performance bias) Subjective outcomes**	Low risk of bias / High risk of bias / Unclear	Quote from publication: "..."
**Blinding of participants and personnel? (performance bias) Objective outcomes**	Low risk of bias / High risk of bias / Unclear	Quote from publication: "..."
**Blinding of outcome assessment? (detection bias) Subjective outcomes**	Low risk of bias / High risk of bias / Unclear	Quote from publication: "..."
**Blinding of outcome assessment? (detection bias) Objective outcomes**	Low risk of bias / High risk of bias / Unclear	Quote from publication: "..."
**Incomplete outcome data? (attrition bias) Subjective outcomes**	Low risk of bias / High risk of bias / Unclear	Quote from publication: "..."<br><br>Comment: no missing data / reported and reasons explained / only reported / not reported
**Incomplete outcome data? (attrition bias) Objective outcomes**	Low risk of bias / High risk of bias / Unclear	Quote from publication: "..."<br><br>Comment: no missing data / reported and reasons explained / only reported / not reported
**Selective outcome reporting?**	Low risk of bias / High risk of bias / Unclear	Comment
**Other bias?**	Low risk of bias / High risk of bias / Unclear	Comment

For peer review only

**Risk of bias for non-RCTs**	*[delete as appropriate]*	**Support for judgement**
**Selection of participants:** *Selection bias caused by the inadequate selection of participants*	Low risk of bias / High risk of bias / Unclear	Quote from publication: "..." Comment
**Confounding variables:** *Selection bias caused by the inadequate confirmation and consideration of confounding variable*	Low risk of bias / High risk of bias / Unclear	Quote from publication: "..." Comment
**Measurement of exposure:** *Performance bias caused by the inadequate measurement of exposure*	Low risk of bias / High risk of bias / Unclear	Quote from publication: "..." Comment
**Blinding of outcome assessments (subjective outcomes)** *Detection bias caused by the inadequate blinding of outcome assessments*	Low risk of bias / High risk of bias / Unclear	Quote from publication: "..." Comment
**Blinding of outcome assessments (objective outcomes)** *Detection bias caused by the inadequate blinding of outcome assessments*		
**Incomplete outcome data (subjective outcomes)** *Attrition bias caused by the inadequate handling of incomplete outcome data*	Low risk of bias / High risk of bias / Unclear	Quote from publication: "..." Comment
**Incomplete outcome data (objective outcomes)** *Attrition bias caused by the inadequate handling of incomplete outcome data*		
**Selective outcome reporting:** *Reporting bias caused by the selective reporting of outcomes*	Low risk of bias / High risk of bias / Unclear	Quote from publication: "..." Comment

Ongoing studies

For peer review only

**Study name**	Acronym:	
**Methods**	Study design:	
	Type of health care setting:	
	Country:	
**Participants**	Enrollment: (e.g. estimated 40)	
	Inclusion criteria:	
	Exclusion criteria:	
**Interventions**	Intervention(s):	
	Comparator(s):	
**Outcomes**	OUTCOME(S)	
**Starting date**	Study start date:	
	Study completion date:	
**Contact information**	Responsible party/principal investigator:	
**Study identifier** (if applicable)	(e.g.) NCT number:	
**Official title**		
**Stated purpose of study** (aim)	Quote: "..."	
**Notes**		

For peer review only

Evidence-based environmental changes to promote healthy eating and drinking by NHS staff

The size of the problem

- The leading risk behaviour for disability and death in England is poor diet[1].
- Disease associated with poor diet costs the NHS at least £5.8 billion per year[2].
- Nearly two thirds of adults in England are now overweight or obese[3].
- The overall cost of obesity to wider society was projected to reach £27 billion in 2015[4].

Barriers to healthy eating specifically reported by health care staff [13,14,15,16,17]

- Lack of time;
- Unpredictable/demanding workload;
- Prioritising patients workload;
- Inconvenient access to food (both physically ie: a long walk to where food is served, and in time ie: 24 hour duties but limited opening times.
- Lack of availability of food they want to eat.

The importance to the NHS

- The NHS is the fifth largest employer on the planet, and the largest employer in the UK[5]. Improving the health and wellbeing of the 1.4 million NHS staff will lift the health and wellbeing levels of the entire UK population.
- Obesity is associated with absenteeism, presenteeism, early retirement, injuries, discrimination, and litigation[6,7,8]. NHS staff absence due to poor health costs at least £2.4billion a year[9]. Improving employee health will improve productivity and performance of the workforce leading to improved patient care.
- Health professionals play an important role in health promotion for the 1 million patients seen in the NHS every 36 hours. Many staff feel more able to do this when they appear to be following health advice themselves[10,11]. By taking action to address diet and obesity for NHS staff, the organisation and staff will be role models for others.
- Policy drivers include NICE Guidance on obesity prevention in workplaces (CG43); NHS standard contract; NHS CQUIN Indicator: Healthy food for NHS staff, visitors and patients; NHS England voluntary sugar-sweetened beverage target [Appendix 1].

Evidence based environmental changes that work to improve diets

Changing the environment to promote healthy behaviour- such as healthy food and drink purchasing and consumption can be particularly useful and effective because:

- When the environment makes healthy choices easier, it doesn't require any effort by the target audience. This means it isn't just those people who are highly motivated to make healthy changes who benefit.
- Most changes are no-cost or low cost to introduce.

Providing information alone (for example, labelling food to indicate that it is healthy or its calorie content) is unlikely to improve diet, although this can work alongside other environmental changes.

More benefits to making these changes

- Greater availability of healthy food can improve the general attractiveness of a food provider[12].Customer volume and sales may increase after changes to improve healthy food and drink purchasing are made[13,14] Types of change for which there is evidence of effectiveness:

For peer review only

Type of change	Examples
Reduce the effort required to select healthy options, or increase the effort required to select unhealthy options.	➢ Healthier vegetable spread available in easy-to-reach positions, butter stored in a fridge.<br>➢ Desserts arranged so that fresh fruit salad was situated in the easiest access positions, cakes and pies in the least convenient.<br>➢ Rearrangement of the canteen to increase the prominence of fruit and water and reduce prominence of confectionery and crisps.
Increase availability of healthy options.	➢ Snacks on offer at a hospital canteen enforced at 75% healthy, no more than 25% unhealthy.<br>➢ Healthy snack cart that visited staff.
Offer smaller sizes of main meals and other products.	➢ Two size options for the main meal offered in a hospital canteen.

Other examples of successful initiatives include:

- An online pre-ordering system for lunch, which allowed staff to choose their lunch an hour or more before collecting it at the canteen without joining a queue. Nutritional information was presented and the system defaulted to the healthier option when appropriate.
- Traffic light labelling of all items on offer, with green (healthy) items placed in the most salient positions and red (unhealthy) items made less visible. Permanent changes to menu boards and other signage to highlight the healthiest options.

References

For more details of the systematic review that supported this document, please contact o.r.o.oyebode@warwick.ac.uk.

1. Newton JN, Briggs AD, Murray CJ, et al. Changes in health in England, with analysis by English regions and areas of deprivation, 1990–2013: a systematic analysis for the Global Burden of Disease Study 2013. *The Lancet* 2015;386:2257-74.
2. Scarborough P, Bhatnagar P, Wickramasinghe KK, et al. The economic burden of ill health due to diet, physical inactivity, smoking, alcohol and obesity in the UK: an update to 2006–07 NHS costs. *J Public Health* 2011;33:527-35.
3. Health and Social Care Information Centre, 2016. Health Survey for England 2015: Adult overweight and obesity. NHS Digital. https://digital.nhs.uk/catalogue/PUB20562 (accessed November 2017).
4. Butland B, Jebb S, Kopelman P, et al. Tackling obesities: future choices-project report. London: Department of Innovation, Universities and Skills 2007; 10:17.
5. Young A. Innovation within a national health care system. *Surgery* 2017;161:1179-82.
6. Shrestha N, Pedisic Z, Neil-Sztramko S, Kukkonen-Harjula KT, Hermans V. The impact of obesity in the workplace: a review of contributing factors, consequences and potential solutions. *Current obesity reports* 2016;5:344-60.
7. Koepp GA, Snedden BJ, Levine JA. Workplace slip, trip and fall injuries and obesity. *Ergonomics* 2015;58:674-79.
8. Flint SW, Snook J. Disability discrimination and obesity: the big questions? *Current obesity reports* 2015;4:504-09
9. Blake H, Mo PK, Lee S, Batt ME. Health in the NHS: lifestyle behaviours of hospital employees. *Perspectives in public health* 2012;132:213.
10. Dalton M. Implementing NICE obesity guidance for staff: an NHS trust audit. *Occup Med* 2014;65:78-85.
11. Van Kleef E, Otten K, van Trijp HC. Healthy snacks at the checkout counter: A lab and field study on the impact of shelf arrangement and assortment structure on consumer choices. *BMC public health* 2012;12:1072.
12. Jilcott Pitts S, Graham J, Mojica A, et al. Implementing healthier foodservice guidelines in hospital and federal worksite cafeterias: barriers, facilitators and keys to success. J Hum Nutr Diet 2016;29:677-86.
13. Kimathi AN, Gregoire MB, Dowling RA, Stone MK. A healthful options food station can improve satisfaction and generate gross profit in a worksite cafeteria. J Am Diet Assoc 2009;109:914-7

For peer review only

For peer review only

Reporting checklist for systematic review and meta-analysis.

Based on the PRISMA guidelines.

Instructions to authors

Complete this checklist by entering the page numbers from your manuscript where readers will find each of the items listed below.

Your article may not currently address all the items on the checklist. Please modify your text to include the missing information. If you are certain that an item does not apply, please write "n/a" and provide a short explanation.

Upload your completed checklist as an extra file when you submit to a journal.

In your methods section, say that you used the PRISMA reporting guidelines, and cite them as:

Moher D, Liberati A, Tetzlaff J, Altman DG, The PRISMA Group. Preferred Reporting Items for Systematic Reviews and Meta-Analyses: The PRISMA Statement

		Reporting Item	Page Number
	#1	Identify the report as a systematic review, meta-analysis, or both.	1
Structured summary	#2	Provide a structured summary including, as applicable: background; objectives; data sources; study eligibility criteria, participants, and interventions; study appraisal and synthesis methods; results; limitations; conclusions and implications of key findings; systematic review registration number	2
Rationale	#3	Describe the rationale for the review in the context of what is already known.	3
Objectives	#4	Provide an explicit statement of questions being addressed with reference to participants, interventions, comparisons, outcomes, and study design (PICOS).	4
Protocol and registration	#5	Indicate if a review protocol exists, if and where it can be accessed (e.g., Web address) and, if available, provide registration information including the registration number.	1,4

For peer review only

Eligibility criteria	#6	Specify study characteristics (e.g., PICOS, length of follow-up) and report characteristics (e.g., years considered, language, publication status) used as criteria for eligibility, giving rational	4-5
Information sources	#7	Describe all information sources in the search (e.g., databases with dates of coverage, contact with study authors to identify additional studies) and date last searched.	5
Search	#8	Present full electronic search strategy for at least one database, including any limits used, such that it could be repeated.	Supplementary 1
Study selection	#9	State the process for selecting studies (i.e., for screening, for determining eligibility, for inclusion in the systematic review, and, if applicable, for inclusion in the meta-analysis).	5
Data collection process	#10	Describe the method of data extraction from reports (e.g., piloted forms, independently by two reviewers) and any processes for obtaining and confirming data from investigators.	5
Data items	#11	List and define all variables for which data were sought (e.g., PICOS, funding sources), and any assumptions and simplifications made.	Supplementary 2
Risk of bias in individual studies	#12	Describe methods used for assessing risk of bias in individual studies (including specification of whether this was done at the study or outcome level, or both), and how this information is to be used in any data synthesis.	5
Summary measures	#13	State the principal summary measures (e.g., risk ratio, difference in means).	5
Planned methods of analyis	#14	Describe the methods of handling data and combining results of studies, if done, including measures of consistency (e.g., I2) for each meta-analysis.	5
Risk of bias across studies	#15	Specify any assessment of risk of bias that may affect the cumulative evidence (e.g., publication bias, selective reporting within studies).	25-29

For peer review only

Additional analyses	#16	Describe methods of additional analyses (e.g., sensitivity or subgroup analyses, meta-regression), if done, indicating which were pre-specified.	5
Study selection	#17	Give numbers of studies screened, assessed for eligibility, and included in the review, with reasons for exclusions at each stage, ideally with a flow diagram.	6, PRISMA flow
Study characteristics	#18	For each study, present characteristics for which data were extracted (e.g., study size, PICOS, follow-up period) and provide the citation.	14-20
Risk of bias within studies	#19	Present data on risk of bias of each study and, if available, any outcome-level assessment (see Item 12).	25-29
Results of individual studies	#20	For all outcomes considered (benefits and harms), present, for each study: (a) simple summary data for each intervention group and (b) effect estimates and confidence intervals, ideally with a forest plot.	21-22
Synthesis of results	#21	Present the main results of the review. If meta-analyses are done, include for each, confidence intervals and measures of consistency.	6-9
Risk of bias across studies	#22	Present results of any assessment of risk of bias across studies (see Item 15).	9
Additional analysis	#23	Give results of additional analyses, if done (e.g., sensitivity or subgroup analyses, meta-regression [see Item 16]).	22
Summary of Evidence	#24	Summarize the main findings, including the strength of evidence for each main outcome; consider their relevance to key groups (e.g., health care providers, users, and policy makers	10,11
Limitations	#25	Discuss limitations at study and outcome level (e.g., risk of bias), and at review level (e.g., incomplete retrieval of identified research, reporting bias).	10
Conclusions	#26	Provide a general interpretation of the results in the context of other evidence, and implications for future research.	10
Funding	#27	Describe sources of funding or other support (e.g., supply	12-13

For peer review only

of data) for the systematic review; role of funders for the systematic review.

The PRISMA checklist is distributed under the terms of the Creative Commons Attribution License CC-BY. This checklist can be completed online using https://www.goodreports.org/, a tool made by the EQUATOR Network in collaboration with Penelope.ai

For peer review only
